# Red Blood Cell Membrane Lipidomics: Potential Biomarkers Detecting Method for Plasma Volume Overload and Major Adverse Cardiovascular Events in Chronic Heart Failure Patients

**DOI:** 10.1002/advs.202502893

**Published:** 2025-06-23

**Authors:** Lin Zhang, Xiangqin Ou, Jingyi Lin, Xiaofei Luo, Jiashun Zhou, Xingyue Zhou, Peihua Zhao, Li Liu, Ziran Zhao, Ying Zhou, Guanwei Fan, Lifeng Han, Xiumei Gao

**Affiliations:** ^1^ Medical Experiment Center First Teaching Hospital of Tianjin University of Traditional Chinese Medicine Tianjin 300381 China; ^2^ Medical Experiment Center National Clinical Research Center for Chinese Medicine Acupuncture and Moxibustion Tianjin 300381 China; ^3^ State Key Laboratory of Component‐based Chinese Medicine Tianjin University of Traditional Chinese Medicine Tianjin 301617 China; ^4^ Jinghai District Hospital of Tianjin University of Traditional Chinese Medicine Tianjin 301600 China

**Keywords:** chronic heart failure, erythrocyte stress index, major adverse cardiovascular events, plasma volume overload, red blood cell membrane lipidomics

## Abstract

Plasma volume fluctuations limit the utility of circulating lipidomics in chronic heart failure (CHF). In contrast, red blood cell (RBC) membrane lipidomics may serve as stable biomarkers that are unaffected by plasma volume changes. Two cohorts are included to investigate the association between RBC indicators and CHF. RBC membrane lipidomics is first used to characterize CHF and its impact on plasma volume overload (PVO) and major adverse cardiovascular events (MACE). The first cohort (n = 507,638) shows that the erythrocyte stress index (ESI), better than traditional RBC indicators, is associated with the prevalence of CHF with odds ratio (OR) and confidence interval (CI) of 1.57 (95% CI,1.55‐1.59). ESI is also linked with in‐hospital death in CHF. Another cohort (n = 1,550) indicated RBC membrane lipidomics ceramide subtype Cer 18:0;O2/16:0 and lysophosphatidylethanolamine subtype LPE 18:0 has an AUC of PVO with 0.75 and 0.61. The above two lipids are risk factors of PVO with OR of 1.62 (95% CI, 1.47‐1.80) and 1.11 (95% CI, 1.06‐1.16). They also are risk factors for MACE, with hazard ratios (HR) of 1.04 (95% CI, 1.01‐1.07) and 1.06 (95% CI, 1.01‐1.10). This research emphasizes the potential value of RBC membrane lipidomics for CHF development, prognosis, and treatment.

## Graphical abstract

A novel RBC indicator, ESI, is better than the traditional RBC indicators and is associated with CHF prevalence and in‐hospital death. RBC membrane lipidomics, Cer 18:0;O2/16:0, and LPE 18:0, are associated with plasma volume overload and MACE in CHF patients, but these plasma lipids could not.

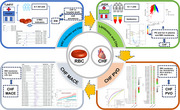

## 1 Introduction

Lipidsomics systematically investigates the composition, structure, and function of lipids and reveals their roles in both physiological and pathological contexts. Lipid metabolism disorder is a significant clinical feature of CHF,^[^ ^1^ ^]^ affecting both cardiac^[^ ^2^ ^]^ and circulation systems.^[^ ^3^ ^]^ Even though lipidomics can be classified into spatial lipidomics (spectrometry imaging),^[^ ^4^ ^]^ structural lipidomics (details of lipid structures),^[^ ^5^ ^]^ and clinical lipidomics (diagnosis or drug targets.^[^ ^6^ ^]^ Currently, most studies of CHF focus on circulating lipidomics (e.g., serum and plasma)^[^ ^3^, ^7^ ^]^ in clinical lipidomics.

Plasma volume has a significant impact on circulating lipids. For instance, serious diarrhea or vomiting can lead to hemoconcentration, resulting in hyperglycemia.^[^ ^8^ ^]^ In CHF, cardiac insufficiency increases plasma volume and might cause dilutional anemia.^[^ ^9^ ^]^ Therefore, the plasma volume increase or decrease will influence the concentration of the circulating lipid. Severe volume fluctuations in CHF patients limit the utility of circulation lipidomics. Due to decreased cardiac function and clinical interventions, plasma volume in CHF patients can be divided into two stages: plasma volume overload (PVO) during the onset stage and reduced volume overload following intervention.^[^ ^10^ ^]^ Drugs recommended by CHF guidelines,^[^ ^11^ ^]^ such as sodium‐glucose transporter 2 inhibitors, diuretics, and insulin, can impact circulating volume, indirectly affecting lipid concentrations. Therefore, identifying a stable source for lipidome detection is crucial.

Red blood cell (RBC) membrane lipidomics holds promise as a diagnostic tool for CHF for four reasons. First, RBCs have a lifespan of ≈120 days, significantly longer than other blood cells (7–10 days for platelets and 7–14 days for white blood cells). Second, the RBC membrane is rich in lipids,^[^ ^12^ ^]^ primarily composed of phospholipids, cholesterol, and glycolipids. Third, lipids on the RBC membrane remain stable because they are firmly bound to the membrane and are not easily displaced.^[^ ^13^ ^]^ Lastly, CHF is often accompanied by RBC dysfunction, leading to vascular endothelial injury.^[^ ^14^ ^]^ Thus, RBCs represent a potential target for cardiovascular disease (CVD) prevention and treatment,^[^ ^15^ ^]^ and exploring RBC membrane lipidomics may offer a more sensitive and precise approach to characterizing CHF.

This study examines the relationship among CHF, RBC membrane, and lipidomics. First, we established a cohort to investigate the association between RBC‐related clinical parameters and CHF prevalence and in‐hospital mortality. Subsequently, another cohort was used to explore the relationship among RBC membrane lipidomics, CHF plasma volume, and major adverse cardiovascular events (MACE).

## 2 Results

### 2.1 Association Between RBC Indicators, CHF's Prevalence, and CHF In‐Hospital Death

**Figure 1:**
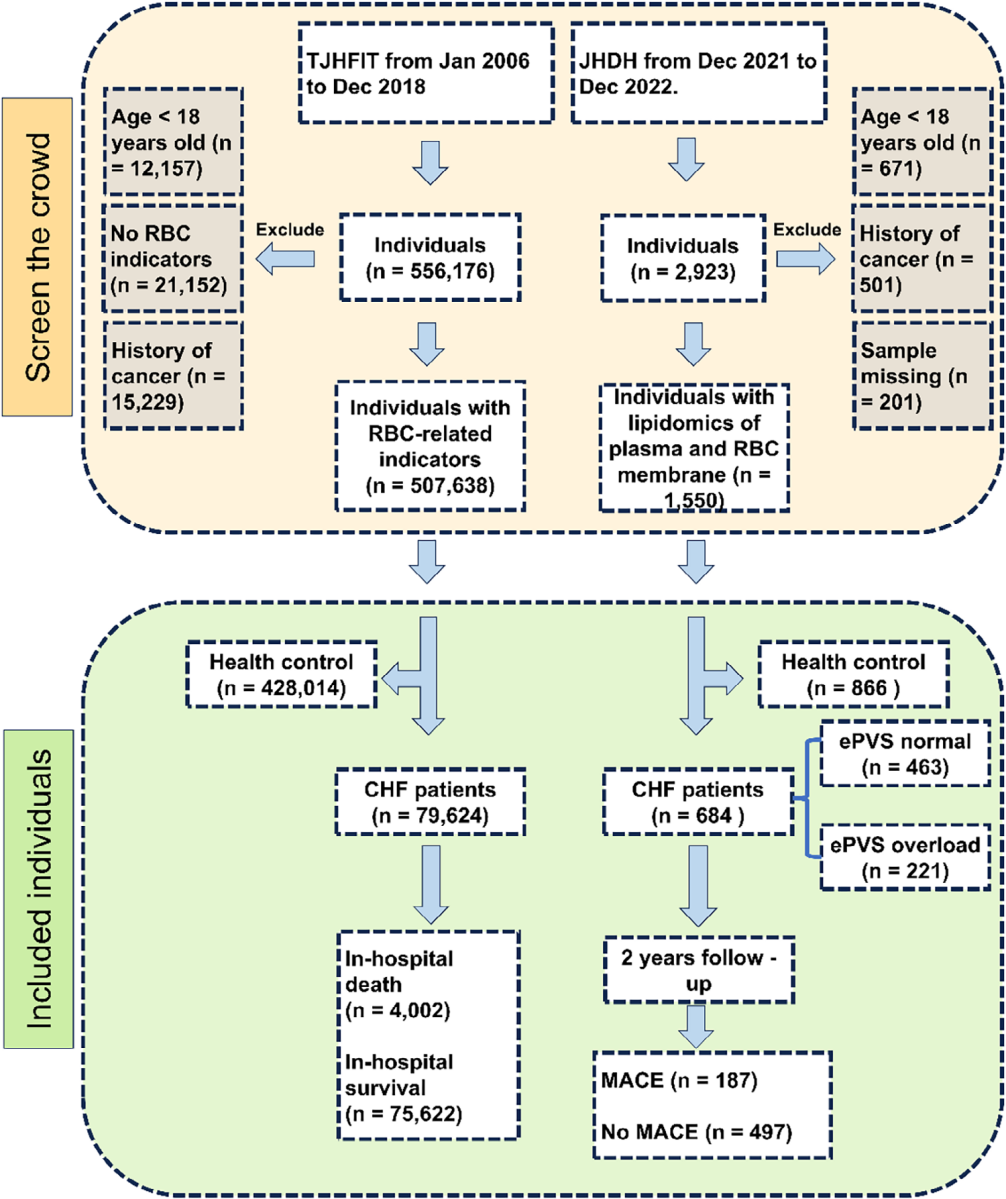
The flow chart of this study.

In Tianjin Heart Failure Integrated Treatment (TJHFIT) cohorts, 507,638 individuals with full eight RBC indicators were recruited, including 428,014 healthy controls (Con) and 79,624 CHF patients. Among these CHF patients, 4 002 died in the hospital, and 75 622 survived (**Figure** **1**). In Jinghai District Hospital (JHDH), 1,550 individuals were recruited, including 866 Con and 684 CHF patients. According to estimated plasma volume status (ePVS) > 5.5 or not, the CHF patients were classified into ePVS normal or ePVS overload (PVO patients). During the 2‐year follow‐up period, 187 individuals developed MACE, while 497 did not.

**Figure 2:**
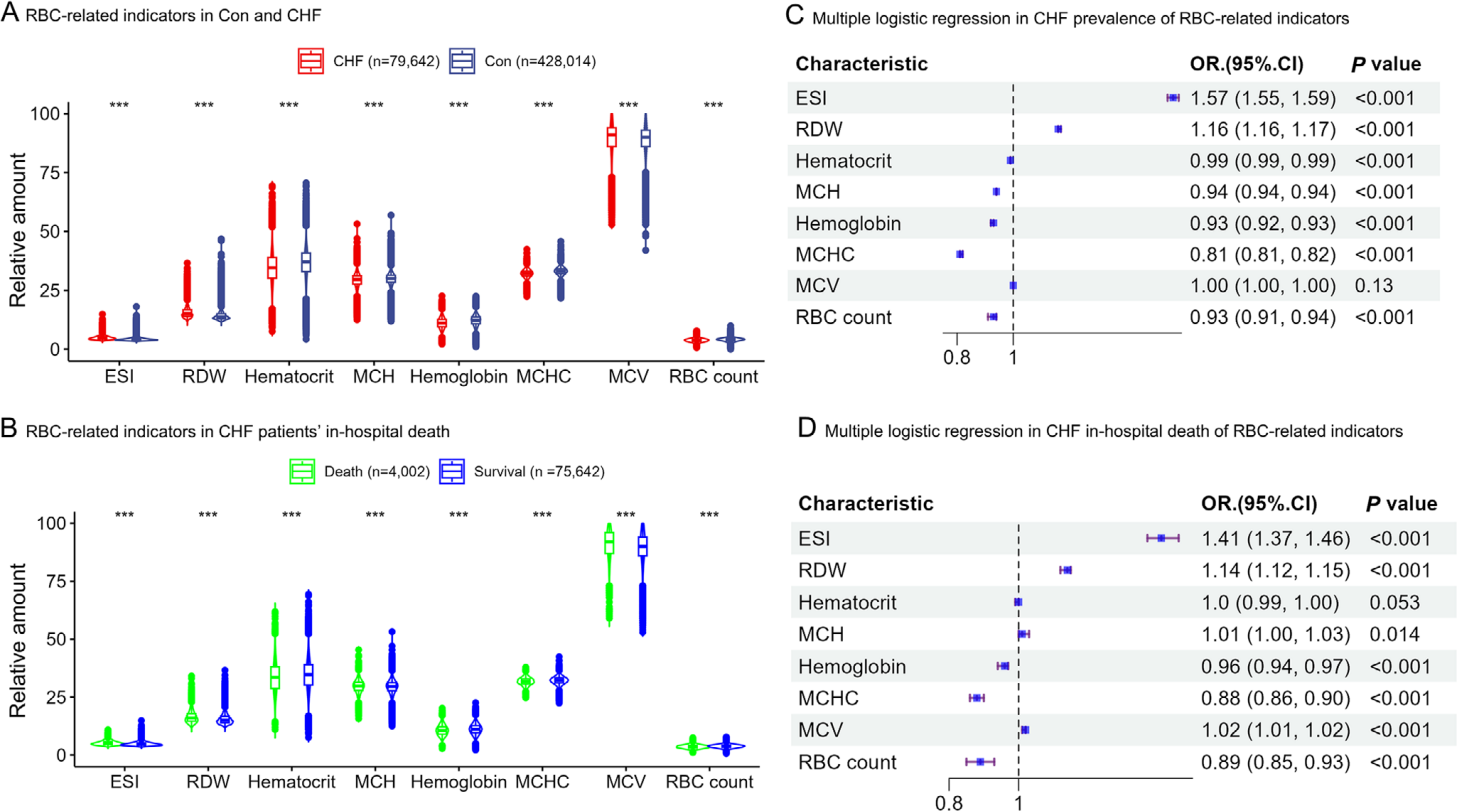
The RBC indicators for the CHF prevalence and in‐hospital death. A). RBC‐related indicators in Con and CHF B). RBC‐related indicators in CHF patients’ in‐hospital death C). Multiple logistic regression in the CHF prevalence of RBC‐related indicators D). Multiple logistic regression in CHF in‐hospital death of RBC‐related indicators.

The base characteristics of Con vs CHF in the TJHFIT are shown in Table S1 (Supporting Information). The baseline characteristics of CHF patients' deaths in the hospital are shown in Table S2 (Supporting Information). Compared with the Con group, the CHF patients had higher diabetes mellitus (DM), CVD, hyperlipidemia, chronic kidney diseases (CKD), atrial fibrillation (AF), nicotine dependence, anemia, hyperuricemia, obstructive sleep apnea, male, and age. In RBC indicators, the CHF patients had a higher erythrocyte stress index (ESI), red blood cell distribution width (RDW), and mean cell volume (MCV), lower RBC count, hematocrit, hemoglobin, mean hemoglobin content (MCH), and mean corpuscular hemoglobin concentration (MCHC) than the Con group (**Figure** **2**A). Similarly, the CHF patients who died in the hospital had a higher ESI, RDW, and MCV, lower RBC count, hematocrit, hemoglobin, MCH, and MCHC than the CHF survived in the hospital (Figure 2B). Multiple logistic regression (Table S3, Supporting Information) indicated that the ESI and RDW were independent risk factors of CHF, with odds ratios (OR) and 95% confidence interval (CI) of 1.57(1.55, 1.59) and 1.16(1.16, 1.17), respectively (Figure 2C). The MCHC, MCH, RBC count, hemoglobin, and hematocrit were independent protective factors of CHF. More importantly, multiple logistic regression (Table S4, Supporting Information) indicated that the ESI and RDW had the top 2 highest OR among all RBC indicators, which were independent risk factors of death in hospitals with OR (95%CI) 1.41(1.37, 1.46) and 1.14(1.12, 1.15), respectively (Figure 2D). The MCHC, RBC count, hemoglobin, and hematocrit were independent protective factors of in‐hospitals death in CHF. In summary, the ESI had the highest OR in both CHF prevalence and CHF in‐hospital death.

### 2.2 Lipidomics Analysis in CHF Patients vs Con

To explore the association between lipidomics and CHF, 1, 550 individuals were recruited, including 866 Con and 684 CHF patients. Compared to the Con group, the CHF patients were older and had a higher proportion of males, as well as an increased prevalence of CVD, AF, DM, hyperlipidemia, hyperuricemia, CKD, hypertension, chronic obstructive pulmonary disease (COPD), and anemia (Table S5, Supporting Information). As for the RBC indicators, the CHF had a higher ESI, RDW, and lower RBC count, hematocrit, hemoglobin, and MCHC than the Con group.

**Figure 3:**
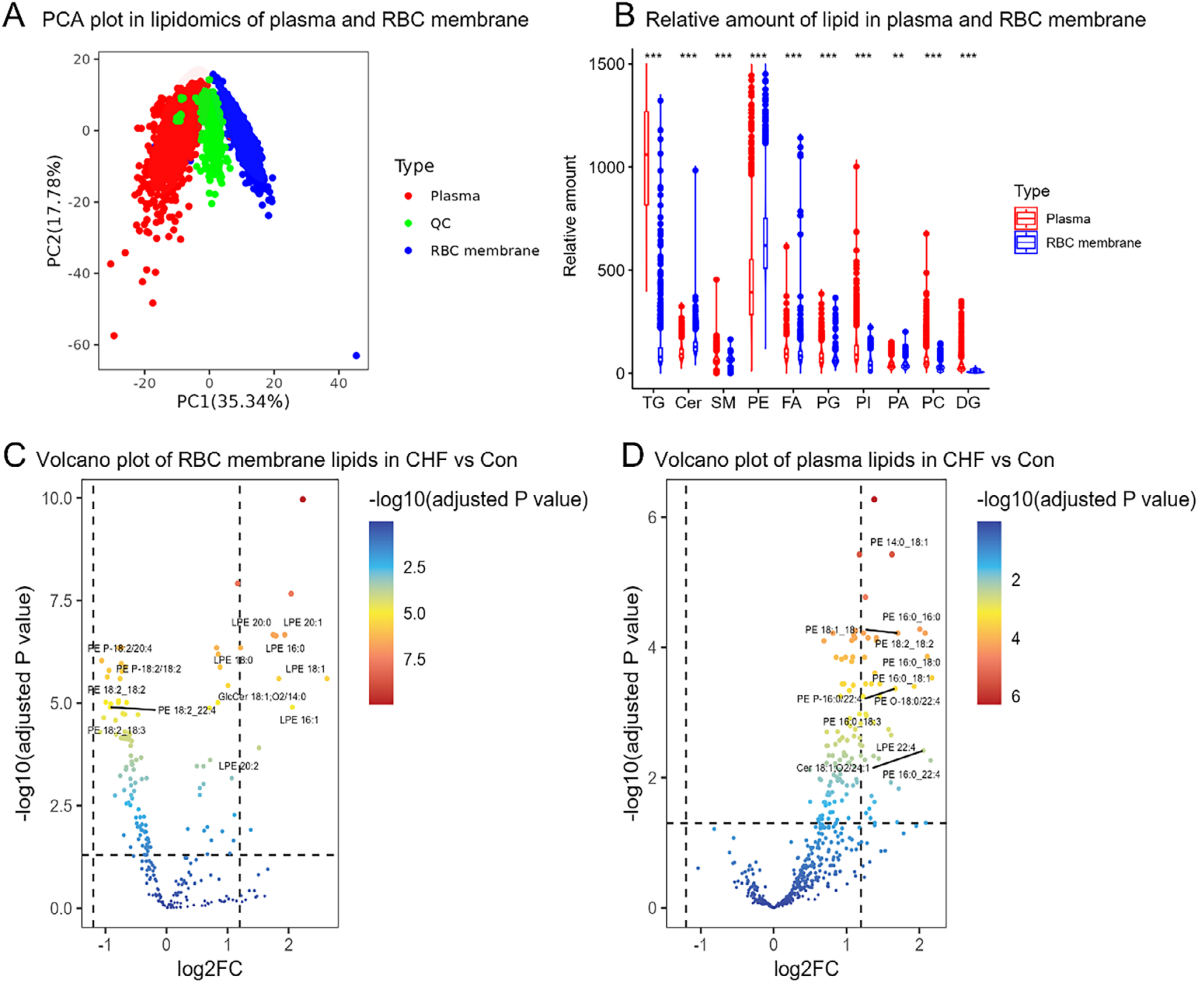
Differences between plasma and RBC membrane lipidomics. A). PCA plot in lipidomics of plasma and RBC membrane B). Relative amount of lipid in plasma and RBC membrane C). Volcano plot of RBC membrane lipids in CHF vs Con, and the plot only marks 13 subtypes of PE and Cer because of the limited typography D). Volcano plot of plasma lipids in CHF vs Con, and the plot only marks 12 subtypes due to the limited typography. (PCA, principal component analysis, QC, quality control, TG, triglycerides, Cer, ceramide, SM, sphingomyelin, PE, Phosphatidylethanolamine, FA, fatty acids, PG, phosphatidylglycerol, PI, phosphatidylinositol, PA, phosphatidic acid, PC, phosphatidylcholine, DG, diacylglycerol).

Additionally, the lipidomics analysis of plasma and RBC membranes was completed (**Figure** **3**). Finally, the type of RBC membrane and plasma lipids were 241 and 569. According to Figure 3A, the results of quality control (QC) samples fall in the middle of the plasma and RBC membrane, and the plasma and RBC membrane separate from each other, which indicates the data is reliable and the following analysis is credible. Next, the lipid subtypes were summarized (Figure 3B). The plasma triglycerides (TG), diglyceride (DG), and fatty acids (FA) were significantly higher than the RBC membrane. RBC membrane phosphatidylethanolamine (PE) and ceramide (Cer) were significantly higher than in plasma. Although the other lipids were also different between plasma and RBC membranes, they were not as high as those mentioned above.

Focusing on PEs and ceramides, the RBC membrane indicated 27 different lipids in CHF compared to Con (absolute value of log2 fold change >1 and adjusted P value < 0.05), including 4 ceramides, 7 lysophosphatidylethanolamines (LPEs), and 16 PEs (Table S6, Supporting Information). Among the 4 ceramides, CHF patients had a higher Cer 18:0;O2/16:0, GlcCer 18:1;O2/14:0, GlcCer 18:1;O2/16:0, but lower GlcCer 18:0;O2/18:0 compared to Con. All 7 LPEs were increased in CHF patients. Except for PE P‐16:0/16:1, all other PEs were decreased in CHF patients. Volcano plot (Figure 3C) indicated the GlcCer 18:1;O2/14:0, LPE 16:0, LPE 20:1, and so on were higher in the CHF patient's RBC membrane than the Con. Overall, CHF patients had a higher LPE, Cer, and lower PE in the RBC membrane compared to the Con group.

As for the plasma lipid, 43 different lipids were identified in CHF vs Con, including 4 ceramides, 10 LPEs, and 29 PEs (Table S7, Supporting Information). Interestingly, the plasma PE indicated an opposite trend compared with the RBC membrane (Figure 3D).

Four models of logistic regression (Tables S8 and S9, Supporting Information) were applied. In the RBC membrane lipids (Table S8, Supporting Information), except for GlcCer 18:0;O2/18:0, the remaining 3 Cers, and all LPEs were identified as risk biomarkers for CHF. In PEs, the PE P‐16:0/16:1 was a risk marker, and 6 PEs had no significance (PE 18:1_22:4, PE 18:2_18:2, PE 18:2_20:3, PE 18:2_20:4, PE 18:2_22:4, and PE P‐18:1/22:4), other PEs were protective markers for CHF. As for the plasma lipids (Table S9, Supporting Information), except Cer 18:1;O2/24:1 with insignificance, other lipids acted as risk factors for CHF.

### 2.3 The Association Between Different Lipids in CHF with ePVS Normal and Overloaded

**Figure 4:**
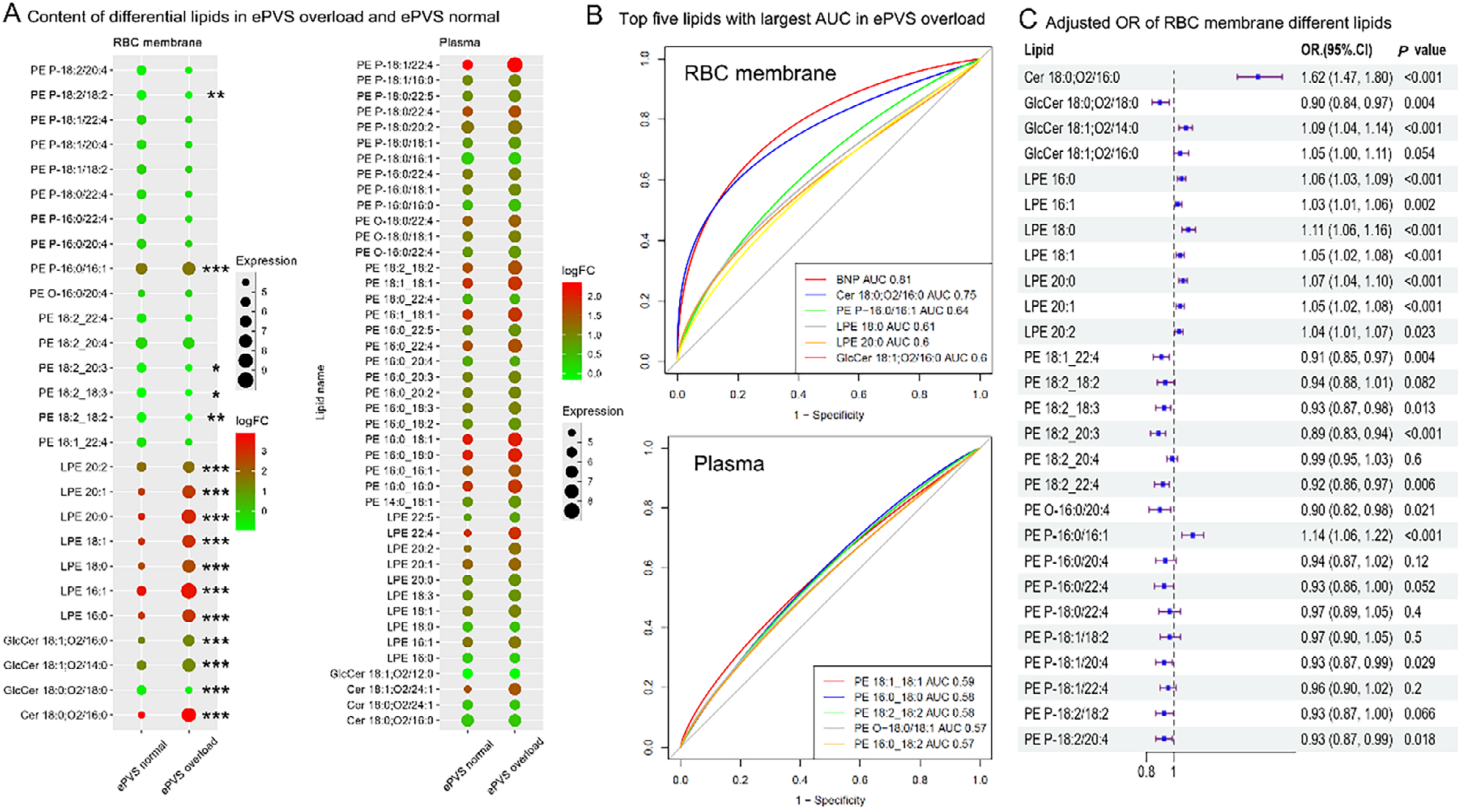
Comparison of lipids differing between plasma and RBC membranes in ePVS normal and ePVS overload CHF patients. A). Content of differential lipids in ePVS overload and ePVS normal, ^***^ mean adjusted *P* value < 0.001, ^**^ mean adjusted *P* value < 0.01, ^*^ mean adjusted *P* value < 0.05, nothing mean adjusted P value > 0.05 B). The top five lipids with the largest AUC in ePVS overload C). Adjusted OR of RBC membrane different lipids.

Among the 684 CHF patients, 463 had normal ePVS, while 221 experienced ePVS overload (Table S10, Supporting Information). Examining the lipids that differed between plasma and RBC membrane, 16 RBC membrane lipids were significantly altered between patients with normal ePVS and those with ePVS overload (**Figure** **4**A). Compared with ePVS normal, the ePVS overload patients had a higher Cer 18:0;O2/16:0, GlcCer 18:1;O2/14:0, GlcCer 18:1;O2/16:0, and lower GlcCer 18:0;O2/18:0. All subtypes of LPEs increased in the ePVS overload CHF patients, including LPE 16:0, LPE 16:1, LPE 18:0, LPE 18:1, LPE 20:0, LPE 20:1, and LPE 20:2. Except for PE P‐16:0/16:1 significantly increased in ePVS overload, four PEs decreased significantly, including PE 18:2_18:2, PE 18:2_18:3, PE 18:2_20:3, and PE P‐18:2/18:2.

In contrast, none of the plasma lipids showed significant associations. The receiver operating characteristic curve (ROC) curve revealed that the BNP had the highest area under the curve (AUC) at 0.81 (Figure 4B), followed by RBC membrane lipids Cer 18:0;O2/16:0 (AUC = 0.75), PE P‐16:0/16:1 (AUC = 0.64), LPE 18:0 (AUC = 0.61), LPE 20:0 (AUC = 0.6), and GlcCer 18:1;O2/16:0 (AUC = 0.6), while none of the plasma lipids showed AUC higher than 0.6. In the multiple logistic regression, we adjusted the RBC membrane lipids with four models (Table S11, Supporting Information) to further explore the association between RBC membrane different lipids and ePVS overload. The adjusted OR of RBC different lipids (Figure 4C) revealed that the top 3 highest OR were observed for Cer 18:0;O2/16:0 (1.62; 95% CI, 1.47‐1.80), PE P‐16:0/16:1 (1.14; 95% CI, 1.06, 1.22), and LPE 18:0 (1.11; 95% CI, 1.06, 1.16).

### 2.4 The Association of Lipids with MACE in CHF Patients

Over 30 monthly follow‐up periods, 187 CHF patients experienced MACE, while the rest 497 did not (Table S12, Supporting Information). Cox proportional hazards model was applied (Table S13, Supporting Information) to explore the association between different lipids and MACE. The plasma lipids showed no significance in the adjusted hazard ratio (HR), except for GlcCer 18:1;O2/12:0 showing an HR of 1.03 (95 CI%, 1.01–1.07).

**Figure 5:**
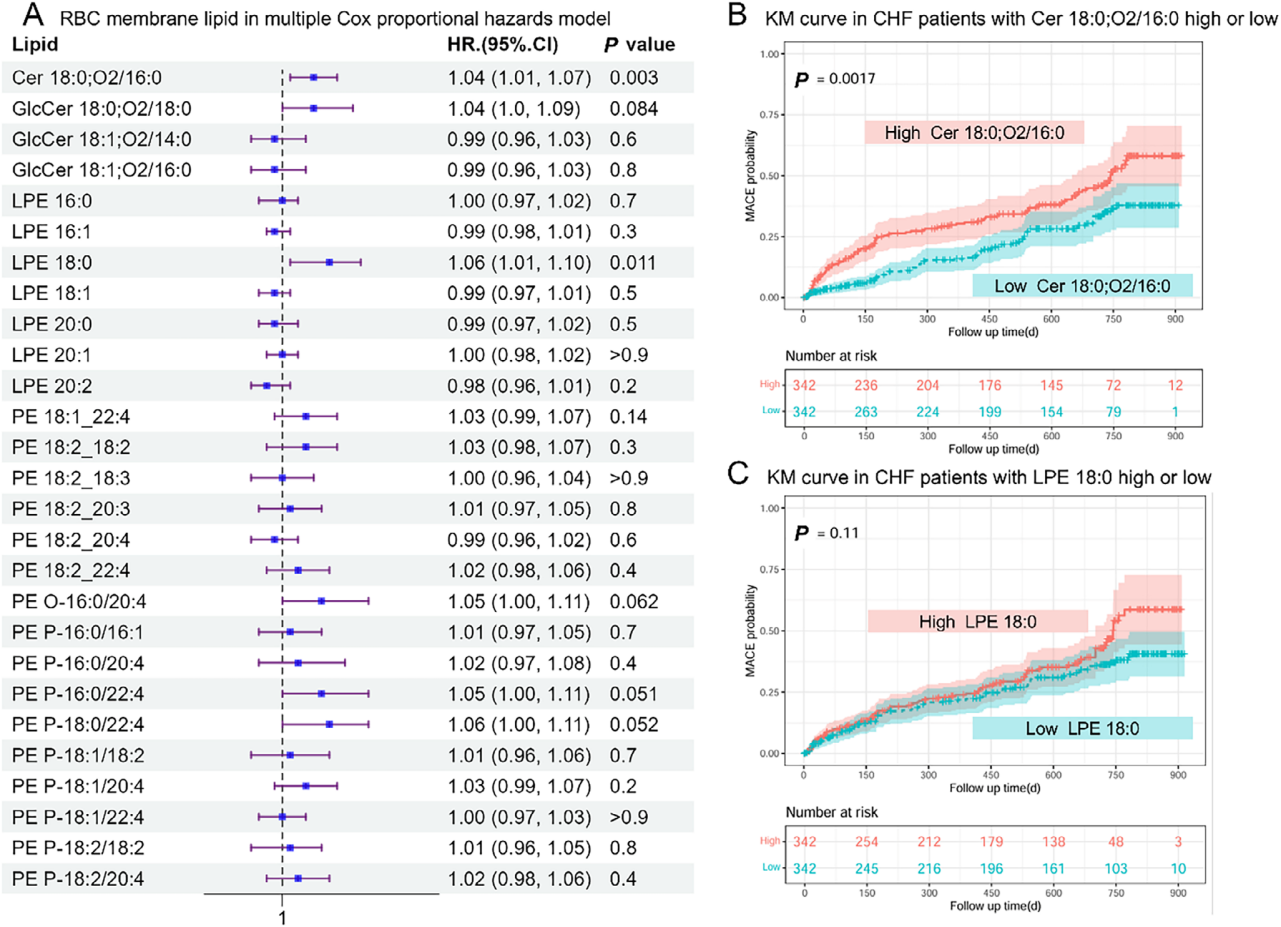
Association of MACE with differential lipids in the RBC membranes of CHF. A). RBC membrane lipid in multiple Cox proportional hazards model B). KM curve in CHF patients with Cer 18:0;O2/16:0 high or low C). KM curve in CHF patients with LPE 18:0 high or low. (KM, Kaplan‐Meier).

The adjusted HR (**Figure** **5**A) in RBC membrane lipid indicated both Cer 18:0;O2/16:0 and LPE 18:0 had a significant adjusted HR (95 CI%) of 1.04 (1.01, 1.07), 1.06 (1.01, 1.10), respectively. Using the median relative content value as a cut‐off, 4.69 in Cer 18:0;O2/16:0, and 4.86 in LPE 18:0, CHF patients were classified into high or low groups. The Kaplan‐Meier curve (Figure 5B) revealed the high Cer 18:0;O2/16:0 group had a significantly increased probability of MACE compared to the low Cer 18:0;O2/16:0 group, but LPE 18:0 did not.

### 2.5 Morphology of RBC and Correlation Between Lipid Subgroups

**Figure 6:**
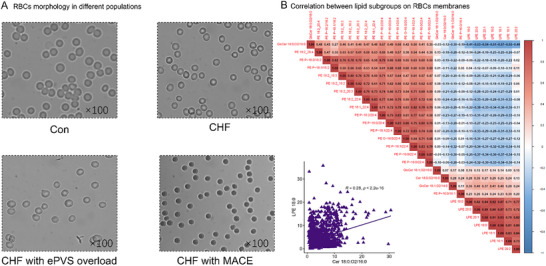
Morphology of RBCs as well as correlation between lipid subgroups A). RBCs morphology in different populations B). Correlation between lipid subgroups on RBCs membranes, and the scatter plot between LPE 18:0 and Cer 18:0;O2/16:0 in RBC membranes.

The morphology of RBCs varies greatly among different populations (**Figure** **6**). In Con (Figure 6A), the RBCs are nearly perfectly round, with a clear biconcave disc shape. In patients with CHF, the biconcave discs of some RBCs were not visualized, and the RBCs were of variable size. In ePVS overload CHF, the individual variation of RBCs is exacerbated, the shape of some RBCs becomes irregular, and the number of RBCs is reduced in the visual field, probably because of volume dilution. In CHF patients with MACE, the volume of the RBCs was significantly shrunken, and the edge of the red blood cells showed spiculated spinous processes.

The correlation analysis of RBC membrane lipids (Figure 6B) in the 1,550 volunteers showed that the correlation was strong for the same subtype, but weak for different subtypes, and there was a significant negative correlation between LPE and PE subtypes. For example, the coefficient between LPE 18:1 and LPE 20:2 was 0.84, and the correlation coefficient between LPE 18:1 and PE P‐18:0/22:4 was ‐0.37. In addition, the LPE 18:0 and Cer 18:0; O2/16:0 was positively correlated (*R* = 0.28).

## 3 Conclusion and Discussion

RBC indicators, especially the ESI, were associated with CHF prevalence and in‐hospital death. RBC membrane ceramide, PE, and LPE can independently predict CHF ePVS overload and MACE more effectively than plasma, especially Cer 18:0;O2/16:0. RBC membrane lipids demonstrate significant potential as novel biomarkers and therapeutic targets for CHF, offering new avenues for future precise intervention and personalized treatment strategies.

Previous studies focus on lipidomics in RBC,^[^ ^16^ ^]^ but few on RBC membranes. RBC FA^[^ ^17^ ^]^ and cholesterol^[^ ^18^ ^]^ have received the most attention among all lipid types. For instance, omega‐3 fatty acids, especially docosahexaenoic acid and alpha‐linolenic acid, have been verified as protective markers for various conditions, including adolescents' attention,^[^ ^16^ ^]^ age‐related macular degeneration,^[^ ^19^ ^]^ and cognitive function.^[^ ^20^ ^]^ Conversely, a higher percentage of RBC arachidonic acid is related to obesity,^[^ ^21^ ^]^ Alzheimer's Disease,^[^ ^22^ ^]^ and RBC membrane fluidity.^[^ ^23^ ^]^ However, other RBC lipids received less attention than the above lipids, such as ceramide. Ceramides are key components of RBC membranes, playing a significant role in maintaining cellular integrity, regulating membrane dynamics, and modulating cellular signaling pathways.^[^ ^24^ ^]^ Our study emphasized new light on other RBC membrane lipids in CHF, especially ceramides and PE.

The abnormal composition of RBC membrane lipids in CHF patients may be related to RBC oxidative stress level. First, PE^[^ ^25^ ^]^ and ceramide^[^ ^26^ ^]^ were important guarantees for RBC to maintain membrane fluidity. Elevated levels of reactive oxygen species (ROS)^[^ ^27^ ^]^ in CHF patients can attack the RBC membrane,^[^ ^28^ ^]^ leading to lipid peroxidation, which disrupts membrane fluidity and stability.^[^ ^14^ ^]^ Second, ROS may inhibit the activity of membrane repair enzymes, such as phospholipase A2,^[^ ^29^ ^]^ resulting in the accumulation of abnormal lipids, such as oxidized phospholipids, further altering the membrane structure. Therefore, the accumulation of abnormal lipid peroxidation (e.g., LPE) impairs RBC membrane fluidity, increasing RBC rigidity.^[^ ^30^ ^]^ This compromises the ability of RBC to traverse narrow capillaries, thereby exacerbating myocardial tissue hypoxia.^[^ ^31^ ^]^

RBC membrane lipidomics holds potential applications in diseases with significant plasma volume fluctuations (increase or decrease). Although circulating lipidomics can be utilized in CHF,^[^ ^3^ ^]^ diabetes,^[^ ^32^ ^]^ obesity,^[^ ^33^ ^]^ and so on, few studies have focused on the impact of circulating lipids on plasma volume due to the dilution or concentration by plasma. As evidenced in our results (Figure 4), where the differences in the plasma lipids were effectively diluted. Therefore, RBC membrane lipidomics not only holds potential for applications in CHF but also offers promising prospects for diseases with significant plasma volume fluctuations, including diabetic ketoacidosis,^[^ ^34^ ^]^ chronic kidney disease,^[^ ^35^ ^]^ burns,^[^ ^36^ ^]^ and so on.

Previous studies indicated that circulating ceramide is associated with better prognosis in CHF,^[^ ^37^ ^]^ such as Cer 18:1;O2/24:0.^[^ ^37^ ^]^ Even though plasma Cer 18:1;O2/16:0 is a risk factor for CHF,^[^ ^3^ ^]^ plasma Cer 18:1;O2/16:0 between the ePVS overload or MACE has never been reported. Higher plasma ceramides might be associated with cardiovascular mortality but associations are weakened after adjustment for established cardiovascular risk factors, such as plasma NT‐proBNP.^[^ ^38^ ^]^ Our findings revealed that four plasma ceramides are associated with CHF, however, this association is weakened when ePVS overloads with the dilution effect of concentration. Notably, as shown in our results, in the case of the ePVS overload, plasma ceramides and plasma PE demonstrated poor predictive value, but RBC membrane ceramides remain reliable. More importantly, in CHF‐related MACE, RBC membrane Cer 18:1;O2/16:0 is a stable predictive marker across all Cox proportional hazards models, underscoring its potential as a robust biomarker.

Another interesting finding in this study is the opposite trend of PE levels in plasma and RBC membranes in CHF. Briefly, CHF patients show decreased PE in RBC membranes, but elevated levels in plasma, just like previous research in RBC^[^ ^12^ ^]^ and plasma lipidomics.^[^ ^39^ ^]^ In cardiovascular diseases, there were consistent with our findings, elevated plasma LPE,^[^ ^40^ ^]^ PE, and ceramide.^[^ ^41^ ^]^ This discrepancy may be because of the different sources of PE. For instance, endothelial cells^[^ ^42^ ^]^ enriched PE, and CHF are always accompanied by vascular endothelial damage.^[^ ^43^ ^]^ This leads to the release of PE into the blood and results in an elevation of plasma PE. Damaged cardiomyocytes^[^ ^44^ ^]^ will increase PE and then secrete it into the plasma. CHF is always associated with liver dysfunction,^[^ ^45^ ^]^ which will increase PE generation in the liver.^[^ ^46^ ^]^ In RBC, the ROS of CHF excessively activates phospholipase A2,^[^ ^47^ ^]^ thereby promoting the hydrolysis of PE to LPE,^[^ ^48^ ^]^ which might explain why the RBC membrane PE decreased but LPE increased. Additionally, the ROS can target unsaturated fatty acids^[^ ^49^ ^]^ in PE, resulting in its degradation into LPE. In summary, the plasma PE increase might be the multiple tissue sources, and the RBC membrane PE decrease might be the phospholipase A2 hydrolysis or ROS degraded to LPE.

This study has third limitations. First, logistic regression analysis, often used in cross‐sectional studies, can only identify associations between variables,^[^ ^50^ ^]^ not causal relationships. Even though this study verified the association between RBC membrane lipid and CHF PVO, the causal relationships need further exploration in a randomized control trial. Second, we considered various confounding factors to bring our estimate closer to the true association in randomized control trials, there are still many that we did not take, e.g., diet, sleep, and other lifestyle factor. Third, the validation of another cohort is necessary to better explore the robustness of the results. However, replicating the entire cohort proved to be challenging due to several factors: collection of RBC membranes is complicated (ethical considerations and dedicated personnel to process blood samples daily), along with the collection of clinical data, and the RBC lipidomics testing at the 30‐month follow‐up period. To ensure the robustness of our results, we developed a clinically applicable RBC membrane lipidomics assay, which streamlined the extraction process by eliminating lymphocyte separation medium, thereby reducing operational errors. Deuterated internal standards and quality control samples were employed to ensure data accuracy. All samples were tested continuously using a well‐maintained UHPLC‐MS/MS to ensure robustness.

## 4 Experimental Section

### Participants

This study included two cohorts. The first was the Tianjin Heart Failure with Integrated Treatment (TJHFIT), which aims to explore the curative effects of integrated HF treatment in the Greater Tianjin Area. Participants were enrolled between January 1, 2006, and December 31, 2018, from 43 tertiary hospitals and 39 hospitals in Tianjin, China. TJHFIT was used to explore the association between RBC‐related indicators and CHF.

The second cohort was from Jinghai District Hospital (JHDH) of Tianjin, a partner of TJHFIT. To explore the relationship between RBC membrane lipidomics and CHF, volunteers were recruited from JHDH. All CHF patients and Con were recruited from December 2021 to December 2022. The study was approved by the ethics committee of JHDH under Plan 11 (Diary number: JHYYLL‐2022‐0307).

### Definition of CHF and Endpoints

According to the guidelines,^[^ ^11^, ^51^ ^]^ CHF was diagnosed based on a combination of clinical symptoms, B‐type natriuretic peptide (BNP), and left ventricular ejection fraction. Typically, clinical symptoms or signs (e.g., dyspnea and fatigue), a left ventricular ejection fraction (LVEF) of less than 50%, and a BNP level greater than 100 pg mL^−1^ confirm a diagnosis of CHF.

In the JHDH study, follow‐up concluded on November 5, 2024, unless an endpoint event (MACE) occurred earlier. The total MACEs^[^ ^52^ ^]^ included the following: (i) cardiac death or rehospitalization, including fatal coronary artery disease or myocardial infarction; (ii) unplanned revascularization; (iii) in‐stent restenosis; and (iv) stroke, including cerebral infarction, cerebral hemorrhage, and subarachnoid hemorrhage.

### Extraction and Detection of Lipids

Lipid in the whole manuscript follows the shorthand notation proposed by the Lipidomic Standard Initiative.^[^ ^53^ ^]^ The RBC membrane was composed of abundant lipids, including phosphatidylcholine (PC),^[^ ^54^ ^]^ phosphatidylethanolamine (PE),^[^ ^55^ ^]^ phosphatidyl glycerol (PG), fatty acid (FA),^[^ ^56^ ^]^ sphingomyelin (SM),^[^ ^57^ ^]^ triglycerides (TG),^[^ ^54^ ^]^ and ceramide (Cer).^[^ ^58^ ^]^ The seven deuterium‐labeled lipid standard samples were used as internal standards. Six of them (including FA 16:0[D31], PE 16:0_18:1[D31], PG 16:0_18:1[D31], PC 16:0_18:1[D31], SM 34:1;O2[D31], TG 15:0_15:0_18:1[D5]) were produced by Merck Co., Ltd (Darmstadt, Germany). The Cer 18:0;O2/16:0[D9] was produced by AB SCIEX Co., Ltd (Framingham, MA, USA). The parameters of all lipid isoforms are shown in Table S14 (Supporting Information).

The extraction of plasma lipids was performed as described in our previous study.^[^ ^59^ ^]^ Briefly, a 100 µL plasma sample with ethylenediamine tetraacetic acid (EDTA) was combined with deuterium‐labeled lipid standards and mixed with 300 µL of precooled isopropanol. The mixture was vortexed and centrifuged.

Since previous studies did not have a unified process of RBC membrane lipid extraction protocols, a set of protocols was optimized with five steps. First, a novel RBC tube was applied for RBC separation and precise extraction (Figure S1, Supporting Information). Second, EDTA was used (Figures S2 and S3) among the three common anticoagulants (heparin sodium, sodium citrate, and EDTA). Third, RBC should be subjected to RBC membrane lipid extraction within 3 h ex vivo (Figures S4 and S5, Supporting Information) of the nine ex vivo time points. Fourth, 100 µL RBC was better lysed twice ten times with a volume of 0.2% acetic acid for 10 min each time compared to the three common (Figures S6 and S7, Supporting Information) erythrocyte lysis reagents (pure water,^[^ ^60^ ^]^ 0.2% acetic acid in water,^[^ ^57^ ^]^ and erythrocyte lysate^[^ ^61^ ^]^). Finally, the isopropanol (IPA) was the fastest and most suitable (Figure S8, Supporting Information) for the processing of large batches of samples among the four lipid extraction methods (CHCl_3_/MeOH,^[^ ^62^ ^]^ CHCl_3_/IPA,^[^ ^12^ ^]^ MTBE/MeOH,^[^ ^58^, ^63^ ^]^ and IPA^[^ ^59^ ^]^). Briefly, 100 µL RBCs were extracted using an RBC separator tube after anticoagulation with EDTA. Within 3 h of RBC being isolated, the 100 µL RBC were lysed twice for 10 min each with 1000 µL of 0.2% acetic acid in water. RBC membrane lipids were extracted using precooled isopropanol at 400 µL after centrifugation.

Ultrahigh‐performance liquid chromatography (UHPLC) analysis was performed using the Shimadzu LC‐30AD system (Shimadzu, Kyoto, Japan). Separation was fulfilled by using ACQUITY UPLC BEH C8 column (Waters, 1.7 µm, 2.1 × 100 mm). The mobile phase comprised solution A (water/methanol/ acetonitrile = 3/1/1, *v*/*v*/*v*, 5 mm ammonium acetate) and solution B (isopropanol, 5 mm ammonium acetate). The gradient elution was set as follows: 0−0.5 min, 20% B; 0.5−1.5 min, 20−40% B; 1.5−3 min, 40−60% B; 3−13 min, 60−100% B; 13−14 min, 100% B; 14−17 min, 20% B. The flow rate was 0.3 mL min^−1^, and the injection volume was 2 µL.

Mass spectrometry was performed with a triple quadrupole linear ion trap mass spectrometer (QTRAP 6500+) (AB SCIEX, Framingham, MA, USA) equipped with an ESI source. The general MS parameters were set as follows: gas temperature, 400 °C; ion spray voltage, 5500 V; GS1 and GS2, both 50 psi; and CUR, 35 psi. Furthermore, the mass spectrometry scene with the scheduled pattern contained precursor ions, product ions, and retention time.

### RBC‐Related Indicators and Estimated Plasma Volume Status (ePVS)

Eight RBC‐related indicators were applied, including the RBC count, red blood cell distribution width (RDW), hemoglobin, hematocrit, mean corpuscular volume (MCV), mean corpuscular hemoglobin concentration (MCHC), mean corpuscular hemoglobin (MCH), and erythrocyte stress index (ESI).

The calculation strategy for the ESI was illustrated as follows. ESI was defined as the ratio of RDW to mean corpuscular hemoglobin concentration (MCHC). First, RDW was determined as the standard deviation of erythrocyte volume (EVSD) divided by the mean corpuscular volume (MCV).^[^ ^64^ ^]^ Next, MCHC^[^ ^65^ ^]^ was calculated as the MCH divided by the mean corpuscular volume (MCV). Subsequently, ESI was computed as the ratio of RDW to MCHC. Notably, as MCV was a component in both RDW and MCHC calculations, it effectively cancels out. Thus, ESI was ultimately equivalent to EVSD divided by MCH. ESI can simultaneously characterize the erythrocyte volume standard deviation and the mean hemoglobin levels.

ESI calculated strategy: (1)RDW $=\frac{\mathrm{EVSD}(\mathrm{fL})}{\mathrm{MCV}(\mathrm{fL})}$ × 100%; (2) MCHC(g L^−1^) = $\frac{\mathrm{MCH}(\mathrm{pg})}{\mathrm{MCV}(\mathrm{fL})}$; (3) ESI(mL g^−1^) = $\frac{\mathrm{RDW}}{\mathrm{MCHC}}$; (4)ESI(mL/g) = $\frac{\mathrm{EVSD}(\mathrm{fL})}{\mathrm{MCV}(\mathrm{fL})}$ $\times \frac{\mathrm{MCV}(\mathrm{fL})}{\mathrm{MCH}(\mathrm{pg})}$; (5) ESI(mL g^−1^) = $\frac{\mathrm{EVSD}(\mathrm{fL})}{\mathrm{MCV}(\mathrm{fL})}$ $\times \frac{\mathrm{MCV}(\mathrm{fL})}{\mathrm{MCH}\;(\mathrm{pg})}$; (6) ESI(mL g^−1^) = $\frac{\mathrm{EVSD}(\mathrm{fL})}{\mathrm{MCH}\;(\mathrm{pg})}$.

As the previous studies indicated,^[^ ^66^ ^]^ the ePVS was a verified method to test the plasma volume, and the calculated method of ePVS (mL g^−1^) = $\frac{100-\mathrm{Hematocrit}}{\mathrm{Hemoglobin}\;}$. According to the previous study, the ePVS > 5.5^[^ ^67^ ^]^ was defined as the ePVS overload.

### Statistical Analysis

All lipidomics raw data were analyzed using the Analyst 1.6.2 software (AB SCIEX, Framingham, MA, USA). Peak area integration was performed by Sciex OS1.4 software (AB SCIEX, Framingham, MA, USA). Before statistical analysis, data filtration, sum normalization, and deuterium‐labeled lipid standards adjustment were performed in R software (version 4.3.1).

Analytical procedures were performed in R (version 4.3.1). Variables with missing data exceeding 20% of the population were excluded from the analysis. Continuous variables were summarized as interquartile ranges. Categorical data (e.g., sex and CHF) were applied for the chi‐square test.

PCA analysis was performed with “prcomp” function of “ropls” package (version 1.38.0) in R. The R package “limma” (version 3.62.1) was performed to explore differential lipids with Benjamini‐Hochberg methods and three functions, “lmFit”, “makeContrasts”, and “eBayes”. The lipids were determined to be statistically significant with the absolute value of log2 fold change >1 and adjusted P‐value < 0.05.

Numerous variables can influence the lipid composition of the RBC membrane, such as age, sex, and underlying diseases. For example, women may have higher rates of anemia^[^ ^68^ ^]^ than men, and older individuals may be more prone to anemia than younger individuals.^[^ ^69^ ^]^ Multivariate logistic and Cox proportional hazards models were widely employed in clinical research to obtain a more accurate analysis of the relationship between CHF and RBC membrane lipids. Cox proportional hazards model, logistic regression, were computed with confidence intervals (CIs), odds ratios (OR), and hazard ratios (HR). Four models with adjusted parameters were established, with confounding variables included in the model adjustment. The receiver operating characteristic curve (ROC) and Kaplan‐Meier (KM) survival analysis were applied to verify the diagnosis or predictive value.

All the initial codes for R software were shared (https://github.com/Linzhang‐BiuBiuBiu/RBC‐membrane‐lipid)

### Ethics Approval Statement

In TJHFIT, the First Teaching Hospital of Tianjin University of TCM approved the ethics with ID: TYLL2023[K]. JHDH ethics review committee approved the research with ID: JHYYLL‐2022‐0307.

## Conflict of Interest

The authors declare no conflict of interest.

## Author Contributions

XG, LH, and GF design and put forward the conceptualization. LZ and XO organized the data and finished the original draft. JL and XL finished writing to review. XG, GF provide the funding acquisition. JZ promotes resources and validation. XZ, PZ, LL, and ZZ contributed to the investigation and methodology. Access and validation of the underlying data LZ and XO. XG and GF give the funding support.

## Supporting information



Supporting Information

## Data Availability

All data for this study can be obtained by contacting the corresponding author.
