# Supplementary material for: Red Blood Cell Membrane Lipidomics: Potential Biomarkers Detecting Method for Plasma Volume Overload and Major Adverse Cardiovascular Events in Chronic Heart Failure Patients
Source: Adv Sci (Weinh). 2025 Jun 23;12(33):e02893. doi: 10.1002/advs.202502893 (PMC12412543; doi:10.1002/advs.202502893)
Supplement: Supplementary file 1 — Supporting Information [file ADVS-12-e02893-s001.docx]

Supporting Information

Red Blood Cell Membrane Lipidomics: Potential Biomarkers Detecting Method for Plasma Volume Overload and Major Adverse Cardiovascular Events in Chronic Heart Failure Patients

*Lin Zhang, Xiangqin Ou, Jingyi Lin, Xiaofei Luo, Jiashun Zhou, Xingyue Zhou, Peihua Zhao, Li Liu, Ziran Zhao, Ying Zhou, Guanwei Fan, Lifeng Han, Xiumei Gao*


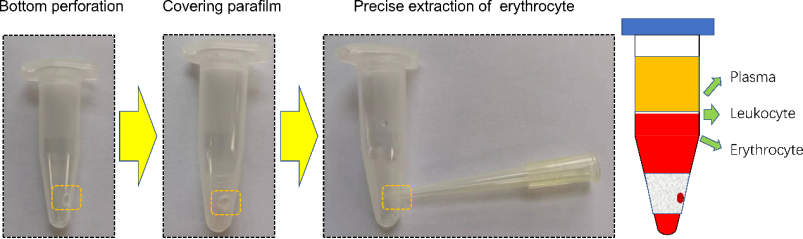


**Figure. S1. The novel RBC tube for precise extraction of the RBC** (Commercial 1.5 mL Eppendorf tube was proposed for the precise extraction of RBC. A 2 mm wire was heated and perforated the side walls of the 1.5 mL Eppendorf tubes. The small holes were generated and covered with five layers of parafilm to make the tubes suitable for the collection of RBC. After centrifugation of whole blood at a low speed of 300 *g*, RBC can be extracted from the bottom.)


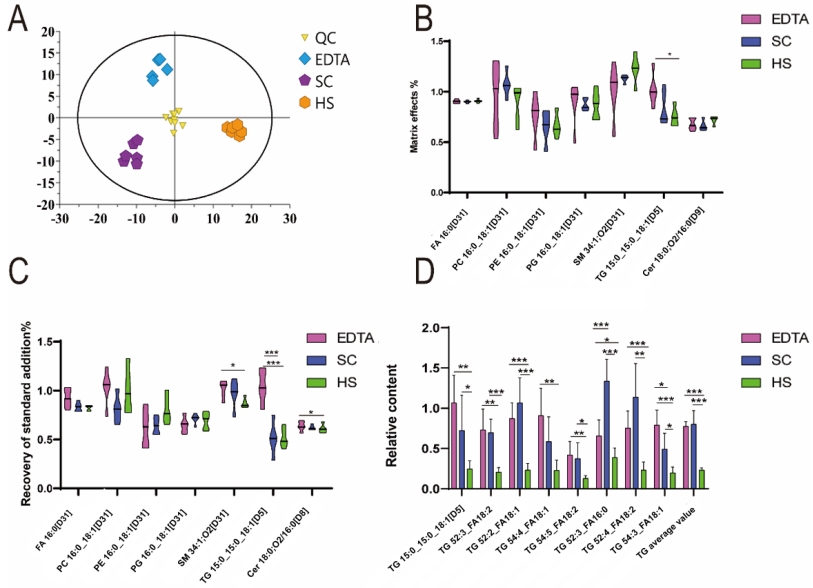
**Figure. S2. Comparison of three anticoagulants.** A). The PCA analysis of anticoagulants solvent B). The seven lipid standards showed the matrix effects C) and recovery of standard addition D). The difference of various TGs among the three anticoagulant solvents. (EDTA, ethylenediaminetetraacetic acid; SC, sodium citrate; HS, heparin sodium; * mean *P* < 0.05, ** mean *P* < 0.01, *** mean *P* < 0.001.)


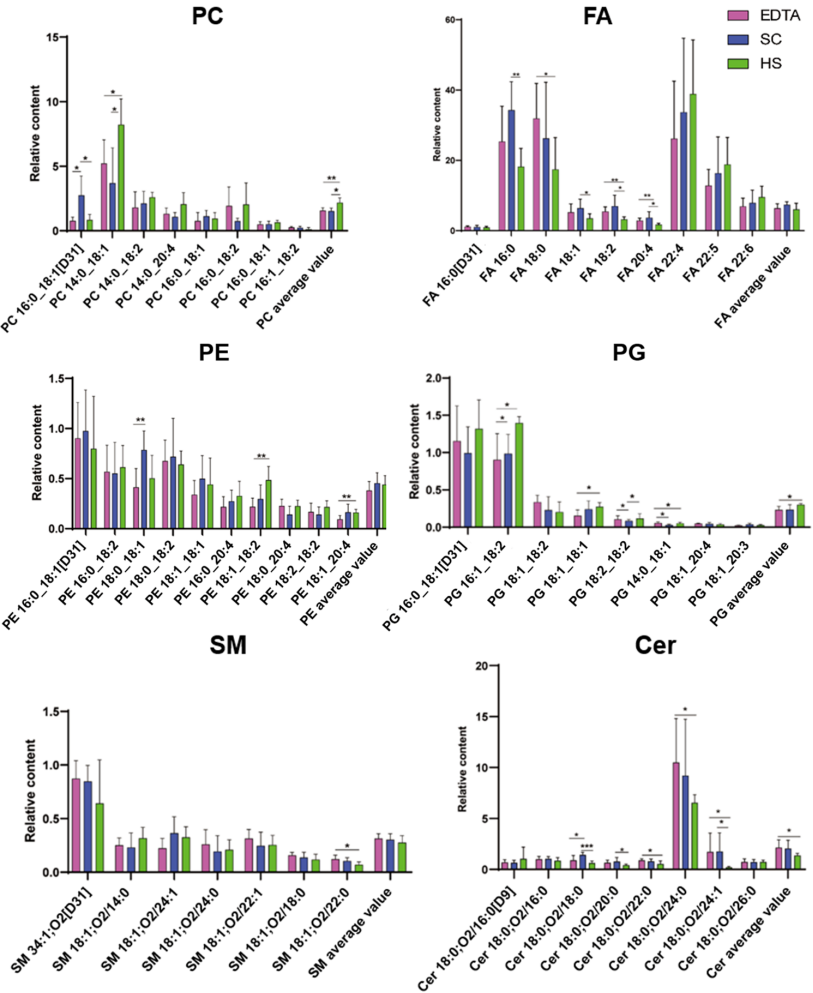


**Figure. S3. The comparison of RBC membrane six lipids subtypes in anticoagulants** (* mean *P* < 0.05, ** mean *P* < 0.01, *** mean *P* < 0.001)


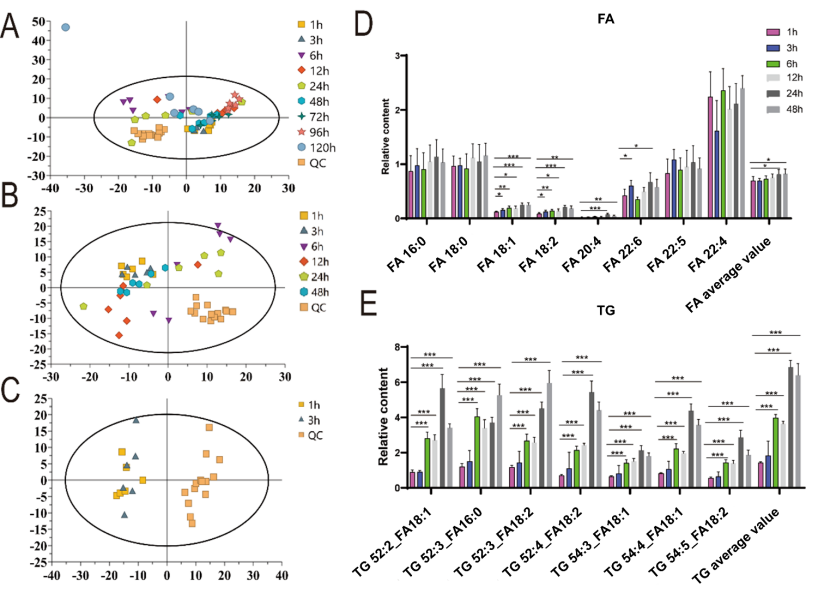


**Figure. S4. Effect of time on RBC membrane lipids.** A). PCA analysis of 9-time points B). PCA analysis of 6-time points and 3 points were excluded because of outliers C). PCA analysis among 1 hour, 3 hours, and QC D). The FA comparison analysis of relative content. (E) The TG comparison analysis of relative content. * mean *P* < 0.05, ** mean *P* < 0.01, *** mean *P* < 0.001.


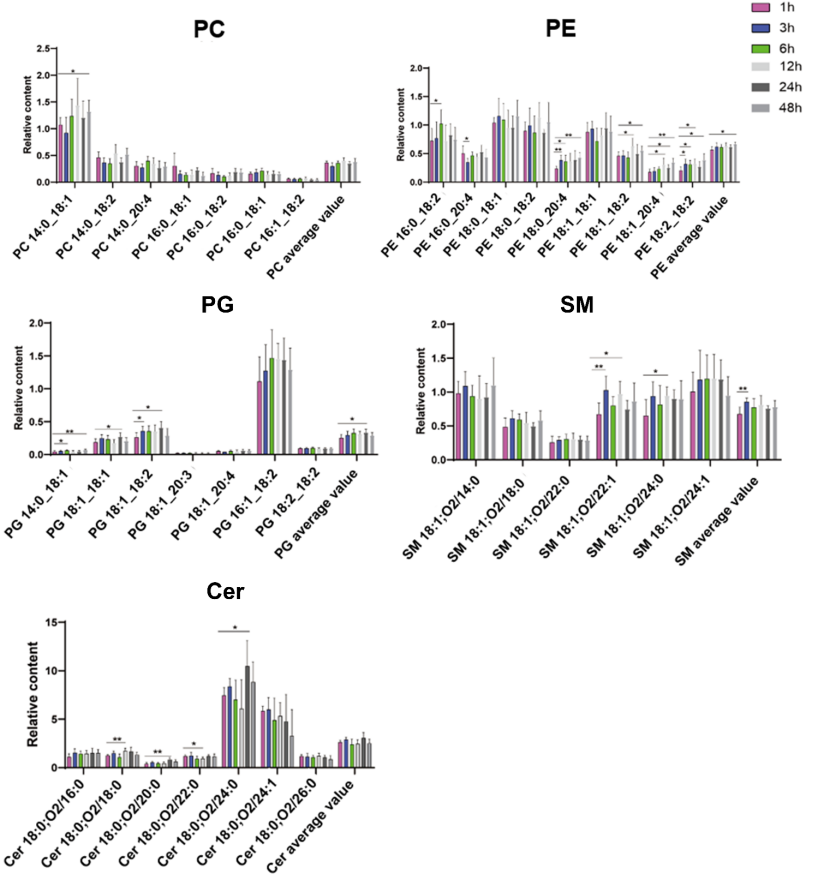


**Figure S5. The comparison of various handling times among the other five types of lipids** (* mean *P* < 0.05, ** mean *P* < 0.01, *** mean *P* < 0.001)


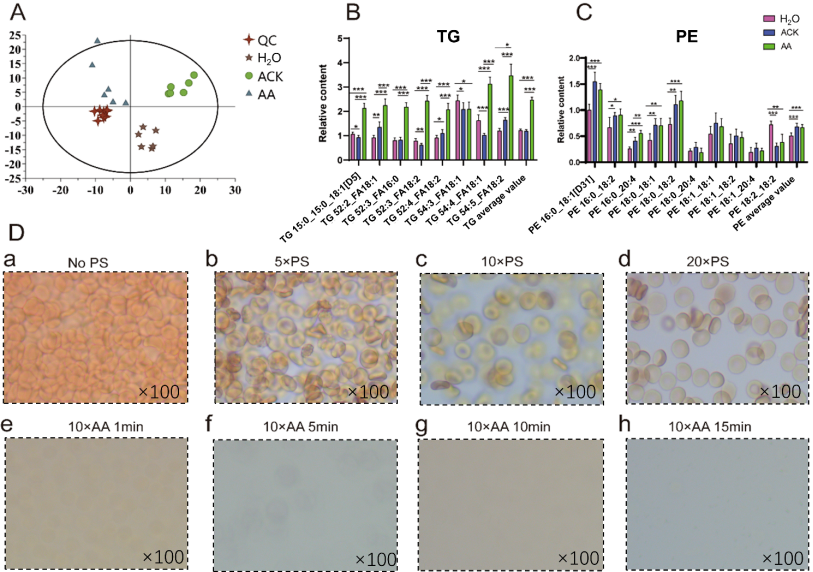


**Figure. S6. Analysis of three erythrocyte lysates.** A). PCA analysis of three erythrocyte lysates B). The TG comparison analysis of relative content C). The PE comparison analysis D a-d). Erythrocytes after adding zero, five, ten, and twenty PS solution D e-h). Erythrocytes after lysis with ten times AA solutions for one, five, ten, and fifteen minutes. (H_2_O, pure water. ACK, erythrocyte lysate. AA, 0.2% acetic acid water. PS, physiological saline. * mean *P* < 0.05, ** mean *P* < 0.01, *** mean *P* < 0.001.)


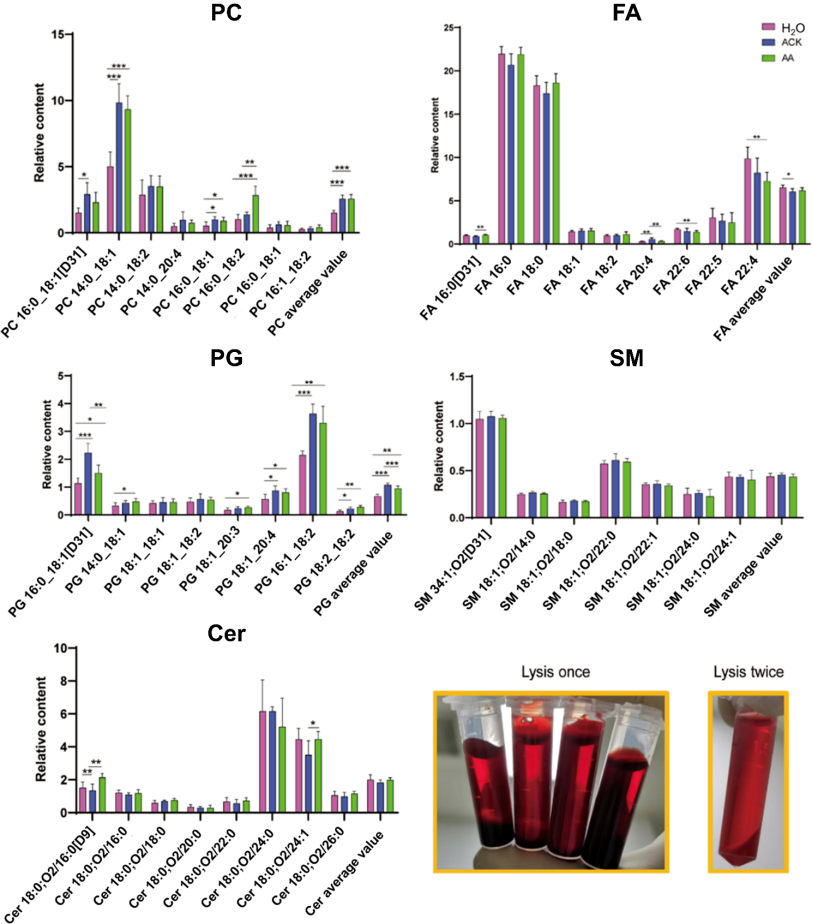


**Figure S7. The comparison of other five lipid subtypes in various erythrocyte lysates** (* mean *P* < 0.05, ** mean *P* < 0.01, *** mean *P* < 0.001)


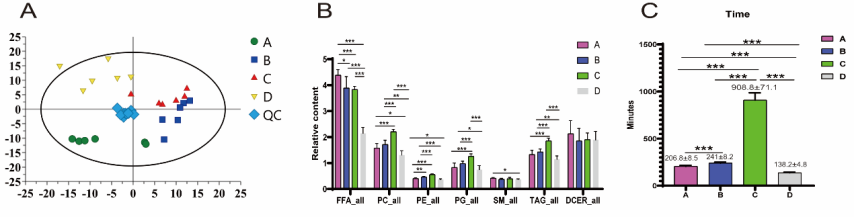


**Figure. S8. Analysis of four lipid extraction methods.** A). PCA analysis of four lipid extraction methods B). Seven lipid subtypes of the four lipid extraction methods C). Sample processing time for the four extraction methods. (A, CHCl_3_/MeOH 2:1 *v*/*v*. B, CHCl_3_/IPA 3:5 *v*/*v*. C, MeOH/ MTBE 3:10 *v*/*v*. D, IPA. * mean *P* < 0.05, ** mean *P* < 0.01, *** mean *P* < 0.001.)

**Table S1. The baseline character in Con vs CHF patients of TJHFIT**

| Characteristic | Overall | CHF | Con | *P* value |
| --- | --- | --- | --- | --- |
|  | N = 507,638 | N = 79,624 | N = 428,014 |  |
| Hospital death, n (%) | 11,422 (2.3%) | 4,002 (5.0%) | 7,420 (1.7%) | < 0.001 |
| LOS, days | 3.0 (1.0, 6.0) | 5.0 (2.0, 9.0) | 3.0 (1.0, 5.0) | < 0.001 |
| Medical history |  |  |  |  |
| DM, n(%) | 133,847 (26%) | 38,339 (48%) | 95,508 (22%) | < 0.001 |
| CVD, n(%) | 107,173 (21%) | 43,059 (54%) | 64,114 (15%) | < 0.001 |
| Hypertension, n(%) | 200,604 (40%) | 25,737 (32%) | 174,867 (41%) | < 0.001 |
| Hyperlipidemia, n(%) | 171,072 (34%) | 43,780 (55%) | 127,292 (30%) | < 0.001 |
| CKD, n(%) | 142,182 (28%) | 49,526 (62%) | 92,656 (22%) | < 0.001 |
| AF, n(%) | 76,429 (15%) | 36,578 (46%) | 39,851 (9.3%) | < 0.001 |
| Nicotine dependance, n(%) | 146,060 (29%) | 30,290 (38%) | 115,770 (27%) | < 0.001 |
| Anemia, n(%) | 126,079 (25%) | 33,421 (42%) | 92,658 (22%) | < 0.001 |
| Hyperuricemia, n(%) | 24,657 (4.9%) | 10,416 (13%) | 14,241 (3.3%) | < 0.001 |
| Obstructive sleep apnea, n(%) | 38,596 (7.6%) | 12,956 (16%) | 25,640 (6.0%) | < 0.001 |
| Demographic |  |  |  |  |
| Sex, male, n(%) | 242,512 (48%) | 42,180 (53%) | 200,332 (47%) | < 0.001 |
| Age, year | 59 (44, 72) | 71 (61, 81) | 56 (41, 69) | < 0.001 |
| RBC indicators |  |  |  |  |
| ESI, cL g^-1^ | 4.22 (3.90, 4.76) | 4.70 (4.26, 5.32) | 4.15 (3.86, 4.63) | < 0.001 |
| RDW, % | 14.00 (13.10, 15.40) | 15.10 (14.00, 16.80) | 13.80 (13.00, 15.10) | < 0.001 |
| RBC count, m uL^-1^ | 4.10 (3.58, 4.55) | 3.81 (3.29, 4.32) | 4.14 (3.65, 4.58) | < 0.001 |
| Hematocrit, % | 37 (33, 41) | 35 (30, 39) | 37 (33, 41) | < 0.001 |
| Hemoglobin, g dL^-1^ | 12.10 (10.60, 13.50) | 11.10 (9.60, 12.60) | 12.30 (10.80, 13.70) | < 0.001 |
| MCH, pg | 30.00 (28.30, 31.40) | 29.60 (27.70, 31.20) | 30.00 (28.50, 31.40) | < 0.001 |
| MCHC, g dL^-1^ | 33.00 (32.00, 34.10) | 32.20 (31.10, 33.20) | 33.20 (32.10, 34.20) | < 0.001 |
| MCV, fL | 90 (86, 94) | 91 (87, 96) | 90 (86, 94) | < 0.001 |
| Other laboratory tests |  |  |  |  |
| Platelet, K uL^-1^ | 231 (179, 293) | 215 (165, 278) | 234 (182, 295) | < 0.001 |
| Potassium,mEq L^-1^ | 4.20 (3.80, 4.60) | 4.40 (3.90, 4.90) | 4.10 (3.80, 4.50) | < 0.001 |
| Sodium, mEq L^-1^ | 139.0 (136.0, 141.0) | 138.0 (135.0, 141.0) | 139.0 (136.0, 141.0) | < 0.001 |
| AnionGap, mEq L^-1^ | 14.0 (12.0, 17.0) | 15.0 (13.0, 17.0) | 14.0 (12.0, 17.0) | < 0.001 |
| BUN, mg dL^-1^ | 16 (12, 24) | 27 (18, 43) | 15 (11, 21) | < 0.001 |
| WBC, K uL^-1^ | 8.4 (6.3, 11.3) | 8.4 (6.4, 11.2) | 8.4 (6.3, 11.3) | 0.8 |
| Bicarbonate, mEq L^-1^ | 25.0 (22.0, 27.0) | 25.0 (22.0, 28.0) | 25.0 (22.0, 27.0) | < 0.001 |
| Creatinine, mg dL^-1^ | 0.90 (0.70, 1.20) | 1.30 (1.00, 2.00) | 0.90 (0.70, 1.10) | < 0.001 |
| Glucose, mg dL^-1^ | 111 (96, 140) | 123 (101, 165) | 110 (95, 136) | < 0.001 |

**#Continuous variables are expressed as interquartile ranges. Categorical variables are expressed as frequency (percentage). LOS, length of stay, DM, diabetes mellitus, CVD, cardiovascular diseases, CKD, chronic kidney disease, AF, atrial fibrillation, ESI, erythrocyte stress index, RDW, red blood cell distribution width, MCH, mean corpuscular hemoglobin, MCHC, mean corpuscular hemoglobin concentration, MCV, mean cell volume, BUN, blood urea nitrogen, WBC, white blood cell count.**

**Table S2. The baseline character in the CHF hospital statue**

| Characteristic | Overall | In-hospital survive | In-hospital death | *P* value |
| --- | --- | --- | --- | --- |
|  | N = 79,624 | N = 75,622 | N = 4,002 |  |
| LOS, days | 5 (2, 9) | 5 (2, 8) | 7 (3, 15) | < 0.001 |
| Medical history |  |  |  |  |
| DM, n(%) | 38,339 (48%) | 36,577 (48%) | 1,762 (44%) | < 0.001 |
| CVD, n(%) | 43,059 (54%) | 40,734 (54%) | 2,325 (58%) | < 0.001 |
| Hypertension, n(%) | 25,737 (32%) | 24,424 (32%) | 1,313 (33%) | 0.5 |
| Hyperlipidemia, n(%) | 43,780 (55%) | 41,873 (55%) | 1,907 (48%) | < 0.001 |
| CKD, n(%) | 49,526 (62%) | 46,238 (61%) | 3,288 (82%) | < 0.001 |
| AF, n(%) | 36,578 (46%) | 34,310 (45%) | 2,268 (57%) | < 0.001 |
| Nicotine dependance, n(%) | 30,290 (38%) | 28,752 (38%) | 1,538 (38%) | 0.6 |
| Anemia, n(%) | 33,421 (42%) | 31,453 (42%) | 1,968 (49%) | < 0.001 |
| Hyperuricemia, n(%) | 10,416 (13%) | 9,959 (13%) | 457 (11%) | 0.001 |
| Obstructive sleep apnea, n(%) | 12,956 (16%) | 12,524 (17%) | 432 (11%) | < 0.001 |
| Demographic |  |  |  |  |
| Sex, male, n(%) | 42,180 (53%) | 40,002 (53%) | 2,178 (54%) | 0.06 |
| Age, year | 71 (61, 81) | 71 (61, 81) | 76 (66, 84) | < 0.001 |
| RBC indicators |  |  |  |  |
| ESI, cL g^-1^ | 4.70 (4.26, 5.32) | 4.68 (4.25, 5.30) | 5.05 (4.52, 5.77) | < 0.001 |
| RDW, % | 15.10 (14.00, 16.80) | 15.10 (14.00, 16.70) | 16.00 (14.60, 17.90) | < 0.001 |
| RBC, m uL^-1^ | 3.81 (3.29, 4.32) | 3.82 (3.30, 4.33) | 3.62 (3.07, 4.18) | < 0.001 |
| Hematocrit, % | 35 (30, 39) | 35 (30, 39) | 34 (29, 38) | < 0.001 |
| Hemoglobin, g dL^-1^ | 11.10 (9.60, 12.60) | 11.10 (9.60, 12.70) | 10.60 (9.00, 12.20) | < 0.001 |
| MCH, pg | 29.60 (27.70, 31.20) | 29.60 (27.70, 31.20) | 29.80 (27.90, 31.40) | < 0.001 |
| MCHC, g dL^-1^ | 32.20 (31.10, 33.20) | 32.20 (31.10, 33.30) | 31.80 (30.70, 32.90) | < 0.001 |
| MCV, fL | 91 (87, 96) | 91 (87, 96) | 93 (88, 98) | < 0.001 |
| Other laboratory tests |  |  |  |  |
| Platelet, K uL^-1^ | 215 (165, 278) | 215 (166, 278) | 202 (142, 278) | < 0.001 |
| Potassium,mEq L^-1^ | 4.40 (3.90, 4.90) | 4.30 (3.90, 4.80) | 4.50 (4.00, 5.20) | < 0.001 |
| Sodium, mEq L^-1^ | 138.0 (135.0, 141.0) | 138.0 (136.0, 141.0) | 138.0 (134.0, 141.0) | < 0.001 |
| AnionGap, mEq L^-1^ | 15.0 (13.0, 17.0) | 15.0 (13.0, 17.0) | 16.0 (14.0, 20.0) | < 0.001 |
| BUN, mg dL^-1^ | 27 (18, 43) | 27 (18, 42) | 36 (23, 57) | < 0.001 |
| WBC, K uL^-1^ | 8.4 (6.4, 11.2) | 8.3 (6.4, 11.0) | 10.8 (7.4, 15.6) | < 0.001 |
| Bicarbonate, mEq L^-1^ | 25.0 (22.0, 28.0) | 25.0 (22.0, 28.0) | 23.0 (19.0, 27.0) | < 0.001 |
| Creatinine, mg dL^-1^ | 1.30 (1.00, 2.00) | 1.30 (1.00, 2.00) | 1.60 (1.10, 2.60) | < 0.001 |
| Glucose, mg dL^-1^ | 123 (101, 165) | 122 (101, 164) | 135 (106, 183) | < 0.001 |

**#Continuous variables are expressed as interquartile ranges. Categorical variables are expressed as frequency (percentage). LOS, length of stay, DM, diabetes mellitus, CVD, cardiovascular diseases, CKD, chronic kidney disease, AF, atrial fibrillation, ESI, erythrocyte stress index, RDW, red blood cell distribution width, MCH, mean corpuscular hemoglobin, MCHC, mean corpuscular hemoglobin concentration, MCV, mean cell volume, BUN, blood urea nitrogen, WBC, white blood cell count.**

**Table S3. The logistic regression of CHF prevalence in TJHFIT.**

| Characteristic | Model 1 | | Model 2 | | Model 3 | | Model 4 | |
| --- | --- | --- | --- | --- | --- | --- | --- | --- |
|  | OR (95% CI) | *P* value | OR (95% CI) | *P* value | OR (95% CI) | *P* value | OR (95% CI) | *P* value |
| ESI | 1.96 (1.94, 1.98) | <0.001 | 1.92 (1.90, 1.93) | <0.001 | 1.55 (1.53, 1.57) | <0.001 | 1.57 (1.55, 1.59) | <0.001 |
| MCV | 1.03 (1.03, 1.03) | <0.001 | 1.01 (1.00, 1.01) | <0.001 | 1.00 (1.00, 1.00) | 0.9 | 1.00 (1.00, 1.00) | 0.13 |
| RDW | 1.25 (1.25, 1.25) | <0.001 | 1.24 (1.24, 1.24) | <0.001 | 1.16 (1.15, 1.16) | <0.001 | 1.16 (1.16, 1.17) | <0.001 |
| MCHC | 0.70 (0.69, 0.70) | <0.001 | 0.71 (0.71, 0.71) | <0.001 | 0.82 (0.81, 0.82) | <0.001 | 0.81 (0.81, 0.82) | <0.001 |
| MCH | 0.95 (0.94, 0.95) | <0.001 | 0.91 (0.91, 0.92) | <0.001 | 0.94 (0.94, 0.95) | <0.001 | 0.94 (0.94, 0.94) | <0.001 |
| RBC | 0.60 (0.60, 0.61) | <0.001 | 0.71 (0.70, 0.72) | <0.001 | 0.95 (0.94, 0.96) | <0.001 | 0.93 (0.91, 0.94) | <0.001 |
| Hemoglobin | 0.81 (0.81, 0.81) | <0.001 | 0.84 (0.84, 0.84) | <0.001 | 0.94 (0.93, 0.94) | <0.001 | 0.93 (0.92, 0.93) | <0.001 |
| Hematocrit | 0.95 (0.95, 0.95) | <0.001 | 0.96 (0.96, 0.96) | <0.001 | 0.99 (0.99, 0.99) | <0.001 | 0.99 (0.99, 0.99) | <0.001 |

**#ESI, erythrocyte stress index, MCV, mean corpuscular volume, RDW, red blood cell distribution width, MCHC, mean corpuscular hemoglobin concentration, MCH, mean corpuscular hemoglobin, RBC, and red blood cell count. The adjusted details were the following, Model 1: non-adjustment, Model 2: age + sex; Model 3: LOS + DM + CVD + Hypertension + Hyperlipidemia + CKD + AF + Nicotine dependence + Anemia + Hyperuricemia + Obstructive sleep apnea, Model 4: Model 3 + Platelet + Glucose + Potassium + WBC + Bicarbonate + Creatinine + Bicarbonate.**

**Table S4. The logistic regression of CHF hospital death in TJHFIT.**

| Characteristic | Model 1 | | Model 2 | | Model 3 | | Model 4 | |
| --- | --- | --- | --- | --- | --- | --- | --- | --- |
|  | OR (95% CI) | *P* value | OR (95% CI) | *P* value | OR (95% CI) | *P* value | OR (95% CI) | *P* value |
| ESI | 1.46 (1.41, 1.50) | <0.001 | 1.50 (1.45, 1.54) | <0.001 | 1.40 (1.36, 1.45) | <0.001 | 1.41 (1.37, 1.46) | <0.001 |
| MCV | 1.03 (1.03, 1.04) | <0.001 | 1.03 (1.02, 1.03) | <0.001 | 1.02 (1.02, 1.03) | <0.001 | 1.02 (1.01, 1.02) | <0.001 |
| RDW | 1.16 (1.14, 1.17) | <0.001 | 1.17 (1.15, 1.18) | <0.001 | 1.13 (1.12, 1.15) | <0.001 | 1.14 (1.12, 1.15) | <0.001 |
| MCHC | 0.86 (0.85, 0.88) | <0.001 | 0.85 (0.83, 0.87) | <0.001 | 0.88 (0.86, 0.90) | <0.001 | 0.88 (0.86, 0.90) | <0.001 |
| MCH | 1.03 (1.01, 1.04) | <0.001 | 1.01 (1.00, 1.02) | 0.023 | 1.01 (1.00, 1.03) | 0.014 | 1.01 (1.00, 1.03) | 0.014 |
| RBC | 0.74 (0.71, 0.78) | <0.001 | 0.76 (0.73, 0.80) | <0.001 | 0.87 (0.83, 0.91) | <0.001 | 0.89 (0.85, 0.93) | <0.001 |
| Hemoglobin | 0.91 (0.90, 0.92) | <0.001 | 0.91 (0.90, 0.92) | <0.001 | 0.95 (0.94, 0.97) | <0.001 | 0.96 (0.94, 0.97) | <0.001 |
| Hematocrit | 0.98 (0.97, 0.98) | <0.001 | 0.98 (0.97, 0.98) | <0.001 | 1.0 (0.99, 1.00) | 0.055 | 1.0 (0.99, 1.00) | 0.053 |

**#ESI, erythrocyte stress index, MCV, mean corpuscular volume, RDW, red blood cell distribution width, MCHC, mean corpuscular hemoglobin concentration, MCH, mean corpuscular hemoglobin, RBC, red blood cell count. The adjusted details were following, Model 1: non adjustment, Model 2: age + sex; Model 3: LOS + DM + CVD + Hypertension + Hyperlipidemia + CKD + AF + Nicotine dependence + Anemia + Hyperuricemia + Obstructive sleep apnea, Model 4: Model 3 + Platelet + Glucose + Potassium + WBC + Bicarbonate + Creatinine + Bicarbonate.**

**Table S5. The baseline characteristics of Con vs CHF individuals in JHDH.**

| Characteristic | Overall | Con | CHF | *P* value |
| --- | --- | --- | --- | --- |
|  | N = 1,550 | N = 866 | N = 684 |  |
| Demographic |  |  |  |  |
| Age, year | 65.00 (54.00, 74.00) | 61.00 (49.00, 70.00) | 70.00 (59.00, 77.50) | <0.001 |
| Sex, male, n(%) | 817 (53%) | 434 (50%) | 383 (56%) | 0.021 |
| Medical history |  |  |  |  |
| CVD, n(%) | 1,260 (81%) | 591 (68%) | 669 (98%) | <0.001 |
| AF, n(%) | 197 (13%) | 54 (6.2%) | 143 (21%) | <0.001 |
| Stroke, n(%) | 181 (12%) | 89 (10%) | 92 (13%) | 0.053 |
| DM, n(%) | 383 (25%) | 162 (19%) | 221 (32%) | <0.001 |
| Hyperlipidemia, n(%) | 270 (17%) | 123 (14%) | 147 (21%) | <0.001 |
| Hyperuricemia, n(%) | 71 (4.6%) | 19 (2.2%) | 52 (7.6%) | <0.001 |
| CKD, n(%) | 321 (21%) | 124 (14%) | 197 (29%) | <0.001 |
| Hypertension, n(%) | 892 (58%) | 433 (50%) | 459 (67%) | <0.001 |
| COPD, n(%) | 72 (4.6%) | 24 (2.8%) | 48 (7.0%) | <0.001 |
| Anemia, n(%) | 194 (13%) | 81 (9.4%) | 113 (17%) | <0.001 |
| Life style |  |  |  |  |
| Smoking history, n(%) | 216 (14%) | 100 (12%) | 116 (17%) | 0.002 |
| Alcohol history, n(%) | 247 (16%) | 109 (13%) | 138 (20%) | <0.001 |
| Laboratory tests |  |  |  |  |
| BNP, ng L^-1^ | 62.58 (11.89, 302.00) | 18.00 (10.00, 48.46) | 334.26 (232.66, 659.93) | <0.001 |
| ESI, cL g^-1^ | 3.87 (3.63, 4.18) | 3.77 (3.57, 4.05) | 4.02 (3.74, 4.36) | <0.001 |
| ePVS, mL g^-1^ | 4.44 (3.77, 5.53) | 4.34 (3.72, 5.11) | 4.63 (3.84, 6.15) | <0.001 |
| RBC count, m uL^-1^ | 4.46 (3.98, 4.90) | 4.50 (4.06, 4.90) | 4.40 (3.86, 4.89) | <0.001 |
| Hematocrit, % | 40.60 (36.80, 44.30) | 40.85 (37.50, 44.50) | 40.30 (35.55, 44.00) | 0.002 |
| RDW, % | 13.00 (12.40, 13.80) | 12.70 (12.20, 13.40) | 13.40 (12.70, 14.40) | <0.001 |
| Hemoglobin, g dL^-1^ | 137.00 (122.00, 151.00) | 138.00 (126.00, 152.00) | 135.00 (118.00, 151.00) | <0.001 |
| MCV, fL | 91.60 (88.40, 95.20) | 91.50 (88.30, 94.90) | 92.00 (88.40, 95.30) | 0.3 |
| MCH, pg | 30.80 (29.70, 32.00) | 30.80 (29.80, 32.00) | 30.90 (29.60, 32.10) | 0.7 |
| MCHC, g dL^-1^ | 336.00 (327.00, 344.00) | 337.00 (329.00, 344.00) | 334.00 (325.00, 343.00) | <0.001 |
| Globulin, g L^-1^ | 24.20 (21.20, 27.00) | 23.80 (20.50, 26.80) | 24.65 (21.70, 27.75) | <0.001 |
| Glucose, mmol L^-1^ | 5.97 (5.14, 7.32) | 5.70 (5.06, 6.81) | 6.49 (5.30, 8.10) | <0.001 |
| Basophils count, K μL^-1^ | 0.02 (0.01, 0.03) | 0.02 (0.01, 0.03) | 0.02 (0.01, 0.03) | <0.001 |
| Albumin, g L^-1^ | 42.70 (39.10, 47.60) | 44.80 (40.30, 49.70) | 40.80 (37.60, 44.20) | <0.001 |
| Total triglycerides, mmol L^-1^ | 1.26 (0.89, 1.93) | 1.38 (0.96, 2.21) | 1.16 (0.82, 1.67) | <0.001 |
| TC, mmol L^-1^ | 4.34 (3.56, 5.19) | 4.27 (3.57, 5.11) | 4.44 (3.55, 5.26) | 0.2 |
| LDL, mmol L^-1^ | 2.54 (1.92, 3.27) | 2.47 (1.91, 3.11) | 2.71 (1.96, 3.44) | <0.001 |
| HDL, mmol L^-1^ | 1.10 (0.91, 1.33) | 1.08 (0.90, 1.32) | 1.12 (0.93, 1.33) | 0.12 |
| ALT, U L^-1^ | 18.00 (12.00, 33.00) | 17.00 (11.00, 30.00) | 20.00 (13.00, 35.00) | <0.001 |
| AST, U L^-1^ | 21.00 (16.00, 39.00) | 19.00 (15.00, 28.00) | 25.00 (17.00, 65.00) | <0.001 |
| ALP, U L^-1^ | 76.00 (60.00, 94.00) | 72.00 (57.00, 88.00) | 81.00 (64.00, 100.00) | <0.001 |
| Lym, K μL^-1^ | 1.61 (1.13, 2.23) | 1.72 (1.24, 2.24) | 1.47 (1.01, 2.22) | <0.001 |
| MPV, fL | 9.90 (9.30, 10.70) | 9.90 (9.20, 10.60) | 9.90 (9.30, 10.80) | 0.018 |
| Platelets count, K μL^-1^ | 226.00 (182.00, 270.00) | 230.50 (189.00, 272.00) | 219.00 (172.00, 266.00) | 0.002 |
| PDW, % | 11.30 (10.10, 12.80) | 11.20 (9.90, 12.60) | 11.55 (10.20, 13.20) | <0.001 |
| DBIL, μmol L^-1^ | 4.50 (3.30, 6.00) | 4.20 (3.20, 5.60) | 4.80 (3.50, 6.80) | <0.001 |
| WBC, K μL^-1^ | 6.99 (5.55, 9.03) | 6.57 (5.31, 8.09) | 7.79 (5.99, 10.29) | <0.001 |
| IDBL, μmol L^-1^ | 5.90 (3.60, 8.80) | 5.90 (3.60, 8.60) | 5.90 (3.60, 9.35) | 0.3 |
| TBI, μmol L^-1^ | 10.50 (7.20, 14.80) | 10.20 (7.10, 14.00) | 11.00 (7.30, 16.25) | 0.002 |
| TBA, μmol L^-1^ | 3.10 (1.80, 5.30) | 3.30 (2.00, 5.90) | 2.85 (1.70, 4.80) | <0.001 |
| Medical therapy |  |  |  |  |
| ACEI/ARB, n(%) | 603 (39%) | 296 (34%) | 307 (45%) | <0.001 |
| Beta-blockers, n(%) | 573 (37%) | 276 (32%) | 297 (43%) | <0.001 |
| CCB, n(%) | 588 (38%) | 277 (32%) | 311 (45%) | <0.001 |
| Metformin, n(%) | 247 (16%) | 119 (14%) | 128 (19%) | 0.008 |
| Insulin injections, n(%) | 185 (12%) | 91 (11%) | 94 (14%) | 0.051 |
| Statins, n(%) | 201 (13%) | 93 (11%) | 108 (16%) | 0.003 |
| Diuretics, n(%) | 169 (11%) | 68 (7.9%) | 101 (15%) | <0.001 |

**#Continuous variables are expressed as interquartile ranges. Categorical variables are expressed as frequency (percentage). CVD, cardiovascular disease, AF, atrial fibrillation, CKD, chronic kidney disease, COPD, Chronic obstructive pulmonary disease, BNP, B-type natriuretic peptide, RBC, red blood cell count, RDW, red blood cell distribution, MCV, mean cell volume, MCH, mean corpuscular hemoglobin, MCHC, mean corpuscular hemoglobin concentration, TC, total cholesterol, LDL, low density lipoprotein, HDL, high density lipoprotein, ALT, alanine aminotransferase, AST, aspartate aminotransferase, ALP, alkaline phosphatase, Lym, lymphocyte count, MPV, mean platelet volume, PDW, platelet distribution width, DBIL, direct bilirubin, WBC, white blood cell count, IBIL, indirect bilirubin, TBI, total bilirubin, TBA, total bile acids, ACEI/ARB, ACE-inhibitors/AT1-receptor antagonists, CCB, dihydropyridine Ca2+ channel blockers.**

**Table S6. The RBC membrane lipidomics difference between the CHF and Con**

| Lipid | log2FC | adj.P.Val | FC | lnFC | t | B |
| --- | --- | --- | --- | --- | --- | --- |
| Cer 18:0;O2/16:0 | 1.27 | 1.31957E-06 | 2.4 | 0.88 | 5.38 | 7.31 |
| GlcCer 18:0;O2/18:0 | -1.14 | 9.62607E-06 | 0.45 | -0.79 | -4.9 | 4.89 |
| GlcCer 18:1;O2/14:0 | 1.45 | 3.69024E-06 | 2.74 | 1.01 | 5.12 | 5.94 |
| GlcCer 18:1;O2/16:0 | 1.03 | 0.000242662 | 2.05 | 0.72 | 4.04 | 1.12 |
| LPE 16:0 | 2.59 | 2.27606E-07 | 6 | 1.79 | 5.86 | 9.91 |
| LPE 16:1 | 2.97 | 1.25451E-05 | 7.86 | 2.06 | 4.82 | 4.51 |
| LPE 18:0 | 1.75 | 4.45538E-07 | 3.36 | 1.21 | 5.67 | 8.87 |
| LPE 18:1 | 2.65 | 2.51098E-06 | 6.3 | 1.84 | 5.2 | 6.37 |
| LPE 20:0 | 2.52 | 2.16293E-07 | 5.74 | 1.75 | 5.9 | 10.13 |
| LPE 20:1 | 2.79 | 2.16293E-07 | 6.93 | 1.94 | 5.93 | 10.3 |
| LPE 20:2 | 1.54 | 0.000677797 | 2.91 | 1.07 | 3.74 | -0.01 |
| PE 18:1_22:4 | -1.2 | 2.59843E-05 | 0.43 | -0.83 | -4.63 | 3.62 |
| PE 18:2_18:2 | -1.4 | 2.31344E-06 | 0.38 | -0.97 | -5.24 | 6.57 |
| PE 18:2_18:3 | -1.48 | 2.26268E-05 | 0.36 | -1.03 | -4.66 | 3.78 |
| PE 18:2_20:3 | -1.19 | 5.70068E-05 | 0.44 | -0.82 | -4.44 | 2.78 |
| PE 18:2_20:4 | -1.01 | 0.016512895 | 0.5 | -0.7 | -2.69 | -3.33 |
| PE 18:2_22:4 | -1.33 | 1.25451E-05 | 0.4 | -0.92 | -4.82 | 4.49 |
| PE O-16:0/20:4 | -1.1 | 4.45538E-07 | 0.47 | -0.76 | -5.69 | 8.98 |
| PE P-16:0/16:1 | 1.18 | 4.45538E-07 | 2.27 | 0.82 | 5.65 | 8.73 |
| PE P-16:0/20:4 | -1.06 | 1.64895E-06 | 0.48 | -0.73 | -5.31 | 6.95 |
| PE P-16:0/22:4 | -1.13 | 4.45538E-07 | 0.46 | -0.78 | -5.64 | 8.68 |
| PE P-18:0/22:4 | -1.07 | 1.09184E-06 | 0.48 | -0.74 | -5.44 | 7.58 |
| PE P-18:1/18:2 | -1.03 | 1.78789E-05 | 0.49 | -0.72 | -4.73 | 4.09 |
| PE P-18:1/20:4 | -1.07 | 6.96235E-05 | 0.47 | -0.74 | -4.37 | 2.5 |
| PE P-18:1/22:4 | -1.03 | 7.19425E-05 | 0.49 | -0.72 | -4.36 | 2.45 |
| PE P-18:2/18:2 | -1.36 | 1.59323E-06 | 0.39 | -0.94 | -5.33 | 7.03 |
| PE P-18:2/20:4 | -1.53 | 9.13448E-07 | 0.35 | -1.06 | -5.48 | 7.82 |

**# log2FC, log2 fold change, adj.P.Val, adjusted P value, FC, fold change, lnFC, ln fold change.**

**Table S7. The plasma lipidomics difference between the CHF and Con**

| Lipid | log2FC | adj.P.Val | FC | lnFC | t | B |
| --- | --- | --- | --- | --- | --- | --- |
| Cer 18:0;O2/16:0 | 1.09 | 0.000380005 | 2.13 | 0.76 | 4.26 | 2.41 |
| Cer 18:0;O2/24:1 | 1.07 | 0.00016417 | 2.1 | 0.74 | 4.5 | 3.44 |
| Cer 18:1;O2/24:1 | 2.05 | 0.003856548 | 4.15 | 1.42 | 3.52 | -0.33 |
| GlcCer 18:1;O2/12:0 | 1.01 | 0.001444863 | 2.01 | 0.7 | 3.87 | 0.9 |
| LPE 16:0 | 1.04 | 0.001237113 | 2.06 | 0.72 | 3.92 | 1.08 |
| LPE 16:1 | 1.25 | 0.005336415 | 2.38 | 0.87 | 3.4 | -0.75 |
| LPE 18:0 | 1.11 | 0.006737653 | 2.16 | 0.77 | 3.31 | -1.04 |
| LPE 18:1 | 1.34 | 0.000364025 | 2.53 | 0.93 | 4.28 | 2.49 |
| LPE 18:3 | 1.37 | 0.001429447 | 2.58 | 0.95 | 3.88 | 0.93 |
| LPE 20:0 | 1.01 | 0.002684293 | 2.01 | 0.7 | 3.65 | 0.12 |
| LPE 20:1 | 1.45 | 0.000563489 | 2.74 | 1.01 | 4.14 | 1.94 |
| LPE 20:2 | 1.2 | 0.004578224 | 2.29 | 0.83 | 3.45 | -0.56 |
| LPE 22:4 | 1.61 | 0.00221444 | 3.06 | 1.12 | 3.72 | 0.38 |
| LPE 22:5 | 1.02 | 0.005963088 | 2.03 | 0.71 | 3.35 | -0.91 |
| PE 14:0_18:1 | 1.62 | 3.74671E-06 | 3.08 | 1.13 | 5.69 | 9.2 |
| PE 16:0_16:0 | 2.01 | 5.26584E-05 | 4.02 | 1.39 | 5.03 | 5.82 |
| PE 16:0_16:1 | 1.34 | 0.019208687 | 2.54 | 0.93 | 2.91 | -2.21 |
| PE 16:0_18:0 | 2.11 | 0.000138245 | 4.31 | 1.46 | 4.6 | 3.85 |
| PE 16:0_18:1 | 2.17 | 0.00029336 | 4.51 | 1.51 | 4.36 | 2.83 |
| PE 16:0_18:2 | 1.12 | 0.00181215 | 2.17 | 0.78 | 3.8 | 0.63 |
| PE 16:0_18:3 | 1.58 | 0.001819029 | 2.98 | 1.09 | 3.79 | 0.61 |
| PE 16:0_20:2 | 1.46 | 0.000364025 | 2.75 | 1.01 | 4.29 | 2.53 |
| PE 16:0_20:3 | 1.13 | 0.010598381 | 2.19 | 0.79 | 3.15 | -1.53 |
| PE 16:0_20:4 | 1.26 | 0.000364025 | 2.4 | 0.87 | 4.27 | 2.48 |
| PE 16:0_22:4 | 2.15 | 0.005453268 | 4.45 | 1.49 | 3.38 | -0.79 |
| PE 16:0_22:5 | 1.18 | 0.002456612 | 2.27 | 0.82 | 3.68 | 0.22 |
| PE 16:1_18:1 | 1.37 | 0.004650756 | 2.59 | 0.95 | 3.44 | -0.59 |
| PE 18:0_22:4 | 1.25 | 0.000141603 | 2.37 | 0.86 | 4.57 | 3.72 |
| PE 18:1_18:1 | 1.7 | 6.0511E-05 | 3.26 | 1.18 | 4.89 | 5.16 |
| PE 18:2_18:2 | 2.08 | 6.0511E-05 | 4.22 | 1.44 | 4.88 | 5.13 |
| PE O-16:0/22:4 | 1.27 | 0.00105324 | 2.4 | 0.88 | 3.97 | 1.28 |
| PE O-18:0/18:1 | 1.41 | 7.79137E-05 | 2.65 | 0.98 | 4.75 | 4.53 |
| PE O-18:0/22:4 | 1.93 | 0.00039855 | 3.81 | 1.34 | 4.24 | 2.34 |
| PE P-16:0/16:0 | 1.18 | 3.74671E-06 | 2.26 | 0.82 | 5.64 | 8.95 |
| PE P-16:0/18:1 | 1.3 | 7.16896E-05 | 2.46 | 0.9 | 4.81 | 4.8 |
| PE P-16:0/22:4 | 1.67 | 0.000428404 | 3.18 | 1.16 | 4.22 | 2.24 |
| PE P-18:0/16:1 | 1.1 | 6.49598E-05 | 2.14 | 0.76 | 4.85 | 4.98 |
| PE P-18:0/18:1 | 1.19 | 0.003080616 | 2.28 | 0.82 | 3.61 | -0.04 |
| PE P-18:0/20:2 | 1.21 | 0.00324084 | 2.32 | 0.84 | 3.58 | -0.14 |
| PE P-18:0/22:4 | 2.37 | 0.002425873 | 5.17 | 1.64 | 3.69 | 0.24 |
| PE P-18:0/22:5 | 1.27 | 0.001128056 | 2.42 | 0.88 | 3.95 | 1.19 |
| PE P-18:1/16:0 | 1.23 | 6.0511E-05 | 2.35 | 0.85 | 4.89 | 5.17 |
| PE P-18:1/22:4 | 2.23 | 0.003777071 | 4.69 | 1.55 | 3.53 | -0.29 |

**# log2FC, log2 fold change, adj.P.Val, adjusted P value, FC, fold change, lnFC, ln fold change.**

**Table S8. The logistic regression of RBC membrane different lipid**

| Lipid | Model 1 |  | Model 2 |  | Model 3 |  | Model 4 |  |
| --- | --- | --- | --- | --- | --- | --- | --- | --- |
|  | OR (95% CI) | *P* value | OR (95% CI) | *P* value | OR (95% CI) | *P* value | OR (95% CI) | *P* value |
| Cer 18:0;O2/16:0 | 1.09 (1.06, 1.13) | <0.001 | 1.10 (1.06, 1.14) | <0.001 | 1.11 (1.07, 1.15) | <0.001 | 1.07 (1.03, 1.12) | 0.001 |
| GlcCer 18:0;O2/18:0 | 0.92 (0.89, 0.95) | <0.001 | 0.92 (0.89, 0.95) | <0.001 | 0.93 (0.89, 0.96) | <0.001 | 0.92 (0.89, 0.96) | <0.001 |
| GlcCer 18:1;O2/14:0 | 1.07 (1.04, 1.10) | <0.001 | 1.07 (1.04, 1.11) | <0.001 | 1.07 (1.04, 1.11) | <0.001 | 1.08 (1.05, 1.12) | <0.001 |
| GlcCer 18:1;O2/16:0 | 1.06 (1.03, 1.09) | <0.001 | 1.05 (1.02, 1.09) | 0.001 | 1.06 (1.03, 1.10) | <0.001 | 1.05 (1.01, 1.09) | 0.016 |
| LPE 16:0 | 1.05 (1.03, 1.07) | <0.001 | 1.05 (1.03, 1.07) | <0.001 | 1.04 (1.02, 1.06) | <0.001 | 1.04 (1.02, 1.07) | <0.001 |
| LPE 16:1 | 1.03 (1.02, 1.04) | <0.001 | 1.03 (1.01, 1.04) | <0.001 | 1.02 (1.01, 1.04) | <0.001 | 1.03 (1.01, 1.04) | <0.001 |
| LPE 18:0 | 1.07 (1.05, 1.10) | <0.001 | 1.07 (1.04, 1.10) | <0.001 | 1.06 (1.03, 1.09) | <0.001 | 1.07 (1.04, 1.10) | <0.001 |
| LPE 18:1 | 1.04 (1.02, 1.05) | <0.001 | 1.04 (1.02, 1.06) | <0.001 | 1.03 (1.02, 1.05) | <0.001 | 1.04 (1.02, 1.05) | <0.001 |
| LPE 20:0 | 1.05 (1.03, 1.07) | <0.001 | 1.05 (1.03, 1.07) | <0.001 | 1.04 (1.03, 1.07) | <0.001 | 1.04 (1.02, 1.07) | <0.001 |
| LPE 20:1 | 1.05 (1.03, 1.07) | <0.001 | 1.05 (1.03, 1.07) | <0.001 | 1.05 (1.03, 1.07) | <0.001 | 1.05 (1.03, 1.07) | <0.001 |
| LPE 20:2 | 1.03 (1.02, 1.05) | <0.001 | 1.04 (1.02, 1.06) | <0.001 | 1.04 (1.02, 1.06) | <0.001 | 1.05 (1.02, 1.07) | <0.001 |
| PE 18:1_22:4 | 0.93 (0.91, 0.96) | <0.001 | 0.95 (0.92, 0.98) | <0.001 | 0.96 (0.93, 1.00) | 0.025 | 0.97 (0.94, 1.01) | 0.15 |
| PE 18:2_18:2 | 0.93 (0.90, 0.95) | <0.001 | 0.94 (0.91, 0.97) | <0.001 | 0.95 (0.92, 0.98) | 0.004 | 0.97 (0.93, 1.00) | 0.077 |
| PE 18:2_18:3 | 0.94 (0.92, 0.97) | <0.001 | 0.95 (0.92, 0.97) | <0.001 | 0.95 (0.93, 0.98) | <0.001 | 0.97 (0.94, 1.00) | 0.025 |
| PE 18:2_20:3 | 0.94 (0.91, 0.96) | <0.001 | 0.94 (0.92, 0.97) | <0.001 | 0.96 (0.93, 0.99) | 0.010 | 0.98 (0.94, 1.01) | 0.2 |
| PE 18:2_20:4 | 0.97 (0.95, 0.99) | 0.007 | 0.98 (0.96, 1.00) | 0.022 | 0.98 (0.96, 1.00) | 0.089 | 0.99 (0.97, 1.02) | 0.5 |
| PE 18:2_22:4 | 0.94 (0.91, 0.96) | <0.001 | 0.95 (0.92, 0.97) | <0.001 | 0.96 (0.93, 0.99) | 0.006 | 0.97 (0.94, 1.00) | 0.079 |
| PE O-16:0/20:4 | 0.89 (0.86, 0.93) | <0.001 | 0.92 (0.88, 0.96) | <0.001 | 0.93 (0.88, 0.97) | 0.001 | 0.94 (0.89, 0.98) | 0.011 |
| PE P-16:0/16:1 | 1.11 (1.07, 1.15) | <0.001 | 1.09 (1.05, 1.13) | <0.001 | 1.09 (1.05, 1.14) | <0.001 | 1.07 (1.02, 1.12) | 0.003 |
| PE P-16:0/20:4 | 0.90 (0.87, 0.94) | <0.001 | 0.92 (0.88, 0.96) | <0.001 | 0.93 (0.89, 0.97) | <0.001 | 0.95 (0.90, 0.99) | 0.020 |
| PE P-16:0/22:4 | 0.90 (0.86, 0.93) | <0.001 | 0.92 (0.88, 0.96) | <0.001 | 0.94 (0.90, 0.98) | 0.002 | 0.95 (0.91, 0.99) | 0.049 |
| PE P-18:0/22:4 | 0.90 (0.86, 0.93) | <0.001 | 0.92 (0.88, 0.96) | <0.001 | 0.93 (0.89, 0.97) | <0.001 | 0.94 (0.90, 0.99) | 0.010 |
| PE P-18:1/18:2 | 0.92 (0.88, 0.95) | <0.001 | 0.93 (0.89, 0.97) | <0.001 | 0.94 (0.90, 0.98) | 0.002 | 0.95 (0.91, 0.99) | 0.036 |
| PE P-18:1/20:4 | 0.93 (0.91, 0.96) | <0.001 | 0.94 (0.91, 0.97) | <0.001 | 0.94 (0.91, 0.97) | <0.001 | 0.96 (0.93, 0.99) | 0.038 |
| PE P-18:1/22:4 | 0.93 (0.90, 0.96) | <0.001 | 0.93 (0.90, 0.96) | <0.001 | 0.94 (0.91, 0.98) | <0.001 | 0.96 (0.93, 1.00) | 0.052 |
| PE P-18:2/18:2 | 0.92 (0.89, 0.95) | <0.001 | 0.93 (0.90, 0.96) | <0.001 | 0.94 (0.90, 0.97) | <0.001 | 0.96 (0.93, 0.98) | 0.004 |
| PE P-18:2/20:4 | 0.93 (0.90, 0.95) | <0.001 | 0.93 (0.90, 0.96) | <0.001 | 0.93 (0.91, 0.96) | <0.001 | 0.96 (0.93, 0.99) | 0.008 |

**#The Logistic regression was adjusted with four models, the adjusted details of the four models included Model 1: no adjustment; Model 2: age + sex; Model 3: Model 2 + CVD + AF + DM + Hyperlipidemia + Hyperuricemia; Model 4: Model 3 + CKD + Hypertension + Anemia + COPD + Globulin + Glucose + Albumin + Total triglycerides + ALT + Lym + Platelets + WBC + Smoking history + Alcohol History + ACEI/ARB + Beta-blockers + CCB + Metformin + Insulin injections + Statins + Diuretics.**

**Table S9.** **The logistic regression of plasma different lipid**

| Characteristic | Model 1 |  | Model 2 |  | Model 3 |  | Model 4 |  |
| --- | --- | --- | --- | --- | --- | --- | --- | --- |
|  | OR (95% CI) | *P* value | OR (95% CI) | *P* value | OR (95% CI) | *P* value | OR (95% CI) | *P* value |
| Cer 18:0;O2/16:0 | 1.06 (1.03, 1.10) | <0.001 | 1.06 (1.02, 1.09) | <0.001 | 1.04 (1.01, 1.08) | 0.016 | 1.05 (1.01, 1.08) | 0.016 |
| Cer 18:0;O2/24:1 | 1.07 (1.04, 1.11) | <0.001 | 1.07 (1.03, 1.10) | <0.001 | 1.05 (1.02, 1.09) | 0.003 | 1.05 (1.01, 1.09) | 0.010 |
| Cer 18:1;O2/24:1 | 1.02 (1.01, 1.04) | <0.001 | 1.02 (1.00, 1.03) | 0.018 | 1.01 (1.00, 1.03) | 0.11 | 1.01 (0.99, 1.02) | 0.3 |
| GlcCer 18:1;O2/12:0 | 1.06 (1.03, 1.09) | <0.001 | 1.05 (1.02, 1.08) | 0.001 | 1.04 (1.01, 1.07) | 0.019 | 1.04 (1.00, 1.08) | 0.026 |
| LPE 16:0 | 1.06 (1.03, 1.09) | <0.001 | 1.06 (1.03, 1.09) | <0.001 | 1.05 (1.02, 1.09) | 0.002 | 1.06 (1.02, 1.10) | 0.002 |
| LPE 16:1 | 1.04 (1.02, 1.07) | <0.001 | 1.04 (1.01, 1.07) | 0.002 | 1.04 (1.02, 1.07) | 0.003 | 1.04 (1.01, 1.07) | 0.007 |
| LPE 18:0 | 1.04 (1.02, 1.06) | 0.001 | 1.04 (1.02, 1.07) | 0.001 | 1.04 (1.01, 1.06) | 0.006 | 1.04 (1.01, 1.07) | 0.010 |
| LPE 18:1 | 1.05 (1.03, 1.08) | <0.001 | 1.05 (1.03, 1.08) | <0.001 | 1.05 (1.02, 1.08) | 0.002 | 1.04 (1.01, 1.08) | 0.005 |
| LPE 18:3 | 1.04 (1.02, 1.07) | <0.001 | 1.04 (1.02, 1.07) | <0.001 | 1.04 (1.01, 1.06) | 0.004 | 1.03 (1.01, 1.06) | 0.014 |
| LPE 20:0 | 1.05 (1.02, 1.08) | <0.001 | 1.05 (1.02, 1.09) | <0.001 | 1.05 (1.02, 1.08) | 0.003 | 1.05 (1.01, 1.08) | 0.009 |
| LPE 20:1 | 1.05 (1.03, 1.08) | <0.001 | 1.06 (1.03, 1.09) | <0.001 | 1.05 (1.02, 1.08) | <0.001 | 1.05 (1.02, 1.08) | 0.002 |
| LPE 20:2 | 1.06 (1.03, 1.09) | <0.001 | 1.05 (1.02, 1.09) | <0.001 | 1.05 (1.02, 1.09) | 0.003 | 1.04 (1.01, 1.08) | 0.027 |
| LPE 22:4 | 1.04 (1.02, 1.07) | <0.001 | 1.04 (1.02, 1.07) | <0.001 | 1.04 (1.02, 1.07) | <0.001 | 1.04 (1.02, 1.07) | 0.002 |
| LPE 22:5 | 1.05 (1.02, 1.08) | 0.001 | 1.05 (1.02, 1.08) | 0.001 | 1.05 (1.02, 1.09) | 0.002 | 1.04 (1.01, 1.08) | 0.019 |
| PE 14:0_18:1 | 1.08 (1.05, 1.11) | <0.001 | 1.08 (1.04, 1.11) | <0.001 | 1.07 (1.04, 1.10) | <0.001 | 1.07 (1.03, 1.11) | <0.001 |
| PE 16:0_16:0 | 1.06 (1.03, 1.09) | <0.001 | 1.05 (1.03, 1.08) | <0.001 | 1.04 (1.02, 1.07) | <0.001 | 1.05 (1.02, 1.07) | <0.001 |
| PE 16:0_16:1 | 1.02 (1.01, 1.04) | 0.005 | 1.02 (1.00, 1.04) | 0.016 | 1.02 (1.00, 1.04) | 0.017 | 1.03 (1.01, 1.05) | 0.010 |
| PE 16:0_18:0 | 1.05 (1.03, 1.07) | <0.001 | 1.04 (1.02, 1.06) | <0.001 | 1.03 (1.01, 1.05) | 0.003 | 1.03 (1.01, 1.06) | 0.003 |
| PE 16:0_18:1 | 1.04 (1.02, 1.06) | <0.001 | 1.03 (1.02, 1.06) | <0.001 | 1.03 (1.01, 1.05) | 0.004 | 1.03 (1.01, 1.05) | 0.003 |
| PE 16:0_18:2 | 1.05 (1.02, 1.08) | <0.001 | 1.04 (1.02, 1.07) | 0.002 | 1.04 (1.01, 1.07) | 0.009 | 1.05 (1.01, 1.08) | 0.004 |
| PE 16:0_18:3 | 1.04 (1.02, 1.06) | <0.001 | 1.03 (1.01, 1.05) | 0.002 | 1.03 (1.01, 1.05) | 0.010 | 1.03 (1.01, 1.05) | 0.011 |
| PE 16:0_20:2 | 1.05 (1.03, 1.08) | <0.001 | 1.05 (1.02, 1.08) | <0.001 | 1.04 (1.01, 1.07) | 0.004 | 1.04 (1.01, 1.07) | 0.004 |
| PE 16:0_20:3 | 1.03 (1.01, 1.06) | 0.002 | 1.03 (1.01, 1.06) | 0.006 | 1.03 (1.01, 1.05) | 0.017 | 1.03 (1.01, 1.06) | 0.009 |
| PE 16:0_20:4 | 1.06 (1.03, 1.09) | <0.001 | 1.06 (1.03, 1.09) | <0.001 | 1.05 (1.02, 1.08) | 0.001 | 1.06 (1.03, 1.09) | <0.001 |
| PE 16:0_22:4 | 1.03 (1.01, 1.04) | 0.002 | 1.02 (1.01, 1.04) | 0.007 | 1.02 (1.00, 1.04) | 0.028 | 1.02 (1.00, 1.04) | 0.023 |
| PE 16:0_22:5 | 1.05 (1.02, 1.07) | <0.001 | 1.04 (1.02, 1.07) | <0.001 | 1.04 (1.01, 1.07) | 0.005 | 1.04 (1.01, 1.08) | 0.004 |
| PE 16:1_18:1 | 1.03 (1.01, 1.06) | 0.001 | 1.03 (1.01, 1.05) | 0.004 | 1.03 (1.01, 1.05) | 0.007 | 1.03 (1.01, 1.06) | 0.011 |
| PE 18:0_22:4 | 1.07 (1.04, 1.10) | <0.001 | 1.07 (1.04, 1.11) | <0.001 | 1.07 (1.03, 1.10) | <0.001 | 1.07 (1.04, 1.11) | <0.001 |
| PE 18:1_18:1 | 1.06 (1.03, 1.08) | <0.001 | 1.05 (1.03, 1.08) | <0.001 | 1.05 (1.02, 1.07) | <0.001 | 1.05 (1.02, 1.08) | <0.001 |
| PE 18:2_18:2 | 1.05 (1.03, 1.07) | <0.001 | 1.04 (1.02, 1.06) | <0.001 | 1.03 (1.01, 1.05) | 0.002 | 1.04 (1.01, 1.06) | 0.001 |
| PE O-16:0/22:4 | 1.05 (1.02, 1.08) | <0.001 | 1.06 (1.03, 1.09) | <0.001 | 1.05 (1.02, 1.08) | 0.001 | 1.05 (1.02, 1.08) | 0.002 |
| PE O-18:0/18:1 | 1.07 (1.04, 1.10) | <0.001 | 1.06 (1.03, 1.10) | <0.001 | 1.05 (1.02, 1.08) | <0.001 | 1.04 (1.01, 1.08) | 0.008 |
| PE O-18:0/22:4 | 1.05 (1.02, 1.08) | <0.001 | 1.05 (1.02, 1.08) | <0.001 | 1.04 (1.01, 1.07) | 0.005 | 1.04 (1.01, 1.06) | 0.006 |
| PE P-16:0/16:0 | 1.11 (1.07, 1.15) | <0.001 | 1.10 (1.06, 1.14) | <0.001 | 1.08 (1.04, 1.13) | <0.001 | 1.08 (1.03, 1.13) | <0.001 |
| PE P-16:0/18:1 | 1.07 (1.04, 1.11) | <0.001 | 1.08 (1.05, 1.12) | <0.001 | 1.07 (1.03, 1.11) | <0.001 | 1.07 (1.03, 1.11) | <0.001 |
| PE P-16:0/22:4 | 1.05 (1.03, 1.08) | <0.001 | 1.06 (1.03, 1.09) | <0.001 | 1.05 (1.02, 1.08) | 0.001 | 1.05 (1.02, 1.08) | 0.002 |
| PE P-18:0/16:1 | 1.09 (1.05, 1.12) | <0.001 | 1.10 (1.06, 1.14) | <0.001 | 1.09 (1.05, 1.13) | <0.001 | 1.09 (1.05, 1.14) | <0.001 |
| PE P-18:0/18:1 | 1.05 (1.02, 1.07) | <0.001 | 1.05 (1.03, 1.09) | <0.001 | 1.05 (1.02, 1.08) | 0.001 | 1.05 (1.02, 1.09) | 0.002 |
| PE P-18:0/20:2 | 1.04 (1.02, 1.07) | <0.001 | 1.05 (1.02, 1.08) | 0.001 | 1.05 (1.02, 1.08) | 0.002 | 1.04 (1.02, 1.08) | 0.005 |
| PE P-18:0/22:4 | 1.03 (1.01, 1.05) | 0.002 | 1.03 (1.02, 1.06) | 0.001 | 1.03 (1.01, 1.05) | 0.010 | 1.03 (1.01, 1.05) | 0.010 |
| PE P-18:0/22:5 | 1.05 (1.02, 1.08) | <0.001 | 1.06 (1.03, 1.10) | <0.001 | 1.06 (1.02, 1.09) | <0.001 | 1.06 (1.02, 1.09) | 0.001 |
| PE P-18:1/16:0 | 1.08 (1.05, 1.12) | <0.001 | 1.08 (1.05, 1.12) | <0.001 | 1.07 (1.04, 1.11) | <0.001 | 1.07 (1.03, 1.12) | <0.001 |
| PE P-18:1/22:4 | 1.02 (1.01, 1.04) | 0.001 | 1.02 (1.01, 1.04) | 0.003 | 1.02 (1.00, 1.03) | 0.014 | 1.02 (1.00, 1.03) | 0.023 |

**#The Logistic regression was adjusted with four models, the adjusted details of the four models included Model 1: no adjustment; Model 2: age + sex; Model 3: Model 2 + CVD + AF + DM + Hyperlipidemia + Hyperuricemia; Model 4: Model 3 + CKD + Hypertension + Anemia + COPD + Globulin + Glucose + Albumin + Total triglycerides + ALT + Lym + Platelets + WBC + Smoking history + Alcohol History + ACEI/ARB + Beta-blockers + CCB + Metformin + Insulin injections + Statins + Diuretics.**

**Table S10. The baseline of CHF in ePVS normal vs ePVS overload**

| Characteristic | Overall | ePVS normal | ePVS overload | *P* value |
| --- | --- | --- | --- | --- |
|  | N = 684 | N = 463 | N = 221 |  |
| Demographic |  |  |  |  |
| Age, year | 70.00 (59.00, 77.50) | 70.00 (57.00, 76.00) | 72.00 (63.00, 79.00) | 0.003 |
| Sex, male, n(%) | 383 (56%) | 285 (62%) | 98 (44%) | <0.001 |
| Medical history |  |  |  |  |
| CVD, n(%) | 669 (98%) | 457 (99%) | 212 (96%) | 0.026 |
| AF, n(%) | 143 (21%) | 88 (19%) | 55 (25%) | 0.077 |
| Stroke, n(%) | 92 (13%) | 56 (12%) | 36 (16%) | 0.13 |
| DM, n(%) | 221 (32%) | 140 (30%) | 81 (37%) | 0.093 |
| Hyperlipidemia, n(%) | 147 (21%) | 101 (22%) | 46 (21%) | 0.8 |
| Hyperuricemia, n(%) | 52 (7.6%) | 30 (6.5%) | 22 (10.0%) | 0.11 |
| CKD, n(%) | 197 (29%) | 98 (21%) | 99 (45%) | <0.001 |
| Hypertension, n(%) | 459 (67%) | 302 (65%) | 157 (71%) | 0.13 |
| COPD, n(%) | 48 (7.0%) | 30 (6.5%) | 18 (8.1%) | 0.4 |
| Anemia, n(%) | 113 (17%) | 19 (4.1%) | 94 (43%) | <0.001 |
| Life style |  |  |  |  |
| Smoking history, n(%) | 116 (17%) | 74 (16%) | 42 (19%) | 0.3 |
| Alcohol history, n(%) | 138 (20%) | 88 (19%) | 50 (23%) | 0.3 |
| Laboratory tests |  |  |  |  |
| BNP, ng L^-1^ | 334.26 (232.66, 659.93) | 290.00 (214.00, 378.00) | 739.54 (426.00, 1,586.18) | <0.001 |
| ESI, cL g^-1^ | 4.02 (3.74, 4.36) | 3.94 (3.70, 4.22) | 4.18 (3.87, 4.64) | <0.001 |
| ePVS, mL g^-1^ | 4.63 (3.84, 6.15) | 4.14 (3.53, 4.66) | 7.90 (6.20, 12.40) | <0.001 |
| RBC count, m uL^-1^ | 4.40 (3.86, 4.89) | 4.58 (4.21, 5.04) | 3.64 (3.24, 4.20) | <0.001 |
| Hematocrit, % | 40.30 (35.55, 44.00) | 41.50 (39.10, 45.50) | 33.50 (29.60, 37.60) | <0.001 |
| RDW, % | 13.40 (12.70, 14.40) | 13.20 (12.60, 14.00) | 13.80 (13.10, 15.10) | <0.001 |
| Hemoglobin, g dL^-1^ | 135.00 (118.00, 151.00) | 141.00 (130.00, 155.00) | 111.00 (97.00, 126.00) | <0.001 |
| MCV, fL | 92.00 (88.40, 95.30) | 92.30 (88.60, 95.30) | 91.40 (87.80, 95.90) | 0.2 |
| MCH, pg | 30.90 (29.60, 32.10) | 31.00 (29.70, 32.10) | 30.30 (28.60, 31.90) | <0.001 |
| MCHC, g dL^-1^ | 334.00 (325.00, 343.00) | 336.00 (328.00, 344.00) | 328.00 (320.00, 340.00) | <0.001 |
| Globulin, g L^-1^ | 24.65 (21.70, 27.75) | 24.60 (21.90, 27.40) | 24.80 (21.60, 28.20) | 0.5 |
| Glucose, mmol L^-1^ | 6.49 (5.30, 8.10) | 6.46 (5.31, 7.82) | 6.62 (5.21, 8.62) | 0.4 |
| Basophils count, K μL^-1^ | 0.02 (0.01, 0.03) | 0.02 (0.01, 0.03) | 0.02 (0.01, 0.03) | 0.086 |
| Albumin, g L^-1^ | 40.80 (37.60, 44.20) | 41.80 (38.80, 44.90) | 39.30 (34.80, 42.30) | <0.001 |
| Platelet count, K uL^-1^ | 219.00 (172.00, 266.00) | 219.00 (176.00, 264.00) | 219.00 (166.00, 273.00) | >0.9 |
| WBC, K uL^-1^ | 7.79 (5.99, 10.29) | 7.97 (6.12, 10.49) | 7.42 (5.82, 10.10) | 0.047 |
| ALT, U L^-1^ | 20.00 (13.00, 35.00) | 22.00 (13.00, 37.00) | 17.00 (11.00, 32.00) | <0.001 |
| AST, U L^-1^ | 25.00 (17.00, 65.00) | 27.00 (18.00, 83.00) | 23.00 (15.00, 46.00) | <0.001 |
| IDBL, μmol L^-1^ | 5.90 (3.60, 9.35) | 6.40 (4.30, 9.70) | 4.60 (2.80, 8.10) | <0.001 |
| ALP, U L^-1^ | 81.00 (64.00, 100.00) | 80.00 (64.00, 100.00) | 81.00 (65.00, 104.00) | 0.3 |
| Lym, K μL^-1^ | 1.47 (1.01, 2.22) | 1.57 (1.13, 2.40) | 1.24 (0.88, 1.74) | <0.001 |
| MPV, fL | 9.90 (9.30, 10.80) | 9.90 (9.30, 10.80) | 10.00 (9.30, 10.90) | 0.7 |
| PDW, % | 11.55 (10.20, 13.20) | 11.50 (10.30, 13.10) | 11.60 (10.20, 13.70) | 0.5 |
| DBIL, μmol L^-1^ | 4.80 (3.50, 6.80) | 4.90 (3.70, 6.50) | 4.50 (3.40, 7.10) | 0.2 |
| TBI, μmol L^-1^ | 11.00 (7.30, 16.25) | 11.50 (7.80, 16.40) | 9.90 (6.40, 15.30) | 0.003 |
| TBA, μmol L^-1^ | 2.85 (1.70, 4.80) | 2.90 (1.70, 4.90) | 2.80 (1.70, 4.60) | 0.4 |
| TC, mmol L^-1^ | 4.44 (3.55, 5.26) | 4.50 (3.64, 5.25) | 4.19 (3.37, 5.26) | 0.064 |
| LDL, mmol L^-1^ | 2.71 (1.96, 3.44) | 2.78 (2.01, 3.46) | 2.52 (1.82, 3.43) | 0.077 |
| Total triglycerides, mmol L^-1^ | 1.16 (0.82, 1.67) | 1.16 (0.84, 1.70) | 1.15 (0.80, 1.62) | 0.5 |
| HDL, mmol L^-1^ | 1.12 (0.93, 1.33) | 1.13 (0.94, 1.34) | 1.10 (0.88, 1.31) | 0.14 |
| Medical therapy |  |  |  |  |
| ACEI/ARB, n(%) | 307 (45%) | 210 (45%) | 97 (44%) | 0.7 |
| Beta-blockers, n(%) | 297 (43%) | 197 (43%) | 100 (45%) | 0.5 |
| CCB, n(%) | 311 (45%) | 210 (45%) | 101 (46%) | >0.9 |
| Metformin, n(%) | 128 (19%) | 79 (17%) | 49 (22%) | 0.11 |
| Insulin injections, n(%) | 94 (14%) | 57 (12%) | 37 (17%) | 0.12 |
| Statins, n(%) | 108 (16%) | 79 (17%) | 29 (13%) | 0.2 |
| Diuretics, n(%) | 101 (15%) | 62 (13%) | 39 (18%) | 0.14 |

**#Continuous variables are expressed as interquartile ranges. Categorical variables are expressed as frequency (percentage). CVD, cardiovascular disease, AF, atrial fibrillation, DM, diabetes mellitus, CKD, chronic kidney disease, COPD, Chronic obstructive pulmonary disease, BNP, B-type natriuretic peptide, ESI, erythrocyte stress index, ePVS, estimated plasma volume status, RBC, red blood cell count, RDW, red blood cell distribution, MCV, mean cell volume, MCH, mean corpuscular hemoglobin, MCHC, mean corpuscular hemoglobin concentration, WBC, white blood cell count, ALT, alanine aminotransferase, AST, aspartate aminotransferase, IBIL, indirect bilirubin, ALP, alkaline phosphatase, MPV, mean platelet volume, PDW, platelet distribution width, DBIL, direct bilirubin, TBI, total bilirubin, TBA, total bile acids, TC, total cholesterol, LDL, low-density lipoprotein, HDL, high-density lipoprotein, Lym, lymphocyte count, ACEI/ARB, ACE-inhibitors/AT1-receptor antagonists, CCB, dihydropyridine Ca2^+^ channel blockers.**

**Table S11. The logistic regression of RBC membrane different lipids in ePVS normal vs ePVS overload**

| Characteristic | Model 1 |  | Model 2 |  | Model 3 |  | Model 4 |  |
| --- | --- | --- | --- | --- | --- | --- | --- | --- |
|  | OR (95% CI) | *P* value | OR (95% CI) | *P* value | OR (95% CI) | *P* value | OR (95% CI) | *P* value |
| Cer 18:0;O2/16:0 | 1.48 (1.38, 1.59) | <0.001 | 1.49 (1.39, 1.61) | <0.001 | 1.67 (1.52, 1.84) | <0.001 | 1.62 (1.47, 1.80) | <0.001 |
| GlcCer 18:0;O2/18:0 | 0.93 (0.88, 0.98) | 0.005 | 0.91 (0.86, 0.96) | <0.001 | 0.88 (0.82, 0.93) | <0.001 | 0.90 (0.84, 0.97) | 0.004 |
| GlcCer 18:1;O2/14:0 | 1.06 (1.02, 1.09) | 0.001 | 1.06 (1.03, 1.10) | <0.001 | 1.09 (1.05, 1.14) | <0.001 | 1.09 (1.04, 1.14) | <0.001 |
| GlcCer 18:1;O2/16:0 | 1.09 (1.04, 1.13) | <0.001 | 1.08 (1.04, 1.13) | <0.001 | 1.06 (1.01, 1.11) | 0.017 | 1.05 (1.00, 1.11) | 0.054 |
| LPE 16:0 | 1.06 (1.03, 1.08) | <0.001 | 1.06 (1.04, 1.09) | <0.001 | 1.08 (1.05, 1.11) | <0.001 | 1.06 (1.03, 1.09) | <0.001 |
| LPE 16:1 | 1.04 (1.02, 1.05) | <0.001 | 1.04 (1.02, 1.05) | <0.001 | 1.05 (1.03, 1.07) | <0.001 | 1.03 (1.01, 1.06) | 0.002 |
| LPE 18:0 | 1.11 (1.07, 1.15) | <0.001 | 1.12 (1.08, 1.16) | <0.001 | 1.13 (1.09, 1.18) | <0.001 | 1.11 (1.06, 1.16) | <0.001 |
| LPE 18:1 | 1.05 (1.03, 1.07) | <0.001 | 1.05 (1.03, 1.07) | <0.001 | 1.07 (1.04, 1.09) | <0.001 | 1.05 (1.02, 1.08) | <0.001 |
| LPE 20:0 | 1.07 (1.04, 1.09) | <0.001 | 1.08 (1.05, 1.10) | <0.001 | 1.09 (1.06, 1.12) | <0.001 | 1.07 (1.04, 1.10) | <0.001 |
| LPE 20:1 | 1.05 (1.02, 1.07) | <0.001 | 1.05 (1.03, 1.08) | <0.001 | 1.07 (1.04, 1.09) | <0.001 | 1.05 (1.02, 1.08) | <0.001 |
| LPE 20:2 | 1.05 (1.02, 1.08) | <0.001 | 1.05 (1.02, 1.08) | <0.001 | 1.06 (1.02, 1.09) | <0.001 | 1.04 (1.01, 1.07) | 0.023 |
| PE 18:1_22:4 | 0.96 (0.92, 1.01) | 0.13 | 0.96 (0.92, 1.01) | 0.14 | 0.89 (0.84, 0.95) | <0.001 | 0.91 (0.85, 0.97) | 0.004 |
| PE 18:2_18:2 | 0.92 (0.87, 0.97) | 0.002 | 0.92 (0.87, 0.97) | 0.004 | 0.90 (0.85, 0.96) | 0.002 | 0.94 (0.88, 1.01) | 0.082 |
| PE 18:2_18:3 | 0.93 (0.89, 0.98) | 0.004 | 0.93 (0.89, 0.98) | 0.005 | 0.89 (0.84, 0.95) | <0.001 | 0.93 (0.87, 0.98) | 0.013 |
| PE 18:2_20:3 | 0.93 (0.88, 0.98) | 0.004 | 0.93 (0.88, 0.98) | 0.004 | 0.87 (0.81, 0.92) | <0.001 | 0.89 (0.83, 0.94) | <0.001 |
| PE 18:2_20:4 | 0.99 (0.96, 1.02) | 0.6 | 0.99 (0.96, 1.02) | 0.6 | 0.98 (0.94, 1.02) | 0.3 | 0.99 (0.95, 1.03) | 0.6 |
| PE 18:2_22:4 | 0.95 (0.91, 1.00) | 0.050 | 0.96 (0.91, 1.00) | 0.080 | 0.89 (0.84, 0.95) | <0.001 | 0.92 (0.86, 0.97) | 0.006 |
| PE O-16:0/20:4 | 0.98 (0.91, 1.04) | 0.5 | 0.96 (0.90, 1.03) | 0.3 | 0.88 (0.81, 0.96) | 0.003 | 0.90 (0.82, 0.98) | 0.021 |
| PE P-16:0/16:1 | 1.17 (1.11, 1.24) | <0.001 | 1.17 (1.11, 1.23) | <0.001 | 1.16 (1.09, 1.24) | <0.001 | 1.14 (1.06, 1.22) | <0.001 |
| PE P-16:0/20:4 | 0.98 (0.92, 1.04) | 0.5 | 0.97 (0.91, 1.03) | 0.3 | 0.91 (0.85, 0.98) | 0.018 | 0.94 (0.87, 1.02) | 0.12 |
| PE P-16:0/22:4 | 0.95 (0.89, 1.01) | 0.083 | 0.95 (0.89, 1.01) | 0.084 | 0.90 (0.83, 0.96) | 0.004 | 0.93 (0.86, 1.00) | 0.052 |
| PE P-18:0/22:4 | 0.95 (0.90, 1.01) | 0.14 | 0.96 (0.90, 1.02) | 0.2 | 0.94 (0.87, 1.01) | 0.10 | 0.97 (0.89, 1.05) | 0.4 |
| PE P-18:1/18:2 | 0.99 (0.93, 1.04) | 0.6 | 0.99 (0.93, 1.05) | 0.7 | 0.96 (0.90, 1.03) | 0.3 | 0.97 (0.90, 1.05) | 0.5 |
| PE P-18:1/20:4 | 0.96 (0.92, 1.01) | 0.14 | 0.96 (0.91, 1.01) | 0.087 | 0.91 (0.85, 0.96) | 0.002 | 0.93 (0.87, 0.99) | 0.029 |
| PE P-18:1/22:4 | 0.98 (0.93, 1.03) | 0.5 | 0.98 (0.93, 1.03) | 0.4 | 0.94 (0.89, 1.00) | 0.064 | 0.96 (0.90, 1.02) | 0.2 |
| PE P-18:2/18:2 | 0.91 (0.86, 0.97) | 0.002 | 0.92 (0.87, 0.97) | 0.005 | 0.91 (0.85, 0.97) | 0.006 | 0.93 (0.87, 1.00) | 0.066 |
| PE P-18:2/20:4 | 0.94 (0.90, 0.99) | 0.015 | 0.94 (0.89, 0.99) | 0.012 | 0.91 (0.85, 0.96) | <0.001 | 0.93 (0.87, 0.99) | 0.018 |

**#The Logistic regression was adjusted with four models, the adjusted details of the four models included Model 1: no adjustment; Model 2: age + sex; Model 3: Model 2 + CVD + CKD + Anemia; Model 4: Model 3 + BNP + Albumin + WBC + ALT + IDBL + Lym + TBIL.**

**Table S12. The baseline characteristic of CHF patients with MACE or not**

| Characteristic | Overall | no MACE | MACE | *P* value |
| --- | --- | --- | --- | --- |
|  | N = 684 | N = 497 | N = 187 |  |
| Demographic |  |  |  |  |
| Age, year | 70.00 (59.00, 77.50) | 70.00 (58.00, 77.00) | 70.00 (59.00, 78.00) | 0.5 |
| Sex, male, n(%) | 383 (56%) | 282 (57%) | 101 (54%) | 0.5 |
| Follow-up time (days) | 521.00 (132.00, 743.00) | 660.00 (174.00, 783.00) | 191.00 (56.00, 507.00) | <0.001 |
| Medical history |  |  |  |  |
| CVD, n(%) | 669 (98%) | 482 (97%) | 187 (100%) | 0.015 |
| AF, n(%) | 143 (21%) | 109 (22%) | 34 (18%) | 0.3 |
| Stroke, n(%) | 92 (13%) | 73 (15%) | 19 (10%) | 0.12 |
| DM, n(%) | 221 (32%) | 165 (33%) | 56 (30%) | 0.4 |
| Hyperlipidemia, n(%) | 147 (21%) | 106 (21%) | 41 (22%) | 0.9 |
| Hyperuricemia, n(%) | 52 (7.6%) | 38 (7.6%) | 14 (7.5%) | >0.9 |
| CKD, n(%) | 197 (29%) | 137 (28%) | 60 (32%) | 0.2 |
| Hypertension, n(%) | 459 (67%) | 327 (66%) | 132 (71%) | 0.2 |
| COPD, n(%) | 48 (7.0%) | 26 (5.2%) | 22 (12%) | 0.003 |
| Anemia, n(%) | 113 (17%) | 73 (15%) | 40 (21%) | 0.035 |
| Life style |  |  |  |  |
| Smoking history, n(%) | 118 (17%) | 81 (16%) | 37 (20%) | 0.3 |
| Alcohol history, n(%) | 142 (21%) | 95 (19%) | 47 (25%) | 0.084 |
| Laboratory tests |  |  |  |  |
| BNP, ng L^-1^ | 334.26 (232.66, 659.93) | 323.10 (230.00, 476.59) | 375.00 (248.10, 829.63) | 0.019 |
| ESI, cL g^-1^ | 4.02 (3.74, 4.36) | 3.91 (3.67, 4.27) | 4.17 (3.97, 4.55) | <0.001 |
| ePVS, mL g^-1^ | 4.63 (3.84, 6.15) | 4.58 (3.85, 5.89) | 4.97 (3.81, 6.70) | 0.088 |
| RBC count, m uL^-1^ | 4.40 (3.86, 4.89) | 4.44 (3.87, 4.89) | 4.26 (3.76, 4.94) | 0.3 |
| Hematocrit, % | 40.30 (35.55, 44.00) | 40.40 (36.00, 44.00) | 39.90 (34.60, 44.30) | 0.2 |
| RDW, % | 13.40 (12.70, 14.40) | 13.20 (12.60, 14.00) | 13.90 (13.30, 14.90) | <0.001 |
| Hemoglobin, g dL^-1^ | 135.00 (118.00, 151.00) | 136.00 (120.00, 151.00) | 132.00 (113.00, 151.00) | 0.044 |
| MCV, fL | 92.00 (88.40, 95.30) | 91.90 (88.50, 95.30) | 92.20 (88.20, 96.10) | 0.9 |
| MCH, pg | 30.90 (29.60, 32.10) | 31.00 (29.60, 32.10) | 30.40 (29.00, 31.50) | 0.001 |
| MCHC, g dL^-1^ | 334.00 (325.00, 343.00) | 337.00 (326.00, 344.00) | 330.00 (322.00, 339.00) | <0.001 |
| Globulin, g L^-1^ | 24.65 (21.70, 27.75) | 24.40 (21.60, 26.90) | 25.60 (22.00, 29.20) | 0.001 |
| Glucose, mmol L^-1^ | 6.49 (5.30, 8.10) | 6.50 (5.32, 8.08) | 6.41 (5.22, 8.17) | 0.4 |
| Basophils count, K μL^-1^ | 0.02 (0.01, 0.03) | 0.02 (0.01, 0.03) | 0.02 (0.01, 0.03) | 0.9 |
| Albumin, g L^-1^ | 40.80 (37.60, 44.20) | 41.10 (37.80, 44.70) | 39.90 (37.00, 42.90) | 0.006 |
| Platelet, K uL^-1^ | 219.00 (172.00, 266.00) | 216.00 (172.00, 263.00) | 226.00 (178.00, 284.00) | 0.11 |
| WBC, K uL-1 | 7.79 (5.99, 10.29) | 7.93 (6.04, 10.39) | 7.33 (5.83, 9.81) | 0.2 |
| TC, mmol L^-1^ | 4.44 (3.55, 5.26) | 4.44 (3.56, 5.26) | 4.45 (3.54, 5.37) | 0.9 |
| LDL, mmol L^-1^ | 2.71 (1.96, 3.44) | 2.73 (1.96, 3.44) | 2.66 (1.91, 3.55) | >0.9 |
| Total triglycerides, mmol L^-1^ | 1.16 (0.82, 1.67) | 1.15 (0.83, 1.67) | 1.18 (0.81, 1.61) | 0.8 |
| HDL, mmol L^-1^ | 1.12 (0.93, 1.33) | 1.12 (0.93, 1.33) | 1.13 (0.91, 1.32) | 0.8 |
| ALT, U L^-1^ | 20.00 (13.00, 35.00) | 20.00 (13.00, 38.00) | 20.00 (11.00, 32.00) | 0.05 |
| AST, U L^-1^ | 25.00 (17.00, 65.00) | 26.00 (17.00, 72.00) | 25.00 (16.00, 45.00) | 0.05 |
| IDBL, μmol L^-1^ | 5.90 (3.60, 9.35) | 6.00 (3.80, 9.50) | 5.40 (3.10, 8.60) | 0.061 |
| ALP, U L^-1^ | 81.00 (64.00, 100.00) | 79.00 (62.00, 97.00) | 83.00 (68.00, 103.00) | 0.05 |
| TBI, μmol L^-1^ | 11.00 (7.30, 16.25) | 11.30 (7.60, 16.40) | 10.60 (6.80, 15.70) | 0.2 |
| TBA, μmol L^-1^ | 2.85 (1.70, 4.80) | 2.80 (1.60, 4.70) | 3.00 (1.80, 5.30) | 0.3 |
| DBIL, μmol L^-1^ | 4.80 (3.50, 6.80) | 4.90 (3.70, 6.90) | 4.80 (3.30, 6.20) | 0.11 |
| Lym, K μL^-1^ | 1.47 (1.01, 2.22) | 1.49 (1.01, 2.14) | 1.43 (1.00, 2.36) | 0.5 |
| MPV, fL | 9.90 (9.30, 10.80) | 9.90 (9.30, 10.80) | 10.00 (9.40, 10.80) | 0.7 |
| PDW, % | 11.55 (10.20, 13.20) | 11.60 (10.20, 13.20) | 11.50 (10.40, 13.20) | 0.8 |
| Medical therapy |  |  |  |  |
| ACEI/ARB, n(%) | 311 (45%) | 214 (43%) | 97 (52%) | 0.039 |
| Beta-blockers, n(%) | 300 (44%) | 205 (41%) | 95 (51%) | 0.025 |
| CCB, n(%) | 313 (46%) | 215 (43%) | 98 (52%) | 0.032 |
| Metformin, n(%) | 133 (19%) | 91 (18%) | 42 (22%) | 0.2 |
| Insulin injections, n(%) | 99 (14%) | 67 (13%) | 32 (17%) | 0.2 |
| Statins, n(%) | 108 (16%) | 73 (15%) | 35 (19%) | 0.2 |
| Diuretics, n(%) | 102 (15%) | 67 (13%) | 35 (19%) | 0.087 |

**#Continuous variables are expressed as interquartile ranges. Categorical variables are expressed as frequency (percentage). CVD, cardiovascular disease, AF, atrial fibrillation, CKD, chronic kidney disease, COPD, Chronic obstructive pulmonary disease, BNP, B-type natriuretic peptide, ESI, erythrocyte stress index, RBC, ePVS, estimated plasma volume status, red blood cell count, RDW, red blood cell distribution, MCV, mean cell volume, MCH, mean corpuscular hemoglobin, MCHC, mean corpuscular hemoglobin concentration, WBC, white blood cell count, TC, total cholesterol, LDL, low-density lipoprotein, HDL, high-density lipoprotein, ALT, alanine aminotransferase, AST, aspartate aminotransferase, IBIL, indirect bilirubin, ALP, alkaline phosphatase, TBI, total bilirubin, TBA, total bile acids, DBIL, direct bilirubin, Lym, lymphocyte count, MPV, mean platelet volume, PDW, platelet distribution width, ACEI/ARB, ACE-inhibitors/AT1-receptor antagonists, CCB, dihydropyridine Ca2+ channel blockers.**

**Table S13. The Cox regression of different lipids in RBC membrane and plasma of MACE**

| Organ | Lipid | Model 1 |  | Model 2 |  | Model 3 |  | Model 4 |  |
| --- | --- | --- | --- | --- | --- | --- | --- | --- | --- |
|  |  | HR (95% CI) | *P* value | HR (95% CI) | *P* value | HR (95% CI) | *P* value | HR (95% CI) | *P* value |
| Plasma | Cer 18:0;O2/16:0 | 0.97 (0.93, 1.01) | 0.11 | 0.97 (0.93, 1.01) | 0.11 | 0.97 (0.93, 1.01) | 0.11 | 0.98 (0.94, 1.01) | 0.2 |
|  | Cer 18:0;O2/24:1 | 0.99 (0.95, 1.03) | 0.5 | 0.99 (0.95, 1.03) | 0.5 | 0.99 (0.95, 1.02) | 0.4 | 0.99 (0.95, 1.02) | 0.5 |
|  | Cer 18:1;O2/24:1 | 1.00 (0.99, 1.01) | >0.9 | 1.00 (0.99, 1.01) | >0.9 | 1.00 (0.99, 1.02) | 0.9 | 1.00 (0.99, 1.02) | 0.8 |
|  | GlcCer 18:1;O2/12:0 | 1.03 (1.01, 1.06) | 0.055 | 1.03 (1.01, 1.07) | 0.049 | 1.03 (1.01, 1.07) | 0.036 | 1.03 (1.01, 1.07) | 0.038 |
|  | LPE 16:0 | 0.99 (0.95, 1.02) | 0.4 | 0.99 (0.95, 1.02) | 0.4 | 0.99 (0.96, 1.02) | 0.5 | 0.99 (0.96, 1.03) | 0.6 |
|  | LPE 16:1 | 0.99 (0.96, 1.01) | 0.3 | 0.99 (0.96, 1.01) | 0.3 | 0.99 (0.96, 1.02) | 0.4 | 0.99 (0.97, 1.02) | 0.5 |
|  | LPE 18:0 | 0.98 (0.96, 1.01) | 0.2 | 0.98 (0.96, 1.01) | 0.2 | 0.99 (0.96, 1.01) | 0.3 | 0.99 (0.96, 1.01) | 0.4 |
|  | LPE 18:1 | 0.98 (0.95, 1.01) | 0.2 | 0.98 (0.95, 1.01) | 0.2 | 0.98 (0.95, 1.01) | 0.2 | 0.98 (0.96, 1.01) | 0.2 |
|  | LPE 18:3 | 0.98 (0.96, 1.01) | 0.2 | 0.98 (0.96, 1.01) | 0.2 | 0.98 (0.96, 1.01) | 0.2 | 0.99 (0.96, 1.01) | 0.2 |
|  | LPE 20:0 | 0.98 (0.95, 1.01) | 0.2 | 0.98 (0.95, 1.01) | 0.2 | 0.98 (0.95, 1.01) | 0.3 | 0.98 (0.95, 1.01) | 0.3 |
|  | LPE 20:1 | 0.98 (0.95, 1.01) | 0.14 | 0.98 (0.95, 1.01) | 0.2 | 0.98 (0.95, 1.01) | 0.2 | 0.98 (0.95, 1.01) | 0.2 |
|  | LPE 20:2 | 0.98 (0.94, 1.01) | 0.15 | 0.98 (0.94, 1.01) | 0.2 | 0.98 (0.95, 1.01) | 0.2 | 0.98 (0.95, 1.01) | 0.3 |
|  | LPE 22:4 | 0.98 (0.96, 1.01) | 0.2 | 0.98 (0.96, 1.01) | 0.2 | 0.99 (0.96, 1.01) | 0.3 | 0.98 (0.96, 1.01) | 0.2 |
|  | LPE 22:5 | 0.96 (0.93, 1.00) | 0.045 | 0.96 (0.93, 1.00) | 0.047 | 0.97 (0.93, 1.00) | 0.064 | 0.97 (0.93, 1.00) | 0.070 |
|  | PE 14:0_18:1 | 0.99 (0.96, 1.01) | 0.3 | 0.99 (0.96, 1.01) | 0.3 | 0.99 (0.96, 1.02) | 0.4 | 0.99 (0.96, 1.02) | 0.6 |
|  | PE 16:0_16:0 | 1.00 (0.99, 1.02) | 0.7 | 1.00 (0.99, 1.02) | 0.7 | 1.01 (0.99, 1.02) | 0.6 | 1.00 (0.99, 1.02) | 0.6 |
|  | PE 16:0_16:1 | 1.01 (0.99, 1.02) | 0.3 | 1.01 (0.99, 1.02) | 0.3 | 1.01 (0.99, 1.02) | 0.2 | 1.01 (1.0, 1.02) | 0.2 |
|  | PE 16:0_18:0 | 1.00 (0.98, 1.02) | 0.8 | 1.00 (0.98, 1.02) | 0.9 | 1.00 (0.98, 1.02) | >0.9 | 1.00 (0.98, 1.02) | >0.9 |
|  | PE 16:0_18:1 | 1.00 (0.98, 1.01) | 0.8 | 1.00 (0.98, 1.01) | 0.9 | 1.00 (0.98, 1.01) | >0.9 | 1.00 (0.98, 1.01) | 0.9 |
|  | PE 16:0_18:2 | 0.98 (0.95, 1.01) | 0.2 | 0.98 (0.95, 1.01) | 0.2 | 0.98 (0.95, 1.01) | 0.3 | 0.99 (0.96, 1.02) | 0.4 |
|  | PE 16:0_18:3 | 0.99 (0.97, 1.01) | 0.5 | 0.99 (0.97, 1.01) | 0.5 | 0.99 (0.97, 1.01) | 0.6 | 1.00 (0.98, 1.02) | 0.7 |
|  | PE 16:0_20:2 | 0.99 (0.97, 1.02) | 0.5 | 0.99 (0.97, 1.02) | 0.5 | 0.99 (0.97, 1.02) | 0.6 | 1.00 (0.97, 1.02) | 0.7 |
|  | PE 16:0_20:3 | 1.00 (0.97, 1.02) | 0.8 | 1.00 (0.97, 1.02) | 0.8 | 1.00 (0.98, 1.02) | 0.9 | 1.00 (0.98, 1.02) | >0.9 |
|  | PE 16:0_20:4 | 0.98 (0.96, 1.01) | 0.3 | 0.99 (0.96, 1.01) | 0.3 | 0.99 (0.96, 1.02) | 0.4 | 0.99 (0.96, 1.02) | 0.5 |
|  | PE 16:0_22:4 | 0.99 (0.98, 1.01) | 0.3 | 0.99 (0.98, 1.01) | 0.3 | 0.99 (0.98, 1.01) | 0.4 | 0.99 (0.98, 1.01) | 0.3 |
|  | PE 16:0_22:5 | 0.98 (0.95, 1.00) | 0.11 | 0.98 (0.95, 1.01) | 0.11 | 0.98 (0.95, 1.01) | 0.12 | 0.98 (0.95, 1.01) | 0.13 |
|  | PE 16:1_18:1 | 0.99 (0.97, 1.01) | 0.6 | 1.0 (0.97, 1.01) | 0.6 | 1.00 (0.98, 1.02) | 0.7 | 1.00 (0.98, 1.02) | 0.7 |
|  | PE 18:0_22:4 | 0.97 (0.94, 1.01) | 0.10 | 0.97 (0.94, 1.01) | 0.11 | 0.97 (0.94, 1.01) | 0.13 | 0.97 (0.94, 1.01) | 0.10 |
|  | PE 18:1_18:1 | 0.98 (0.96, 1.01) | 0.13 | 0.98 (0.96, 1.01) | 0.13 | 0.98 (0.96, 1.01) | 0.2 | 0.98 (0.96, 1.01) | 0.15 |
|  | PE 18:2_18:2 | 0.99 (0.97, 1.01) | 0.5 | 0.99 (0.97, 1.01) | 0.5 | 0.99 (0.97, 1.01) | 0.6 | 1.00 (0.98, 1.02) | 0.7 |
|  | PE O-16:0/22:4 | 0.97 (0.94, 1.00) | 0.052 | 0.97 (0.94, 1.00) | 0.054 | 0.97 (0.94, 1.00) | 0.072 | 0.97 (0.93, 1.00) | 0.057 |
|  | PE O-18:0/18:1 | 0.97 (0.94, 1.00) | 0.091 | 0.97 (0.94, 1.01) | 0.10 | 0.97 (0.94, 1.01) | 0.11 | 0.98 (0.95, 1.01) | 0.2 |
|  | PE O-18:0/22:4 | 0.99 (0.97, 1.01) | 0.3 | 0.99 (0.97, 1.01) | 0.3 | 0.99 (0.97, 1.01) | 0.4 | 0.99 (0.97, 1.01) | 0.3 |
|  | PE P-16:0/16:0 | 0.97 (0.93, 1.02) | 0.2 | 0.97 (0.93, 1.01) | 0.2 | 0.97 (0.93, 1.02) | 0.2 | 0.98 (0.94, 1.02) | 0.3 |
|  | PE P-16:0/18:1 | 0.98 (0.94, 1.01) | 0.2 | 0.98 (0.94, 1.01) | 0.2 | 0.98 (0.95, 1.01) | 0.2 | 0.98 (0.95, 1.01) | 0.2 |
|  | PE P-16:0/22:4 | 0.98 (0.96, 1.01) | 0.2 | 0.98 (0.96, 1.01) | 0.2 | 0.98 (0.96, 1.01) | 0.2 | 0.98 (0.96, 1.01) | 0.2 |
|  | PE P-18:0/16:1 | 0.97 (0.93, 1.01) | 0.10 | 0.97 (0.93, 1.01) | 0.11 | 0.97 (0.93, 1.01) | 0.2 | 0.97 (0.93, 1.01) | 0.2 |
|  | PE P-18:0/18:1 | 0.99 (0.96, 1.01) | 0.3 | 0.99 (0.96, 1.01) | 0.3 | 0.99 (0.96, 1.02) | 0.4 | 0.99 (0.96, 1.02) | 0.5 |
|  | PE P-18:0/20:2 | 0.98 (0.95, 1.01) | 0.11 | 0.98 (0.95, 1.01) | 0.12 | 0.98 (0.95, 1.01) | 0.2 | 0.98 (0.95, 1.01) | 0.2 |
|  | PE P-18:0/22:4 | 0.99 (0.98, 1.01) | 0.3 | 0.99 (0.98, 1.01) | 0.3 | 0.99 (0.98, 1.01) | 0.4 | 0.99 (0.98, 1.01) | 0.3 |
|  | PE P-18:0/22:5 | 0.97 (0.94, 1.00) | 0.041 | 0.97 (0.94, 1.00) | 0.043 | 0.97 (0.94, 1.00) | 0.048 | 0.96 (0.93, 1.00) | 0.051 |
|  | PE P-18:1/16:0 | 0.98 (0.95, 1.02) | 0.3 | 0.98 (0.95, 1.02) | 0.3 | 0.98 (0.95, 1.02) | 0.3 | 0.98 (0.95, 1.02) | 0.3 |
|  | PE P-18:1/22:4 | 0.98 (0.96, 1.00) | 0.086 | 0.98 (0.96, 1.00) | 0.089 | 0.98 (0.96, 1.00) | 0.080 | 0.98 (0.96, 1.00) | 0.095 |
| RBC membrane | Cer 18:0;O2/16:0 | 1.05 (1.02, 1.08) | 0.001 | 1.05 (1.02, 1.08) | 0.001 | 1.04 (1.01, 1.07) | 0.004 | 1.04 (1.01, 1.07) | 0.003 |
|  | GlcCer 18:0;O2/18:0 | 1.03 (0.99, 1.08) | 0.14 | 1.03 (0.99, 1.08) | 0.2 | 1.03 (0.98, 1.07) | 0.2 | 1.04 (1.0, 1.09) | 0.084 |
|  | GlcCer 18:1;O2/14:0 | 0.99 (0.96, 1.02) | 0.5 | 0.99 (0.96, 1.02) | 0.5 | 1.00 (0.96, 1.03) | 0.8 | 0.99 (0.96, 1.03) | 0.6 |
|  | GlcCer 18:1;O2/16:0 | 1.00 (0.96, 1.04) | >0.9 | 1.00 (0.96, 1.04) | >0.9 | 0.99 (0.95, 1.03) | 0.6 | 0.99 (0.96, 1.03) | 0.8 |
|  | LPE 16:0 | 1.00 (0.98, 1.02) | 0.9 | 1.00 (0.98, 1.02) | 0.9 | 1.00 (0.98, 1.02) | >0.9 | 1.00 (0.97, 1.02) | 0.7 |
|  | LPE 16:1 | 1.00 (0.98, 1.01) | 0.9 | 1.00 (0.98, 1.01) | 0.7 | 1.00 (0.98, 1.01) | 0.7 | 0.99 (0.98, 1.01) | 0.3 |
|  | LPE 18:0 | 1.05 (1.00, 1.09) | 0.033 | 1.04 (1.00, 1.09) | 0.039 | 1.05 (1.01, 1.09) | 0.026 | 1.06 (1.01, 1.10) | 0.011 |
|  | LPE 18:1 | 1.00 (0.98, 1.02) | >0.9 | 1.00 (0.98, 1.02) | >0.9 | 1.00 (0.98, 1.02) | 0.9 | 0.99 (0.97, 1.01) | 0.5 |
|  | LPE 20:0 | 1.00 (0.98, 1.02) | 0.8 | 1.00 (0.98, 1.02) | 0.9 | 1.00 (0.98, 1.02) | 0.9 | 0.99 (0.97, 1.02) | 0.5 |
|  | LPE 20:1 | 1.00 (0.99, 1.02) | 0.6 | 1.01 (0.99, 1.02) | 0.6 | 1.01 (0.99, 1.02) | 0.6 | 1.00 (0.98, 1.02) | >0.9 |
|  | LPE 20:2 | 0.99 (0.96, 1.01) | 0.4 | 0.99 (0.96, 1.01) | 0.3 | 0.99 (0.96, 1.01) | 0.3 | 0.98 (0.96, 1.01) | 0.2 |
|  | PE 18:1_22:4 | 1.03 (0.99, 1.07) | 0.2 | 1.03 (0.99, 1.07) | 0.2 | 1.02 (0.99, 1.07) | 0.2 | 1.03 (0.99, 1.07) | 0.14 |
|  | PE 18:2_18:2 | 1.01 (0.97, 1.05) | 0.7 | 1.01 (0.97, 1.05) | 0.7 | 1.01 (0.97, 1.06) | 0.5 | 1.03 (0.98, 1.07) | 0.3 |
|  | PE 18:2_18:3 | 0.99 (0.95, 1.03) | 0.6 | 0.99 (0.95, 1.03) | 0.6 | 0.99 (0.95, 1.03) | 0.6 | 1.00 (0.96, 1.04) | >0.9 |
|  | PE 18:2_20:3 | 1.00 (0.96, 1.04) | >0.9 | 1.00 (0.96, 1.04) | >0.9 | 1.00 (0.96, 1.04) | >0.9 | 1.01 (0.97, 1.05) | 0.8 |
|  | PE 18:2_20:4 | 0.99 (0.96, 1.01) | 0.3 | 0.98 (0.96, 1.01) | 0.3 | 0.99 (0.96, 1.02) | 0.3 | 0.99 (0.96, 1.02) | 0.6 |
|  | PE 18:2_22:4 | 1.01 (0.98, 1.05) | 0.5 | 1.02 (0.98, 1.06) | 0.4 | 1.01 (0.98, 1.05) | 0.5 | 1.02 (0.98, 1.06) | 0.4 |
|  | PE O-16:0/20:4 | 1.05 (1.00, 1.11) | 0.066 | 1.05 (0.99, 1.11) | 0.090 | 1.04 (0.99, 1.10) | 0.13 | 1.05 (1.00, 1.11) | 0.062 |
|  | PE P-16:0/16:1 | 1.03 (0.99, 1.08) | 0.11 | 1.03 (0.99, 1.07) | 0.2 | 1.02 (0.98, 1.07) | 0.3 | 1.01 (0.97, 1.05) | 0.7 |
|  | PE P-16:0/20:4 | 1.02 (0.97, 1.08) | 0.4 | 1.02 (0.97, 1.08) | 0.4 | 1.02 (0.97, 1.07) | 0.5 | 1.02 (0.97, 1.08) | 0.4 |
|  | PE P-16:0/22:4 | 1.04 (0.99, 1.10) | 0.10 | 1.04 (0.99, 1.10) | 0.095 | 1.04 (0.99, 1.10) | 0.088 | 1.05 (1.00, 1.11) | 0.051 |
|  | PE P-18:0/22:4 | 1.04 (0.99, 1.10) | 0.11 | 1.05 (0.99, 1.10) | 0.088 | 1.05 (1.00, 1.10) | 0.077 | 1.06 (1.00, 1.11) | 0.052 |
|  | PE P-18:1/18:2 | 1.01 (0.96, 1.06) | 0.7 | 1.01 (0.96, 1.06) | 0.7 | 1.00 (0.96, 1.05) | 0.9 | 1.01 (0.96, 1.06) | 0.7 |
|  | PE P-18:1/20:4 | 1.03 (0.98, 1.07) | 0.3 | 1.02 (0.98, 1.07) | 0.3 | 1.02 (0.98, 1.06) | 0.4 | 1.03 (0.99, 1.07) | 0.2 |
|  | PE P-18:1/22:4 | 1.01 (0.98, 1.04) | 0.6 | 1.01 (0.98, 1.04) | 0.6 | 1.00 (0.98, 1.03) | 0.8 | 1.00 (0.97, 1.03) | >0.9 |
|  | PE P-18:2/18:2 | 0.99 (0.95, 1.04) | 0.8 | 1.00 (0.95, 1.04) | 0.9 | 1.00 (0.95, 1.04) | 0.9 | 1.01 (0.96, 1.05) | 0.8 |
|  | PE P-18:2/20:4 | 1.00 (0.96, 1.05) | 0.8 | 1.00 (0.96, 1.05) | 0.8 | 1.01 (0.97, 1.05) | 0.7 | 1.02 (0.98, 1.06) | 0.4 |

**#The Cox regression was adjusted with four models, the adjusted details of the four models included Model 1: no adjustment; Model 2: age + sex; Model 3: Model 2 + CVD + COPD + Anemia; Model 4: Model 3 + BNP + Globulin + Albumin + WBC + ACEI/ARB + Beta-blockers + CCB.**

**Table S14. The parameters of all lipid isoforms**

| Lipid | Precursor Ion (*m/z*) | Product Ion (*m/z*) | Retention Time (min) | Collision Energy (v) | Adduct |
| --- | --- | --- | --- | --- | --- |
| Cer 18:0;O2/16:0 | 540.6 | 266.4 | 6.15 | 43 | [M+H]^+^ |
| Cer 18:0;O2/18:0 | 568.7 | 266.4 | 6.45 | 43 | [M+H]^+^ |
| Cer 18:0;O2/20:0 | 596.7 | 266.4 | 6.8 | 43 | [M+H]^+^ |
| Cer 18:0;O2/22:0 | 624.8 | 266.4 | 7.14 | 43 | [M+H]^+^ |
| Cer 18:0;O2/24:0 | 652.9 | 266.4 | 7.48 | 43 | [M+H]^+^ |
| Cer 18:0;O2/24:1 | 650.9 | 266.4 | 7.19 | 43 | [M+H]^+^ |
| Cer 18:0;O2/26:0 | 680.5 | 266.4 | 7.75 | 43 | [M+H]^+^ |
| Cer 18:0;O2/26:1 | 678.5 | 266.4 | 7.38 | 43 | [M+H]^+^ |
| Cer 18:1;O2/22:0 | 622.7 | 264.4 | 7.33 | 43 | [M+H]^+^ |
| Cer 18:1;O2/22:1 | 620.7 | 264.4 | 7.44 | 43 | [M+H]^+^ |
| Cer 18:1;O2/24:1 | 648.8 | 264.4 | 7.65 | 43 | [M+H]^+^ |
| DG 14:0_18:1 | 584.4 | 285.2 | 6.2 | 26 | [M+NH_4_]^+^ |
| DG 16:0_16:0 | 586.5 | 313.3 | 6.44 | 26 | [M+NH_4_]^+^ |
| DG 16:0_18:1 | 612.6 | 313.2 | 6.54 | 26 | [M+NH_4_]^+^ |
| DG 16:0_18:2 | 610.4 | 313.2 | 6.29 | 26 | [M+NH_4_]^+^ |
| DG 18:1_18:1 | 638.4 | 339.3 | 6.63 | 26 | [M+NH_4_]^+^ |
| DG 18:1_18:2 | 636.5 | 339.3 | 6.39 | 26 | [M+NH_4_]^+^ |
| DG 18:1_20:4 | 660.5 | 339.3 | 6.39 | 26 | [M+NH_4_]^+^ |
| DG 18:2_20:4 | 658.5 | 337.3 | 6.14 | 26 | [M+NH_4_]^+^ |
| FA 14:0 | 227.1 | 227.1 | 3.35 | -10 | [M-H]^-^ |
| FA 14:1 | 225.1 | 225.1 | 3 | -10 | [M-H]^-^ |
| FA 16:0 | 255.1 | 255.1 | 3.81 | -10 | [M-H]^-^ |
| FA 16:1 | 253.1 | 253.1 | 3.51 | -10 | [M-H]^-^ |
| FA 16:2 | 251.1 | 251.1 | 3.22 | -10 | [M-H]^-^ |
| FA 18:0 | 283.2 | 283.2 | 4.2 | -10 | [M-H]^-^ |
| FA 18:1 | 281.2 | 281.2 | 3.93 | -10 | [M-H]^-^ |
| FA 18:2 | 279.2 | 279.2 | 3.65 | -10 | [M-H]^-^ |
| FA 18:3 | 277.2 | 277.2 | 3.38 | -10 | [M-H]^-^ |
| FA 20:0 | 311.2 | 311.2 | 4.53 | -10 | [M-H]^-^ |
| FA 20:1 | 309.2 | 309.2 | 4.27 | -10 | [M-H]^-^ |
| FA 20:2 | 307.2 | 307.2 | 4.02 | -10 | [M-H]^-^ |
| FA 20:3 | 305.2 | 305.2 | 3.81 | -10 | [M-H]^-^ |
| FA 20:4 | 303.2 | 303.2 | 3.63 | -10 | [M-H]^-^ |
| FA 20:5 | 301.2 | 301.2 | 3.36 | -10 | [M-H]^-^ |
| FA 22:4 | 331.3 | 331.3 | 6.21 | -10 | [M-H]^-^ |
| FA 22:5 | 329.2 | 329.2 | 5.99 | -10 | [M-H]^-^ |
| FA 22:6 | 327.2 | 327.2 | 5.67 | -10 | [M-H]^-^ |
| FA 24:0 | 367.3 | 367.3 | 5.18 | -10 | [M-H]^-^ |
| FA 24:1 | 365.3 | 365.3 | 4.87 | -10 | [M-H]^-^ |
| GlcCer 18:0;O2/18:0 | 730.7 | 266.4 | 5.89 | 43 | [M+H]^+^ |
| GlcCer 18:1;O2/12:0 | 644.5 | 264.4 | 7.24 | 43 | [M+H]^+^ |
| GlcCer 18:1;O2/14:0 | 672.5 | 264.4 | 5.6 | 43 | [M+H]^+^ |
| GlcCer 18:1;O2/16:0 | 700.7 | 264.4 | 5.9 | 43 | [M+H]^+^ |
| GlcCer 18:1;O2/18:0 | 728.8 | 264.4 | 6.14 | 43 | [M+H]^+^ |
| GlcCer 18:1;O2/18:1 | 726.7 | 264.4 | 5.84 | 43 | [M+H]^+^ |
| GlcCer 18:1;O2/22:0 | 784.9 | 264.4 | 6.7 | 43 | [M+H]^+^ |
| GlcCer 18:1;O2/24:0 | 812.9 | 264.4 | 7.12 | 43 | [M+H]^+^ |
| GlcCer 18:1;O2/24:1 | 810.9 | 264.4 | 6.8 | 43 | [M+H]^+^ |
| LacCer 18:1;O2/14:0 | 834.9 | 264.4 | 5.24 | 43 | [M+H]^+^ |
| LacCer 18:1;O2/16:0 | 862.9 | 264.4 | 5.51 | 43 | [M+H]^+^ |
| LPA 16:0 | 409.236 | 255.233 | 3.45 | -50 | [M-H]^-^ |
| LPC 14:0 | 526.317 | 227.202 | 3.3 | -43 | [M+OAc]^-^ |
| LPC 16:0 | 554.346 | 255.233 | 3.7 | -50 | [M+OAc]^-^ |
| LPC 16:1 | 552.331 | 253.217 | 3.5 | -50 | [M+OAc]^-^ |
| LPC 18:0 | 582.378 | 283.264 | 4.1 | -50 | [M+OAc]^-^ |
| LPC 18:1 | 580.362 | 281.249 | 3.9 | -50 | [M+OAc]^-^ |
| LPC 18:2 | 578.346 | 279.233 | 3.6 | -50 | [M+OAc]^-^ |
| LPC 20:4 | 602.346 | 303.233 | 3.5 | -50 | [M+OAc]^-^ |
| LPE 16:0 | 452.278 | 255.233 | 3.81 | -40 | [M-H]^-^ |
| LPE 16:1 | 450.263 | 253.217 | 3.51 | -40 | [M-H]^-^ |
| LPE 18:0 | 480.31 | 283.264 | 4.21 | -40 | [M-H]^-^ |
| LPE 18:1 | 478.293 | 281.249 | 3.94 | -40 | [M-H]^-^ |
| LPE 18:2 | 476.278 | 279.233 | 3.65 | -40 | [M-H]^-^ |
| LPE 18:3 | 474.263 | 277.217 | 3.39 | -40 | [M-H]^-^ |
| LPE 20:0 | 508.341 | 311.3 | 4.55 | -40 | [M-H]^-^ |
| LPE 20:1 | 506.325 | 309.28 | 4.28 | -40 | [M-H]^-^ |
| LPE 20:2 | 504.31 | 307.264 | 4.04 | -40 | [M-H]^-^ |
| LPE 20:3 | 502.294 | 305.249 | 3.81 | -40 | [M-H]^-^ |
| LPE 20:4 | 500.278 | 303.233 | 3.65 | -40 | [M-H]^-^ |
| LPE 22:4 | 528.31 | 331.264 | 3.97 | -40 | [M-H]^-^ |
| LPE 22:5 | 526.294 | 329.249 | 3.75 | -40 | [M-H]^-^ |
| LPE 22:6 | 524.278 | 327.233 | 3.6 | -40 | [M-H]^-^ |
| LPI 16:0 | 571.289 | 255.233 | 3.28 | -50 | [M-H]^-^ |
| LPI 16:1 | 569.273 | 253.217 | 2.96 | -50 | [M-H]^-^ |
| LPI 18:0 | 599.32 | 283.264 | 3.68 | -50 | [M-H]^-^ |
| LPI 18:1 | 597.305 | 281.249 | 3.41 | -50 | [M-H]^-^ |
| LPI 18:2 | 595.289 | 279.233 | 3.12 | -50 | [M-H]^-^ |
| LPI 20:3 | 621.305 | 305.249 | 3.29 | -50 | [M-H]^-^ |
| LPI 20:4 | 619.289 | 303.233 | 3.12 | -50 | [M-H]^-^ |
| PA 14:0_18:1 | 645.45 | 281.249 | 3.87 | -50 | [M-H]^-^ |
| PA 16:0_16:0 | 647.466 | 255.233 | 5.18 | -50 | [M-H]^-^ |
| PA 16:0_18:0 | 675.497 | 283.264 | 5.34 | -50 | [M-H]^-^ |
| PA 16:0_18:1 | 673.481 | 281.249 | 5.15 | -50 | [M-H]^-^ |
| PA 16:0_18:2 | 671.466 | 279.233 | 5.85 | -50 | [M-H]^-^ |
| PA 18:0_20:4 | 723.497 | 303.233 | 5.87 | -50 | [M-H]^-^ |
| PA 18:0_22:6 | 747.497 | 327.233 | 5.58 | -50 | [M-H]^-^ |
| PA 18:1_18:1 | 699.497 | 281.249 | 5.85 | -50 | [M-H]^-^ |
| PA 18:1_20:2 | 725.513 | 307.264 | 5.75 | -50 | [M-H]^-^ |
| PA 20:0_20:4 | 751.528 | 303.233 | 5.95 | -50 | [M-H]^-^ |
| PC 14:0_18:1 | 790.56 | 281.249 | 5.56 | -50 | [M+OAc]^-^ |
| PC 14:0_18:2 | 788.545 | 279.233 | 5.34 | -50 | [M+OAc]^-^ |
| PC 14:0_20:4 | 812.545 | 303.233 | 5.25 | -50 | [M+OAc]^-^ |
| PC 16:0_16:0 | 792.576 | 255.233 | 5.75 | -50 | [M+OAc]^-^ |
| PC 16:0_18:0 | 820.607 | 283.264 | 6.05 | -50 | [M+OAc]^-^ |
| PC 16:0_18:1 | 818.592 | 281.249 | 5.85 | -50 | [M+OAc]^-^ |
| PC 16:0_18:2 | 816.576 | 279.233 | 5.63 | -50 | [M+OAc]^-^ |
| PC 16:1_18:1 | 816.576 | 281.249 | 5.66 | -50 | [M+OAc]^-^ |
| PC 16:1_18:2 | 814.56 | 279.233 | 5.44 | -50 | [M+OAc]^-^ |
| PE 14:0_16:0 | 662.477 | 255.233 | 5.27 | -50 | [M-H]^-^ |
| PE 14:0_18:1 | 688.492 | 281.249 | 5.68 | -43 | [M-H]^-^ |
| PE 14:0_18:2 | 686.477 | 279.233 | 5.48 | -50 | [M-H]^-^ |
| PE 16:0_16:0 | 690.508 | 255.233 | 5.86 | -50 | [M-H]^-^ |
| PE 16:0_16:1 | 688.492 | 253.217 | 5.7 | -50 | [M-H]^-^ |
| PE 16:0_18:0 | 718.539 | 283.264 | 6.16 | -50 | [M-H]^-^ |
| PE 16:0_18:1 | 716.524 | 281.249 | 5.99 | -50 | [M-H]^-^ |
| PE 16:0_18:2 | 714.508 | 279.233 | 5.77 | -50 | [M-H]^-^ |
| PE 16:0_18:3 | 712.492 | 277.217 | 5.58 | -50 | [M-H]^-^ |
| PE 16:0_20:1 | 744.555 | 309.28 | 6.28 | -50 | [M-H]^-^ |
| PE 16:0_20:2 | 742.539 | 307.264 | 6.03 | -50 | [M-H]^-^ |
| PE 16:0_20:3 | 740.524 | 305.249 | 5.89 | -50 | [M-H]^-^ |
| PE 16:0_20:4 | 738.508 | 303.233 | 5.7 | -50 | [M-H]^-^ |
| PE 16:0_20:5 | 736.492 | 301.217 | 5.5 | -50 | [M-H]^-^ |
| PE 16:0_22:4 | 766.539 | 331.264 | 6.02 | -50 | [M-H]^-^ |
| PE 16:0_22:5 | 764.524 | 329.249 | 5.8 | -50 | [M-H]^-^ |
| PE 16:0_22:6 | 762.508 | 327.233 | 5.73 | -50 | [M-H]^-^ |
| PE 16:1_18:1 | 714.508 | 281.249 | 5.7 | -50 | [M-H]^-^ |
| PE 16:1_18:2 | 712.492 | 279.233 | 5.5 | -50 | [M-H]^-^ |
| PE 18:0_18:0 | 746.57 | 283.264 | 6.5 | -50 | [M-H]^-^ |
| PE 18:0_18:1 | 744.555 | 281.249 | 6.28 | -50 | [M-H]^-^ |
| PE 18:0_18:2 | 742.539 | 279.233 | 6.05 | -50 | [M-H]^-^ |
| PE 18:0_18:3 | 740.524 | 277.217 | 5.86 | -50 | [M-H]^-^ |
| PE 18:0_20:1 | 772.586 | 309.28 | 6.56 | -50 | [M-H]^-^ |
| PE 18:0_20:2 | 770.57 | 307.264 | 6.3 | -50 | [M-H]^-^ |
| PE 18:0_20:3 | 768.555 | 305.249 | 6.17 | -50 | [M-H]^-^ |
| PE 18:0_20:4 | 766.539 | 303.233 | 6.08 | -50 | [M-H]^-^ |
| PE 18:0_20:5 | 764.524 | 301.217 | 5.87 | -50 | [M-H]^-^ |
| PE 18:0_22:4 | 794.57 | 331.264 | 6.29 | -50 | [M-H]^-^ |
| PE 18:0_22:6 | 790.539 | 327.233 | 6 | -50 | [M-H]^-^ |
| PE 18:1_18:1 | 742.539 | 281.249 | 6.08 | -50 | [M-H]^-^ |
| PE 18:1_18:2 | 740.524 | 279.233 | 5.87 | -50 | [M-H]^-^ |
| PE 18:1_18:3 | 738.508 | 277.217 | 5.67 | -50 | [M-H]^-^ |
| PE 18:1_20:1 | 770.57 | 309.28 | 6.37 | -50 | [M-H]^-^ |
| PE 18:1_20:2 | 768.555 | 307.264 | 6.14 | -50 | [M-H]^-^ |
| PE 18:1_20:3 | 766.539 | 305.249 | 5.97 | -50 | [M-H]^-^ |
| PE 18:1_20:4 | 764.524 | 303.233 | 5.88 | -50 | [M-H]^-^ |
| PE 18:1_22:4 | 792.555 | 331.264 | 6.09 | -50 | [M-H]^-^ |
| PE 18:2_18:2 | 738.508 | 279.233 | 5.64 | -50 | [M-H]^-^ |
| PE 18:2_18:3 | 736.492 | 277.217 | 5.43 | -50 | [M-H]^-^ |
| PE 18:2_20:1 | 768.555 | 309.28 | 6.15 | -50 | [M-H]^-^ |
| PE 18:2_20:2 | 766.539 | 307.264 | 5.91 | -50 | [M-H]^-^ |
| PE 18:2_20:3 | 764.524 | 305.249 | 5.74 | -50 | [M-H]^-^ |
| PE 18:2_20:4 | 762.508 | 303.233 | 5.57 | -50 | [M-H]^-^ |
| PE 18:2_22:4 | 790.539 | 331.264 | 5.89 | -50 | [M-H]^-^ |
| PE O-18:0/16:0 | 704.56 | 255.233 | 6.42 | -50 | [M-H]^-^ |
| PE O-16:0/18:1 | 702.544 | 281.249 | 6.23 | -50 | [M-H]^-^ |
| PE O-16:0/18:2 | 700.529 | 279.233 | 6.01 | -50 | [M-H]^-^ |
| PE O-16:0/20:1 | 730.576 | 309.28 | 6.52 | -50 | [M-H]^-^ |
| PE O-16:0/20:2 | 728.56 | 307.264 | 6.27 | -50 | [M-H]^-^ |
| PE O-16:0/20:3 | 726.544 | 305.249 | 6.05 | -50 | [M-H]^-^ |
| PE O-16:0/20:4 | 724.529 | 303.233 | 5.98 | -50 | [M-H]^-^ |
| PE O-16:0/22:4 | 752.56 | 331.264 | 6.2 | -50 | [M-H]^-^ |
| PE O-16:0/22:5 | 750.544 | 329.249 | 5.98 | -50 | [M-H]^-^ |
| PE O-16:0/22:6 | 748.529 | 327.233 | 5.9 | -50 | [M-H]^-^ |
| PE O-18:0/16:1 | 702.544 | 253.217 | 6.2 | -50 | [M-H]^-^ |
| PE O-18:0/18:0 | 732.591 | 283.264 | 6.7 | -50 | [M-H]^-^ |
| PE O-18:0/18:1 | 730.576 | 281.249 | 6.53 | -50 | [M-H]^-^ |
| PE O-18:0/18:2 | 728.56 | 279.233 | 6.3 | -50 | [M-H]^-^ |
| PE O-18:0/20:1 | 758.607 | 309.28 | 6.8 | -50 | [M-H]^-^ |
| PE O-18:0/20:2 | 756.591 | 307.264 | 6.6 | -50 | [M-H]^-^ |
| PE O-18:0/20:3 | 754.576 | 305.249 | 6.4 | -50 | [M-H]^-^ |
| PE O-18:0/20:4 | 752.56 | 303.233 | 6.2 | -50 | [M-H]^-^ |
| PE O-18:0/20:5 | 750.544 | 301.217 | 5.98 | -50 | [M-H]^-^ |
| PE O-18:0/22:4 | 780.591 | 331.264 | 6.54 | -50 | [M-H]^-^ |
| PE O-18:0/22:6 | 776.56 | 327.233 | 6.23 | -50 | [M-H]^-^ |
| PE P-16:0/16:0 | 674.5 | 255.233 | 6.1 | -50 | [M-H]^-^ |
| PE P-16:0/16:1 | 672.5 | 253.217 | 5.9 | -50 | [M-H]^-^ |
| PE P-16:0/18:0 | 702.5 | 283.264 | 6.4 | -50 | [M-H]^-^ |
| PE P-18:0/16:0 | 702.5 | 255.233 | 6.2 | -50 | [M-H]^-^ |
| PE P-16:0/18:1 | 700.5 | 281.249 | 6.2 | -50 | [M-H]^-^ |
| PE P-16:0/18:2 | 698.5 | 279.233 | 5.97 | -50 | [M-H]^-^ |
| PE P-16:0/18:3 | 696.5 | 277.217 | 5.76 | -50 | [M-H]^-^ |
| PE P-16:0/20:1 | 728.6 | 309.28 | 6.5 | -50 | [M-H]^-^ |
| PE P-16:0/20:2 | 726.5 | 307.264 | 6.24 | -50 | [M-H]^-^ |
| PE P-16:0/20:3 | 724.5 | 305.249 | 6.06 | -50 | [M-H]^-^ |
| PE P-16:0/20:4 | 722.5 | 303.233 | 5.98 | -50 | [M-H]^-^ |
| PE P-16:0/20:5 | 720.5 | 301.217 | 5.77 | -50 | [M-H]^-^ |
| PE P-16:0/22:4 | 750.5 | 331.264 | 6.3 | -50 | [M-H]^-^ |
| PE P-16:0/22:5 | 748.5 | 329.249 | 6.1 | -50 | [M-H]^-^ |
| PE P-16:0/22:6 | 746.5 | 327.233 | 5.9 | -50 | [M-H]^-^ |
| PE P-18:0/16:1 | 700.5 | 253.217 | 6.2 | -50 | [M-H]^-^ |
| PE P-18:0/18:0 | 730.6 | 283.264 | 6.7 | -50 | [M-H]^-^ |
| PE P-18:0/18:1 | 728.6 | 281.249 | 6.53 | -50 | [M-H]^-^ |
| PE P-18:0/18:2 | 726.5 | 279.233 | 6.29 | -50 | [M-H]^-^ |
| PE P-18:0/20:1 | 756.6 | 309.28 | 6.8 | -50 | [M-H]^-^ |
| PE P-18:0/20:2 | 754.6 | 307.264 | 6.6 | -50 | [M-H]^-^ |
| PE P-18:0/20:3 | 752.6 | 305.249 | 6.41 | -50 | [M-H]^-^ |
| PE P-18:0/20:4 | 750.5 | 303.233 | 6.31 | -50 | [M-H]^-^ |
| PE P-18:0/20:5 | 748.5 | 301.217 | 6.09 | -50 | [M-H]^-^ |
| PE P-18:0/22:4 | 778.6 | 331.264 | 6.53 | -50 | [M-H]^-^ |
| PE P-18:0/22:5 | 776.6 | 329.249 | 6.3 | -50 | [M-H]^-^ |
| PE P-18:0/22:6 | 774.5 | 327.233 | 6.22 | -50 | [M-H]^-^ |
| PE P-18:1/16:0 | 700.5 | 255.233 | 6.17 | -50 | [M-H]^-^ |
| PE P-18:1/18:0 | 728.6 | 283.2 | 6.5 | -50 | [M-H]^-^ |
| PE P-18:1/18:1 | 726.5 | 281.249 | 6.27 | -50 | [M-H]^-^ |
| PE P-18:1/18:2 | 724.5 | 279.233 | 6.03 | -50 | [M-H]^-^ |
| PE P-18:1/20:1 | 754.6 | 309.28 | 6.57 | -50 | [M-H]^-^ |
| PE P-18:1/20:2 | 752.6 | 307.264 | 6.33 | -50 | [M-H]^-^ |
| PE P-18:1/20:3 | 750.5 | 305.249 | 6.17 | -50 | [M-H]^-^ |
| PE P-18:1/20:4 | 748.5 | 303.233 | 6.04 | -50 | [M-H]^-^ |
| PE P-18:1/22:4 | 776.6 | 331.264 | 6.27 | -50 | [M-H]^-^ |
| PE P-18:1/22:5 | 774.5 | 329.249 | 6.17 | -50 | [M-H]^-^ |
| PE P-18:1/22:6 | 772.5 | 327.233 | 5.97 | -50 | [M-H]^-^ |
| PE P-18:2/18:2 | 722.5 | 279.233 | 5.82 | -50 | [M-H]^-^ |
| PE P-18:2/20:4 | 746.5 | 303.233 | 5.82 | -50 | [M-H]^-^ |
| PG 14:0_18:1 | 719.487 | 281.249 | 5.74 | -50 | [M-H]^-^ |
| PG 14:0_18:2 | 717.471 | 279.233 | 5.61 | -50 | [M-H]^-^ |
| PG 16:0_16:0 | 721.503 | 255.233 | 5.87 | -50 | [M-H]^-^ |
| PG 16:0_18:0 | 749.534 | 283.264 | 6.07 | -50 | [M-H]^-^ |
| PG 16:0_18:1 | 747.518 | 281.249 | 5.94 | -50 | [M-H]^-^ |
| PG 16:0_18:2 | 745.503 | 279.233 | 5.77 | -50 | [M-H]^-^ |
| PG 16:1_18:1 | 745.503 | 281.249 | 5.77 | -50 | [M-H]^-^ |
| PG 16:1_18:2 | 743.487 | 279.233 | 5.64 | -50 | [M-H]^-^ |
| PG 18:0_18:2 | 773.534 | 279.233 | 5.99 | -50 | [M-H]^-^ |
| PG 18:1_18:1 | 773.534 | 281.249 | 5.99 | -50 | [M-H]^-^ |
| PG 18:1_18:2 | 771.518 | 279.233 | 5.87 | -50 | [M-H]^-^ |
| PG 18:1_20:2 | 799.549 | 307.264 | 6.08 | -50 | [M-H]^-^ |
| PG 18:1_20:3 | 797.534 | 305.249 | 5.98 | -50 | [M-H]^-^ |
| PG 18:1_20:4 | 795.518 | 303.233 | 5.85 | -50 | [M-H]^-^ |
| PG 18:2_18:2 | 769.503 | 279.233 | 5.79 | -50 | [M-H]^-^ |
| PG 18:2_20:3 | 795.518 | 305.249 | 5.85 | -50 | [M-H]^-^ |
| PG 18:2_20:4 | 793.503 | 303.233 | 5.71 | -50 | [M-H]^-^ |
| PI 16:0_16:0 | 809.518 | 255.233 | 5.05 | -50 | [M-H]^-^ |
| PI 16:0_16:1 | 807.503 | 253.217 | 4.85 | -50 | [M-H]^-^ |
| PI 16:0_18:0 | 837.55 | 283.264 | 5.35 | -50 | [M-H]^-^ |
| PI 16:0_18:1 | 835.534 | 281.249 | 5.15 | -50 | [M-H]^-^ |
| PI 16:0_18:2 | 833.518 | 279.233 | 4.95 | -50 | [M-H]^-^ |
| PI 16:0_20:3 | 859.534 | 305.249 | 5.05 | -50 | [M-H]^-^ |
| PI 16:0_20:4 | 857.518 | 303.233 | 4.85 | -50 | [M-H]^-^ |
| PI 18:0_16:1 | 835.534 | 253.217 | 5.15 | -50 | [M-H]^-^ |
| PI 18:0_18:2 | 861.55 | 279.233 | 5.23 | -50 | [M-H]^-^ |
| PI 18:0_20:4 | 885.55 | 303.233 | 5.1 | -50 | [M-H]^-^ |
| PI 18:0_22:6 | 909.55 | 327.233 | 5.05 | -50 | [M-H]^-^ |
| PI 18:1_18:1 | 861.55 | 281.249 | 5.22 | -50 | [M-H]^-^ |
| PI 18:1_18:2 | 859.534 | 279.233 | 5.05 | -50 | [M-H]^-^ |
| PI 18:1_20:4 | 883.534 | 303.233 | 4.91 | -50 | [M-H]^-^ |
| SM 18:1;O2/14:0 | 675.5 | 184.1 | 5.28 | 43 | [M+H]^+^ |
| SM 18:1;O2/16:0 | 703.6 | 184.1 | 5.57 | 43 | [M+H]^+^ |
| SM 18:1;O2/18:0 | 731.6 | 184.1 | 5.9 | 43 | [M+H]^+^ |
| SM 18:1;O2/20:0 | 759.6 | 184.1 | 6.25 | 43 | [M+H]^+^ |
| SM 18:1;O2/20:1 | 757.6 | 184.1 | 6.02 | 43 | [M+H]^+^ |
| SM 18:1;O2/22:0 | 787.7 | 184.1 | 6.61 | 43 | [M+H]^+^ |
| SM 18:1;O2/22:1 | 785.7 | 184.1 | 5.79 | 43 | [M+H]^+^ |
| SM 18:1;O2/24:0 | 815.7 | 184.1 | 6.98 | 43 | [M+H]^+^ |
| SM 18:1;O2/24:1 | 813.7 | 184.1 | 6.63 | 43 | [M+H]^+^ |
| SM 18:1;O2/26:0 | 843.7 | 184.1 | 7.13 | 43 | [M+H]^+^ |
| SM 18:1;O2/26:1 | 841.7 | 184.1 | 6.97 | 25 | [M+H]^+^ |
| SPBP 18:1;O2 | 380.3 | 264.2 | 3.31 | 10 | [M+H]^+^ |
| TG 46:0_FA14:0 | 796.7 | 551.503 | 8.7 | 38 | [M+NH_4_]^+^ |
| TG 46:0_FA16:0 | 796.7 | 523.472 | 8.7 | 38 | [M+NH_4_]^+^ |
| TG 46:1_FA14:0 | 794.7 | 549.5 | 8.49 | 38 | [M+NH_4_]^+^ |
| TG 46:1_FA16:0 | 794.7 | 521.4 | 8.49 | 38 | [M+NH_4_]^+^ |
| TG 46:1_FA16:1 | 794.7 | 523.472 | 8.49 | 38 | [M+NH_4_]^+^ |
| TG 46:1_FA18:1 | 794.7 | 495.441 | 8.49 | 38 | [M+NH_4_]^+^ |
| TG 46:2_FA14:0 | 792.7 | 547.5 | 8.3 | 38 | [M+NH_4_]^+^ |
| TG 46:2_FA16:0 | 792.7 | 519.441 | 8.3 | 38 | [M+NH_4_]^+^ |
| TG 46:2_FA16:1 | 792.7 | 521.4 | 8.3 | 38 | [M+NH_4_]^+^ |
| TG 46:2_FA18:1 | 792.7 | 493.425 | 8.3 | 38 | [M+NH_4_]^+^ |
| TG 46:2_FA18:2 | 792.7 | 495.441 | 8.3 | 38 | [M+NH_4_]^+^ |
| TG 46:3_FA16:0 | 790.7 | 517.4 | 8.06 | 38 | [M+NH_4_]^+^ |
| TG 46:3_FA18:1 | 790.7 | 491.409 | 8.06 | 38 | [M+NH_4_]^+^ |
| TG 46:3_FA18:2 | 790.7 | 493.425 | 8.06 | 38 | [M+NH_4_]^+^ |
| TG 46:4_FA18:2 | 788.7 | 491.409 | 8.06 | 38 | [M+NH_4_]^+^ |
| TG 48:0_FA14:0 | 824.7 | 579.5 | 8.96 | 38 | [M+NH_4_]^+^ |
| TG 48:0_FA16:0 | 824.7 | 551.4 | 8.96 | 38 | [M+NH_4_]^+^ |
| TG 48:0_FA18:0 | 824.7 | 523.472 | 8.96 | 38 | [M+NH_4_]^+^ |
| TG 48:1_FA14:0 | 822.7 | 577.5 | 8.77 | 38 | [M+NH_4_]^+^ |
| TG 48:1_FA16:0 | 822.7 | 549.4 | 8.77 | 38 | [M+NH_4_]^+^ |
| TG 48:1_FA16:1 | 822.7 | 551.4 | 8.77 | 38 | [M+NH_4_]^+^ |
| TG 48:1_FA18:1 | 822.7 | 523.472 | 8.77 | 38 | [M+NH_4_]^+^ |
| TG 48:2_FA14:0 | 820.7 | 575.5 | 8.55 | 38 | [M+NH_4_]^+^ |
| TG 48:2_FA16:0 | 820.7 | 547.4 | 8.55 | 38 | [M+NH_4_]^+^ |
| TG 48:2_FA16:1 | 820.7 | 549.4 | 8.55 | 38 | [M+NH_4_]^+^ |
| TG 48:2_FA18:1 | 820.7 | 521.4 | 8.55 | 38 | [M+NH_4_]^+^ |
| TG 48:2_FA18:2 | 820.7 | 523.472 | 8.55 | 38 | [M+NH_4_]^+^ |
| TG 48:3_FA14:0 | 818.7 | 573.5 | 8.34 | 38 | [M+NH_4_]^+^ |
| TG 48:3_FA16:0 | 818.7 | 545.4 | 8.34 | 38 | [M+NH_4_]^+^ |
| TG 48:3_FA16:1 | 818.7 | 547.4 | 8.34 | 38 | [M+NH_4_]^+^ |
| TG 48:3_FA18:1 | 818.7 | 519.441 | 8.34 | 38 | [M+NH_4_]^+^ |
| TG 48:3_FA18:2 | 818.7 | 521.4 | 8.34 | 38 | [M+NH_4_]^+^ |
| TG 48:3_FA18:3 | 818.7 | 523.472 | 8.34 | 38 | [M+NH_4_]^+^ |
| TG 48:4_FA14:0 | 816.7 | 571.5 | 8.13 | 38 | [M+NH_4_]^+^ |
| TG 48:4_FA16:0 | 816.7 | 543.4 | 8.13 | 38 | [M+NH_4_]^+^ |
| TG 48:4_FA16:1 | 816.7 | 545.4 | 8.13 | 38 | [M+NH_4_]^+^ |
| TG 48:4_FA18:1 | 816.7 | 517.4 | 8.13 | 38 | [M+NH_4_]^+^ |
| TG 48:4_FA18:2 | 816.7 | 519.441 | 8.13 | 38 | [M+NH_4_]^+^ |
| TG 48:4_FA18:3 | 816.7 | 521.4 | 8.13 | 38 | [M+NH_4_]^+^ |
| TG 48:5_FA18:2 | 814.7 | 517.4 | 7.88 | 38 | [M+NH_4_]^+^ |
| TG 49:0_FA16:0 | 838.8 | 565.5 | 9.07 | 38 | [M+NH_4_]^+^ |
| TG 49:1_FA16:0 | 836.8 | 563.5 | 8.89 | 38 | [M+NH_4_]^+^ |
| TG 49:1_FA18:1 | 836.8 | 537.5 | 8.89 | 38 | [M+NH_4_]^+^ |
| TG 49:2_FA16:0 | 834.8 | 561.5 | 8.67 | 38 | [M+NH_4_]^+^ |
| TG 49:2_FA16:1 | 834.8 | 563.5 | 8.67 | 38 | [M+NH_4_]^+^ |
| TG 49:2_FA18:1 | 834.8 | 535.5 | 8.67 | 38 | [M+NH_4_]^+^ |
| TG 49:2_FA18:2 | 834.8 | 537.5 | 8.67 | 38 | [M+NH_4_]^+^ |
| TG 49:3_FA16:0 | 832.8 | 559.5 | 8.47 | 38 | [M+NH_4_]^+^ |
| TG 49:3_FA16:1 | 832.8 | 561.5 | 8.47 | 38 | [M+NH_4_]^+^ |
| TG 49:3_FA18:2 | 832.8 | 535.5 | 8.47 | 38 | [M+NH_4_]^+^ |
| TG 50:0_FA16:0 | 852.8 | 579.5 | 9.03 | 38 | [M+NH_4_]^+^ |
| TG 50:0_FA18:0 | 852.8 | 551.503 | 9.23 | 38 | [M+NH_4_]^+^ |
| TG 50:1_FA14:0 | 850.8 | 605.6 | 9.04 | 38 | [M+NH_4_]^+^ |
| TG 50:1_FA16:0 | 850.8 | 577.5 | 9.04 | 38 | [M+NH_4_]^+^ |
| TG 50:1_FA16:1 | 850.8 | 579.5 | 9.04 | 38 | [M+NH_4_]^+^ |
| TG 50:1_FA18:0 | 850.8 | 549.5 | 9.04 | 38 | [M+NH_4_]^+^ |
| TG 50:1_FA18:1 | 850.8 | 551.503 | 9.04 | 38 | [M+NH_4_]^+^ |
| TG 50:2_FA14:0 | 848.8 | 603.6 | 8.83 | 38 | [M+NH_4_]^+^ |
| TG 50:2_FA16:0 | 848.8 | 575.5 | 8.83 | 38 | [M+NH_4_]^+^ |
| TG 50:2_FA16:1 | 848.8 | 577.5 | 8.83 | 38 | [M+NH_4_]^+^ |
| TG 50:2_FA18:0 | 848.8 | 547.5 | 8.83 | 38 | [M+NH_4_]^+^ |
| TG 50:2_FA18:1 | 848.8 | 549.5 | 8.83 | 38 | [M+NH_4_]^+^ |
| TG 50:2_FA18:2 | 848.8 | 551.503 | 8.83 | 38 | [M+NH_4_]^+^ |
| TG 50:3_FA14:0 | 846.8 | 601.6 | 8.62 | 38 | [M+NH_4_]^+^ |
| TG 50:3_FA16:0 | 846.8 | 573.5 | 8.62 | 38 | [M+NH_4_]^+^ |
| TG 50:3_FA16:1 | 846.8 | 575.5 | 8.62 | 38 | [M+NH_4_]^+^ |
| TG 50:3_FA18:0 | 846.8 | 545.5 | 8.62 | 38 | [M+NH_4_]^+^ |
| TG 50:3_FA18:1 | 846.8 | 547.5 | 8.62 | 38 | [M+NH_4_]^+^ |
| TG 50:3_FA18:2 | 846.8 | 549.5 | 8.62 | 38 | [M+NH_4_]^+^ |
| TG 50:3_FA18:3 | 846.8 | 551.503 | 8.62 | 38 | [M+NH_4_]^+^ |
| TG 50:4_FA14:0 | 844.6 | 599.4 | 8.4 | 38 | [M+NH_4_]^+^ |
| TG 50:4_FA16:0 | 844.6 | 571.3 | 8.4 | 38 | [M+NH_4_]^+^ |
| TG 50:4_FA16:1 | 844.6 | 573.3 | 8.4 | 38 | [M+NH_4_]^+^ |
| TG 50:4_FA18:1 | 844.6 | 545.3 | 8.4 | 38 | [M+NH_4_]^+^ |
| TG 50:4_FA18:2 | 844.6 | 547.3 | 8.4 | 38 | [M+NH_4_]^+^ |
| TG 50:4_FA18:3 | 844.6 | 549.3 | 8.4 | 38 | [M+NH_4_]^+^ |
| TG 50:4_FA20:4 | 844.6 | 523.3 | 8.4 | 38 | [M+NH_4_]^+^ |
| TG 50:5_FA14:0 | 842.6 | 597.4 | 8.22 | 38 | [M+NH_4_]^+^ |
| TG 50:5_FA16:0 | 842.6 | 569.3 | 8.22 | 38 | [M+NH_4_]^+^ |
| TG 50:5_FA16:1 | 842.6 | 571.3 | 8.22 | 38 | [M+NH_4_]^+^ |
| TG 50:5_FA18:1 | 842.6 | 543.3 | 8.22 | 38 | [M+NH_4_]^+^ |
| TG 50:5_FA18:2 | 842.6 | 545.3 | 8.22 | 38 | [M+NH_4_]^+^ |
| TG 50:5_FA18:3 | 842.6 | 547.3 | 8.22 | 38 | [M+NH_4_]^+^ |
| TG 51:0_FA16:0 | 866.8 | 593.5 | 9.16 | 38 | [M+NH_4_]^+^ |
| TG 51:0_FA17:0 | 866.8 | 579.5 | 9.16 | 38 | [M+NH_4_]^+^ |
| TG 51:1_FA16:0 | 864.8 | 591.5 | 9.15 | 38 | [M+NH_4_]^+^ |
| TG 51:1_FA17:0 | 864.8 | 577.5 | 9.15 | 38 | [M+NH_4_]^+^ |
| TG 51:1_FA18:0 | 864.8 | 563.5 | 9.15 | 38 | [M+NH_4_]^+^ |
| TG 51:1_FA18:1 | 864.8 | 565.5 | 9.15 | 38 | [M+NH_4_]^+^ |
| TG 51:2_FA16:0 | 862.8 | 589.5 | 8.96 | 38 | [M+NH_4_]^+^ |
| TG 51:2_FA16:1 | 862.8 | 591.5 | 8.96 | 38 | [M+NH_4_]^+^ |
| TG 51:2_FA17:0 | 862.8 | 575.5 | 8.96 | 38 | [M+NH_4_]^+^ |
| TG 51:2_FA18:1 | 862.8 | 563.5 | 8.96 | 38 | [M+NH_4_]^+^ |
| TG 51:2_FA18:2 | 862.8 | 565.5 | 8.96 | 38 | [M+NH_4_]^+^ |
| TG 51:3_FA16:1 | 860.8 | 589.5 | 8.78 | 38 | [M+NH_4_]^+^ |
| TG 51:3_FA17:0 | 860.8 | 573.5 | 8.78 | 38 | [M+NH_4_]^+^ |
| TG 51:3_FA18:2 | 860.8 | 563.5 | 8.78 | 38 | [M+NH_4_]^+^ |
| TG 51:4_FA16:1 | 858.8 | 587.5 | 8.56 | 38 | [M+NH_4_]^+^ |
| TG 51:4_FA18:2 | 858.8 | 561.5 | 8.56 | 38 | [M+NH_4_]^+^ |
| TG 51:4_FA18:3 | 858.8 | 563.5 | 8.56 | 38 | [M+NH_4_]^+^ |
| TG 51:5_FA18:2 | 856.8 | 559.5 | 8.56 | 38 | [M+NH_4_]^+^ |
| TG 51:5_FA18:3 | 856.8 | 561.5 | 8.56 | 38 | [M+NH_4_]^+^ |
| TG 52:0_FA16:0 | 880.8 | 607.5 | 9.5 | 38 | [M+NH_4_]^+^ |
| TG 52:0_FA18:0 | 880.8 | 579.5 | 9.5 | 38 | [M+NH_4_]^+^ |
| TG 52:1_FA16:0 | 878.8 | 605.5 | 9.31 | 38 | [M+NH_4_]^+^ |
| TG 52:1_FA16:1 | 878.8 | 607.5 | 9.31 | 38 | [M+NH_4_]^+^ |
| TG 52:1_FA18:0 | 878.8 | 577.5 | 9.31 | 38 | [M+NH_4_]^+^ |
| TG 52:1_FA18:1 | 878.8 | 579.5 | 9.31 | 38 | [M+NH_4_]^+^ |
| TG 52:1_FA20:1 | 878.8 | 551.503 | 9.31 | 38 | [M+NH_4_]^+^ |
| TG 52:2_FA16:0 | 876.8 | 603.5 | 9.1 | 38 | [M+NH_4_]^+^ |
| TG 52:2_FA16:1 | 876.8 | 605.5 | 9.1 | 38 | [M+NH_4_]^+^ |
| TG 52:2_FA18:0 | 876.8 | 575.5 | 9.1 | 38 | [M+NH_4_]^+^ |
| TG 52:2_FA18:1 | 876.8 | 577.5 | 9.1 | 38 | [M+NH_4_]^+^ |
| TG 52:2_FA18:2 | 876.8 | 579.5 | 9.1 | 38 | [M+NH_4_]^+^ |
| TG 52:2_FA20:0 | 876.8 | 547.5 | 9.1 | 38 | [M+NH_4_]^+^ |
| TG 52:2_FA20:1 | 876.8 | 549.5 | 9.1 | 38 | [M+NH_4_]^+^ |
| TG 52:2_FA20:2 | 876.8 | 551.503 | 9.1 | 38 | [M+NH_4_]^+^ |
| TG 52:3_FA14:0 | 874.8 | 629.6 | 8.9 | 38 | [M+NH_4_]^+^ |
| TG 52:3_FA16:0 | 874.8 | 601.5 | 8.9 | 38 | [M+NH_4_]^+^ |
| TG 52:3_FA16:1 | 874.8 | 603.5 | 8.9 | 38 | [M+NH_4_]^+^ |
| TG 52:3_FA18:0 | 874.8 | 573.5 | 8.9 | 38 | [M+NH_4_]^+^ |
| TG 52:3_FA18:1 | 874.8 | 575.5 | 8.9 | 38 | [M+NH_4_]^+^ |
| TG 52:3_FA18:2 | 874.8 | 577.5 | 8.9 | 38 | [M+NH_4_]^+^ |
| TG 52:3_FA18:3 | 874.8 | 579.5 | 8.9 | 38 | [M+NH_4_]^+^ |
| TG 52:3_FA20:0 | 874.8 | 545.5 | 8.9 | 38 | [M+NH_4_]^+^ |
| TG 52:3_FA20:1 | 874.8 | 547.5 | 8.9 | 38 | [M+NH_4_]^+^ |
| TG 52:3_FA20:2 | 874.8 | 549.5 | 8.9 | 38 | [M+NH_4_]^+^ |
| TG 52:3_FA20:3 | 874.8 | 551.503 | 8.9 | 38 | [M+NH_4_]^+^ |
| TG 52:4_FA14:0 | 872.8 | 627.6 | 8.7 | 38 | [M+NH_4_]^+^ |
| TG 52:4_FA16:0 | 872.8 | 599.5 | 8.7 | 38 | [M+NH_4_]^+^ |
| TG 52:4_FA16:1 | 872.8 | 601.5 | 8.7 | 38 | [M+NH_4_]^+^ |
| TG 52:4_FA18:0 | 872.8 | 571.5 | 8.7 | 38 | [M+NH_4_]^+^ |
| TG 52:4_FA18:1 | 872.8 | 573.5 | 8.7 | 38 | [M+NH_4_]^+^ |
| TG 52:4_FA18:2 | 872.8 | 575.5 | 8.7 | 38 | [M+NH_4_]^+^ |
| TG 52:4_FA18:3 | 872.8 | 577.5 | 8.7 | 38 | [M+NH_4_]^+^ |
| TG 52:4_FA20:0 | 872.8 | 543.5 | 8.7 | 38 | [M+NH_4_]^+^ |
| TG 52:4_FA20:2 | 872.8 | 547.5 | 8.7 | 38 | [M+NH_4_]^+^ |
| TG 52:4_FA20:3 | 872.8 | 549.5 | 8.7 | 38 | [M+NH_4_]^+^ |
| TG 52:4_FA20:4 | 872.8 | 551.503 | 8.7 | 38 | [M+NH_4_]^+^ |
| TG 52:4_FA22:4 | 872.8 | 523.472 | 8.7 | 38 | [M+NH_4_]^+^ |
| TG 52:5_FA14:0 | 870.8 | 625.6 | 8.5 | 38 | [M+NH_4_]^+^ |
| TG 52:5_FA16:0 | 870.8 | 597.5 | 8.5 | 38 | [M+NH_4_]^+^ |
| TG 52:5_FA16:1 | 870.8 | 599.5 | 8.5 | 38 | [M+NH_4_]^+^ |
| TG 52:5_FA18:1 | 870.8 | 571.5 | 8.5 | 38 | [M+NH_4_]^+^ |
| TG 52:5_FA18:2 | 870.8 | 573.5 | 8.5 | 38 | [M+NH_4_]^+^ |
| TG 52:5_FA18:3 | 870.8 | 575.5 | 8.5 | 38 | [M+NH_4_]^+^ |
| TG 52:5_FA20:3 | 870.8 | 547.5 | 8.5 | 38 | [M+NH_4_]^+^ |
| TG 52:5_FA20:4 | 870.8 | 549.5 | 8.5 | 38 | [M+NH_4_]^+^ |
| TG 52:5_FA20:5 | 870.8 | 551.503 | 8.5 | 38 | [M+NH_4_]^+^ |
| TG 52:5_FA22:5 | 870.8 | 523.472 | 8.5 | 38 | [M+NH_4_]^+^ |
| TG 52:6_FA14:0 | 868.8 | 623.6 | 8.3 | 38 | [M+NH_4_]^+^ |
| TG 52:6_FA16:0 | 868.8 | 595.5 | 8.3 | 38 | [M+NH_4_]^+^ |
| TG 52:6_FA16:1 | 868.8 | 597.5 | 8.3 | 38 | [M+NH_4_]^+^ |
| TG 52:6_FA18:1 | 868.8 | 569.5 | 8.3 | 38 | [M+NH_4_]^+^ |
| TG 52:6_FA18:2 | 868.8 | 571.5 | 8.3 | 38 | [M+NH_4_]^+^ |
| TG 52:6_FA18:3 | 868.8 | 573.5 | 8.3 | 38 | [M+NH_4_]^+^ |
| TG 52:6_FA20:4 | 868.8 | 547.5 | 8.3 | 38 | [M+NH_4_]^+^ |
| TG 52:6_FA20:5 | 868.8 | 549.5 | 8.3 | 38 | [M+NH_4_]^+^ |
| TG 52:6_FA22:6 | 868.8 | 523.472 | 8.3 | 38 | [M+NH_4_]^+^ |
| TG 52:7_FA20:5 | 866.7 | 547.4 | 8.1 | 38 | [M+NH_4_]^+^ |
| TG 53:1_FA17:0 | 892.8 | 605.5 | 9.4 | 38 | [M+NH_4_]^+^ |
| TG 53:1_FA18:0 | 892.8 | 591.5 | 9.4 | 38 | [M+NH_4_]^+^ |
| TG 53:1_FA18:1 | 892.8 | 593.5 | 9.4 | 38 | [M+NH_4_]^+^ |
| TG 53:2_FA16:0 | 890.8 | 617.5 | 9.2 | 38 | [M+NH_4_]^+^ |
| TG 53:2_FA17:0 | 890.8 | 603.5 | 9.2 | 38 | [M+NH_4_]^+^ |
| TG 53:2_FA18:1 | 890.8 | 591.5 | 9.2 | 38 | [M+NH_4_]^+^ |
| TG 53:2_FA18:2 | 890.8 | 593.5 | 9.2 | 38 | [M+NH_4_]^+^ |
| TG 53:3_FA16:0 | 888.8 | 615.5 | 9 | 38 | [M+NH_4_]^+^ |
| TG 53:3_FA17:0 | 888.8 | 601.5 | 9 | 38 | [M+NH_4_]^+^ |
| TG 53:3_FA18:2 | 888.8 | 591.5 | 9 | 38 | [M+NH_4_]^+^ |
| TG 53:4_FA16:0 | 886.8 | 613.5 | 8.8 | 38 | [M+NH_4_]^+^ |
| TG 53:4_FA17:0 | 886.8 | 599.5 | 8.8 | 38 | [M+NH_4_]^+^ |
| TG 53:4_FA18:2 | 886.8 | 589.5 | 8.8 | 38 | [M+NH_4_]^+^ |
| TG 53:4_FA18:3 | 886.8 | 591.5 | 8.8 | 38 | [M+NH_4_]^+^ |
| TG 53:5_FA20:4 | 884.8 | 563.5 | 8.8 | 38 | [M+NH_4_]^+^ |
| TG 53:6_FA20:4 | 882.8 | 561.5 | 8.8 | 38 | [M+NH_4_]^+^ |
| TG 54:0_FA18:0 | 908.8 | 607.5 | 9.75 | 38 | [M+NH_4_]^+^ |
| TG 54:1_FA16:0 | 906.8 | 633.5 | 9.55 | 38 | [M+NH_4_]^+^ |
| TG 54:1_FA18:0 | 906.8 | 605.5 | 9.55 | 38 | [M+NH_4_]^+^ |
| TG 54:1_FA18:1 | 906.8 | 607.5 | 9.55 | 38 | [M+NH_4_]^+^ |
| TG 54:1_FA20:0 | 906.8 | 577.5 | 9.55 | 38 | [M+NH_4_]^+^ |
| TG 54:1_FA20:1 | 906.8 | 579.5 | 9.55 | 38 | [M+NH_4_]^+^ |
| TG 54:2_FA16:0 | 904.8 | 631.5 | 9.3 | 38 | [M+NH_4_]^+^ |
| TG 54:2_FA18:0 | 904.8 | 603.5 | 9.3 | 38 | [M+NH_4_]^+^ |
| TG 54:2_FA18:1 | 904.8 | 605.5 | 9.3 | 38 | [M+NH_4_]^+^ |
| TG 54:2_FA18:2 | 904.8 | 607.5 | 9.3 | 38 | [M+NH_4_]^+^ |
| TG 54:2_FA20:0 | 904.8 | 575.5 | 9.3 | 38 | [M+NH_4_]^+^ |
| TG 54:2_FA20:1 | 904.8 | 577.5 | 9.3 | 38 | [M+NH_4_]^+^ |
| TG 54:2_FA20:2 | 904.8 | 579.5 | 9.3 | 38 | [M+NH_4_]^+^ |
| TG 54:3_FA16:0 | 902.8 | 629.5 | 9.1 | 38 | [M+NH_4_]^+^ |
| TG 54:3_FA16:1 | 902.8 | 631.5 | 9.1 | 38 | [M+NH_4_]^+^ |
| TG 54:3_FA18:0 | 902.8 | 601.5 | 9.1 | 38 | [M+NH_4_]^+^ |
| TG 54:3_FA18:1 | 902.8 | 603.5 | 9.1 | 38 | [M+NH_4_]^+^ |
| TG 54:3_FA18:2 | 902.8 | 605.5 | 9.1 | 38 | [M+NH_4_]^+^ |
| TG 54:3_FA18:3 | 902.8 | 607.5 | 9.1 | 38 | [M+NH_4_]^+^ |
| TG 54:3_FA20:1 | 902.8 | 575.5 | 9.1 | 38 | [M+NH_4_]^+^ |
| TG 54:3_FA20:2 | 902.8 | 577.5 | 9.1 | 38 | [M+NH_4_]^+^ |
| TG 54:3_FA20:3 | 902.8 | 579.5 | 9.1 | 38 | [M+NH_4_]^+^ |
| TG 54:4_FA16:0 | 900.8 | 627.5 | 8.9 | 38 | [M+NH_4_]^+^ |
| TG 54:4_FA16:1 | 900.8 | 629.5 | 8.9 | 38 | [M+NH_4_]^+^ |
| TG 54:4_FA18:0 | 900.8 | 599.5 | 8.9 | 38 | [M+NH_4_]^+^ |
| TG 54:4_FA18:1 | 900.8 | 601.5 | 8.9 | 38 | [M+NH_4_]^+^ |
| TG 54:4_FA18:2 | 900.8 | 603.5 | 8.9 | 38 | [M+NH_4_]^+^ |
| TG 54:4_FA18:3 | 900.8 | 605.5 | 8.9 | 38 | [M+NH_4_]^+^ |
| TG 54:4_FA20:1 | 900.8 | 573.5 | 8.9 | 38 | [M+NH_4_]^+^ |
| TG 54:4_FA20:2 | 900.8 | 575.5 | 8.9 | 38 | [M+NH_4_]^+^ |
| TG 54:4_FA20:3 | 900.8 | 577.5 | 8.9 | 38 | [M+NH_4_]^+^ |
| TG 54:4_FA20:4 | 900.8 | 579.5 | 8.9 | 38 | [M+NH_4_]^+^ |
| TG 54:4_FA22:4 | 900.8 | 551.503 | 8.9 | 38 | [M+NH_4_]^+^ |
| TG 54:5_FA16:0 | 898.8 | 625.5 | 8.75 | 38 | [M+NH_4_]^+^ |
| TG 54:5_FA16:1 | 898.8 | 627.5 | 8.75 | 38 | [M+NH_4_]^+^ |
| TG 54:5_FA18:0 | 898.8 | 597.5 | 8.75 | 38 | [M+NH_4_]^+^ |
| TG 54:5_FA18:1 | 898.8 | 599.5 | 8.75 | 38 | [M+NH_4_]^+^ |
| TG 54:5_FA18:2 | 898.8 | 601.5 | 8.75 | 38 | [M+NH_4_]^+^ |
| TG 54:5_FA18:3 | 898.8 | 603.5 | 8.75 | 38 | [M+NH_4_]^+^ |
| TG 54:5_FA20:2 | 898.8 | 573.5 | 8.75 | 38 | [M+NH_4_]^+^ |
| TG 54:5_FA20:3 | 898.8 | 575.5 | 8.75 | 38 | [M+NH_4_]^+^ |
| TG 54:5_FA20:4 | 898.8 | 577.5 | 8.75 | 38 | [M+NH_4_]^+^ |
| TG 54:5_FA20:5 | 898.8 | 579.5 | 8.75 | 38 | [M+NH_4_]^+^ |
| TG 54:5_FA22:4 | 898.8 | 549.5 | 8.75 | 38 | [M+NH_4_]^+^ |
| TG 54:5_FA22:5 | 898.8 | 551.503 | 8.75 | 38 | [M+NH_4_]^+^ |
| TG 54:6_FA16:0 | 896.8 | 623.5 | 8.55 | 38 | [M+NH_4_]^+^ |
| TG 54:6_FA16:1 | 896.8 | 625.5 | 8.55 | 38 | [M+NH_4_]^+^ |
| TG 54:6_FA18:1 | 896.8 | 597.5 | 8.55 | 38 | [M+NH_4_]^+^ |
| TG 54:6_FA18:2 | 896.8 | 599.5 | 8.55 | 38 | [M+NH_4_]^+^ |
| TG 54:6_FA18:3 | 896.8 | 601.5 | 8.55 | 38 | [M+NH_4_]^+^ |
| TG 54:6_FA20:3 | 896.8 | 573.5 | 8.55 | 38 | [M+NH_4_]^+^ |
| TG 54:6_FA20:4 | 896.8 | 575.5 | 8.55 | 38 | [M+NH_4_]^+^ |
| TG 54:6_FA20:5 | 896.8 | 577.5 | 8.55 | 38 | [M+NH_4_]^+^ |
| TG 54:6_FA22:5 | 896.8 | 549.5 | 8.55 | 38 | [M+NH_4_]^+^ |
| TG 54:6_FA22:6 | 896.8 | 551.503 | 8.55 | 38 | [M+NH_4_]^+^ |
| TG 54:7_FA16:1 | 894.8 | 623.5 | 8.35 | 38 | [M+NH_4_]^+^ |
| TG 54:7_FA18:1 | 894.8 | 595.5 | 8.35 | 38 | [M+NH_4_]^+^ |
| TG 54:7_FA18:2 | 894.8 | 597.5 | 8.35 | 38 | [M+NH_4_]^+^ |
| TG 54:7_FA18:3 | 894.8 | 599.5 | 8.35 | 38 | [M+NH_4_]^+^ |
| TG 54:7_FA20:4 | 894.8 | 573.5 | 8.35 | 38 | [M+NH_4_]^+^ |
| TG 54:7_FA20:5 | 894.8 | 575.5 | 8.35 | 38 | [M+NH_4_]^+^ |
| TG 54:7_FA22:5 | 894.8 | 547.5 | 8.35 | 38 | [M+NH_4_]^+^ |
| TG 54:7_FA22:6 | 894.8 | 549.5 | 8.35 | 38 | [M+NH_4_]^+^ |
| TG 54:8_FA18:2 | 892.8 | 595.5 | 8.1 | 38 | [M+NH_4_]^+^ |
| TG 54:8_FA18:3 | 892.8 | 597.5 | 8.1 | 38 | [M+NH_4_]^+^ |
| TG 54:8_FA20:4 | 892.8 | 571.5 | 8.1 | 38 | [M+NH_4_]^+^ |
| TG 54:8_FA20:5 | 892.8 | 573.5 | 8.1 | 38 | [M+NH_4_]^+^ |
| TG 54:8_FA22:6 | 892.8 | 547.5 | 8.1 | 38 | [M+NH_4_]^+^ |
| TG 55:2_FA18:1 | 918.8 | 619.5 | 9.5 | 38 | [M+NH_4_]^+^ |
| TG 55:3_FA18:1 | 916.8 | 617.5 | 9.3 | 38 | [M+NH_4_]^+^ |
| TG 55:3_FA18:2 | 916.8 | 619.5 | 9.3 | 38 | [M+NH_4_]^+^ |
| TG 55:4_FA18:1 | 914.8 | 615.5 | 9.1 | 38 | [M+NH_4_]^+^ |
| TG 55:4_FA18:2 | 914.8 | 617.5 | 9.1 | 38 | [M+NH_4_]^+^ |
| TG 55:5_FA18:1 | 912.8 | 613.5 | 8.9 | 38 | [M+NH_4_]^+^ |
| TG 55:5_FA18:2 | 912.8 | 615.5 | 8.9 | 38 | [M+NH_4_]^+^ |
| TG 55:5_FA20:4 | 912.8 | 591.5 | 8.9 | 38 | [M+NH_4_]^+^ |
| TG 55:7_FA22:6 | 908.8 | 563.5 | 8.6 | 38 | [M+NH_4_]^+^ |
| TG 56:1_FA16:0 | 934.9 | 661.6 | 9.8 | 38 | [M+NH_4_]^+^ |
| TG 56:1_FA18:1 | 934.9 | 635.6 | 9.8 | 38 | [M+NH_4_]^+^ |
| TG 56:2_FA16:0 | 932.9 | 659.6 | 9.6 | 38 | [M+NH_4_]^+^ |
| TG 56:2_FA18:0 | 932.9 | 631.6 | 9.6 | 38 | [M+NH_4_]^+^ |
| TG 56:2_FA20:0 | 932.9 | 603.6 | 9.6 | 38 | [M+NH_4_]^+^ |
| TG 56:2_FA20:1 | 932.9 | 605.6 | 9.6 | 38 | [M+NH_4_]^+^ |
| TG 56:3_FA16:0 | 930.8 | 657.5 | 9.42 | 38 | [M+NH_4_]^+^ |
| TG 56:3_FA18:0 | 930.8 | 629.5 | 9.42 | 38 | [M+NH_4_]^+^ |
| TG 56:3_FA18:1 | 930.8 | 631.5 | 9.42 | 38 | [M+NH_4_]^+^ |
| TG 56:3_FA18:2 | 930.8 | 633.5 | 9.42 | 38 | [M+NH_4_]^+^ |
| TG 56:3_FA20:0 | 930.8 | 601.5 | 9.42 | 38 | [M+NH_4_]^+^ |
| TG 56:3_FA20:1 | 930.8 | 603.5 | 9.42 | 38 | [M+NH_4_]^+^ |
| TG 56:3_FA20:2 | 930.8 | 605.5 | 9.42 | 38 | [M+NH_4_]^+^ |
| TG 56:4_FA16:0 | 928.8 | 655.5 | 9.19 | 38 | [M+NH_4_]^+^ |
| TG 56:4_FA18:0 | 928.8 | 627.5 | 9.19 | 38 | [M+NH_4_]^+^ |
| TG 56:4_FA18:1 | 928.8 | 629.5 | 9.19 | 38 | [M+NH_4_]^+^ |
| TG 56:4_FA18:2 | 928.8 | 631.5 | 9.19 | 38 | [M+NH_4_]^+^ |
| TG 56:4_FA20:1 | 928.8 | 601.5 | 9.19 | 38 | [M+NH_4_]^+^ |
| TG 56:4_FA20:2 | 928.8 | 603.5 | 9.19 | 38 | [M+NH_4_]^+^ |
| TG 56:4_FA20:3 | 928.8 | 605.5 | 9.19 | 38 | [M+NH_4_]^+^ |
| TG 56:4_FA20:4 | 928.8 | 607.5 | 9.19 | 38 | [M+NH_4_]^+^ |
| TG 56:4_FA22:4 | 928.8 | 579.5 | 9.19 | 38 | [M+NH_4_]^+^ |
| TG 56:5_FA16:0 | 926.8 | 653.5 | 9 | 38 | [M+NH_4_]^+^ |
| TG 56:5_FA18:0 | 926.8 | 625.5 | 9 | 38 | [M+NH_4_]^+^ |
| TG 56:5_FA18:1 | 926.8 | 627.5 | 9 | 38 | [M+NH_4_]^+^ |
| TG 56:5_FA18:2 | 926.8 | 629.5 | 9 | 38 | [M+NH_4_]^+^ |
| TG 56:5_FA20:1 | 926.8 | 599.5 | 9 | 38 | [M+NH_4_]^+^ |
| TG 56:5_FA20:2 | 926.8 | 601.5 | 9 | 38 | [M+NH_4_]^+^ |
| TG 56:5_FA20:3 | 926.8 | 603.5 | 9 | 38 | [M+NH_4_]^+^ |
| TG 56:5_FA20:4 | 926.8 | 605.5 | 9 | 38 | [M+NH_4_]^+^ |
| TG 56:5_FA22:4 | 926.8 | 577.5 | 9 | 38 | [M+NH_4_]^+^ |
| TG 56:5_FA22:5 | 926.8 | 579.5 | 9 | 38 | [M+NH_4_]^+^ |
| TG 56:6_FA16:0 | 924.8 | 651.5 | 8.82 | 38 | [M+NH_4_]^+^ |
| TG 56:6_FA18:0 | 924.8 | 623.5 | 8.82 | 38 | [M+NH_4_]^+^ |
| TG 56:6_FA18:1 | 924.8 | 625.5 | 8.82 | 38 | [M+NH_4_]^+^ |
| TG 56:6_FA18:2 | 924.8 | 627.5 | 8.82 | 38 | [M+NH_4_]^+^ |
| TG 56:6_FA18:3 | 924.8 | 629.5 | 8.82 | 38 | [M+NH_4_]^+^ |
| TG 56:6_FA20:2 | 924.8 | 599.5 | 8.82 | 38 | [M+NH_4_]^+^ |
| TG 56:6_FA20:3 | 924.8 | 601.5 | 8.82 | 38 | [M+NH_4_]^+^ |
| TG 56:6_FA20:4 | 924.8 | 603.5 | 8.82 | 38 | [M+NH_4_]^+^ |
| TG 56:6_FA20:5 | 924.8 | 605.5 | 8.82 | 38 | [M+NH_4_]^+^ |
| TG 56:6_FA22:4 | 924.8 | 575.5 | 8.82 | 38 | [M+NH_4_]^+^ |
| TG 56:6_FA22:5 | 924.8 | 577.5 | 8.82 | 38 | [M+NH_4_]^+^ |
| TG 56:6_FA22:6 | 924.8 | 579.5 | 8.82 | 38 | [M+NH_4_]^+^ |
| TG 56:7_FA16:0 | 922.8 | 649.5 | 8.63 | 38 | [M+NH_4_]^+^ |
| TG 56:7_FA16:1 | 922.8 | 651.5 | 8.63 | 38 | [M+NH_4_]^+^ |
| TG 56:7_FA18:0 | 922.8 | 621.5 | 8.63 | 38 | [M+NH_4_]^+^ |
| TG 56:7_FA18:1 | 922.8 | 623.5 | 8.63 | 38 | [M+NH_4_]^+^ |
| TG 56:7_FA18:2 | 922.8 | 625.5 | 8.63 | 38 | [M+NH_4_]^+^ |
| TG 56:7_FA18:3 | 922.8 | 627.5 | 8.63 | 38 | [M+NH_4_]^+^ |
| TG 56:7_FA20:3 | 922.8 | 599.5 | 8.63 | 38 | [M+NH_4_]^+^ |
| TG 56:7_FA20:4 | 922.8 | 601.5 | 8.63 | 38 | [M+NH_4_]^+^ |
| TG 56:7_FA20:5 | 922.8 | 603.5 | 8.63 | 38 | [M+NH_4_]^+^ |
| TG 56:7_FA22:4 | 922.8 | 573.5 | 8.63 | 38 | [M+NH_4_]^+^ |
| TG 56:7_FA22:5 | 922.8 | 575.5 | 8.63 | 38 | [M+NH_4_]^+^ |
| TG 56:7_FA22:6 | 922.8 | 577.5 | 8.63 | 38 | [M+NH_4_]^+^ |
| TG 56:8_FA16:0 | 920.8 | 647.5 | 8.56 | 38 | [M+NH_4_]^+^ |
| TG 56:8_FA16:1 | 920.8 | 649.5 | 8.56 | 38 | [M+NH_4_]^+^ |
| TG 56:8_FA18:1 | 920.8 | 621.5 | 8.56 | 38 | [M+NH_4_]^+^ |
| TG 56:8_FA18:2 | 920.8 | 623.5 | 8.56 | 38 | [M+NH_4_]^+^ |
| TG 56:8_FA18:3 | 920.8 | 625.5 | 8.56 | 38 | [M+NH_4_]^+^ |
| TG 56:8_FA20:4 | 920.8 | 599.5 | 8.56 | 38 | [M+NH_4_]^+^ |
| TG 56:8_FA20:5 | 920.8 | 601.5 | 8.56 | 38 | [M+NH_4_]^+^ |
| TG 56:8_FA22:5 | 920.8 | 573.5 | 8.56 | 38 | [M+NH_4_]^+^ |
| TG 56:8_FA22:6 | 920.8 | 575.5 | 8.56 | 38 | [M+NH_4_]^+^ |
| TG 56:9_FA18:3 | 918.8 | 623.5 | 8.3 | 38 | [M+NH_4_]^+^ |
| TG 56:9_FA20:4 | 918.8 | 597.5 | 8.3 | 38 | [M+NH_4_]^+^ |
| TG 56:9_FA20:5 | 918.8 | 599.5 | 8.3 | 38 | [M+NH_4_]^+^ |
| TG 56:9_FA22:6 | 918.8 | 573.5 | 8.3 | 38 | [M+NH_4_]^+^ |
| TG 58:10_FA18:2 | 944.8 | 647.5 | 8.4 | 38 | [M+NH_4_]^+^ |
| TG 58:10_FA20:4 | 944.8 | 623.5 | 8.4 | 38 | [M+NH_4_]^+^ |
| TG 58:10_FA20:5 | 944.8 | 625.5 | 8.4 | 38 | [M+NH_4_]^+^ |
| TG 58:10_FA22:5 | 944.8 | 597.5 | 8.4 | 38 | [M+NH_4_]^+^ |
| TG 58:10_FA22:6 | 944.8 | 599.5 | 8.4 | 38 | [M+NH_4_]^+^ |
| TG 58:2_FA18:1 | 960.9 | 661.6 | 9.9 | 38 | [M+NH_4_]^+^ |
| TG 58:3_FA18:1 | 958.9 | 659.6 | 9.7 | 38 | [M+NH_4_]^+^ |
| TG 58:5_FA18:1 | 954.9 | 655.6 | 9.3 | 38 | [M+NH_4_]^+^ |
| TG 58:6_FA16:0 | 952.8 | 679.5 | 9.1 | 38 | [M+NH_4_]^+^ |
| TG 58:6_FA18:0 | 952.8 | 651.5 | 9.1 | 38 | [M+NH_4_]^+^ |
| TG 58:6_FA18:1 | 952.8 | 653.5 | 9.1 | 38 | [M+NH_4_]^+^ |
| TG 58:6_FA20:4 | 952.8 | 631.5 | 9.1 | 38 | [M+NH_4_]^+^ |
| TG 58:6_FA22:4 | 952.8 | 603.5 | 9.1 | 38 | [M+NH_4_]^+^ |
| TG 58:6_FA22:5 | 952.8 | 605.5 | 9.1 | 38 | [M+NH_4_]^+^ |
| TG 58:7_FA16:0 | 950.8 | 677.5 | 8.9 | 38 | [M+NH_4_]^+^ |
| TG 58:7_FA18:0 | 950.8 | 649.5 | 8.9 | 38 | [M+NH_4_]^+^ |
| TG 58:7_FA18:1 | 950.8 | 651.5 | 8.9 | 38 | [M+NH_4_]^+^ |
| TG 58:7_FA18:2 | 950.8 | 653.5 | 8.9 | 38 | [M+NH_4_]^+^ |
| TG 58:7_FA20:4 | 950.8 | 629.5 | 8.9 | 38 | [M+NH_4_]^+^ |
| TG 58:7_FA22:4 | 950.8 | 601.5 | 8.9 | 38 | [M+NH_4_]^+^ |
| TG 58:7_FA22:5 | 950.8 | 603.5 | 8.9 | 38 | [M+NH_4_]^+^ |
| TG 58:7_FA22:6 | 950.8 | 605.5 | 8.9 | 38 | [M+NH_4_]^+^ |
| TG 58:8_FA18:1 | 948.8 | 649.5 | 8.7 | 38 | [M+NH_4_]^+^ |
| TG 58:8_FA18:2 | 948.8 | 651.5 | 8.7 | 38 | [M+NH_4_]^+^ |
| TG 58:8_FA20:3 | 948.8 | 625.5 | 8.7 | 38 | [M+NH_4_]^+^ |
| TG 58:8_FA20:4 | 948.8 | 627.5 | 8.7 | 38 | [M+NH_4_]^+^ |
| TG 58:8_FA22:5 | 948.8 | 601.5 | 8.7 | 38 | [M+NH_4_]^+^ |
| TG 58:8_FA22:6 | 948.8 | 603.5 | 8.7 | 38 | [M+NH_4_]^+^ |
| TG 58:9_FA18:1 | 946.8 | 647.5 | 8.6 | 38 | [M+NH_4_]^+^ |
| TG 58:9_FA18:2 | 946.8 | 649.5 | 8.6 | 38 | [M+NH_4_]^+^ |
| TG 58:9_FA20:4 | 946.8 | 625.5 | 8.6 | 38 | [M+NH_4_]^+^ |
| TG 58:9_FA22:5 | 946.8 | 599.5 | 8.6 | 38 | [M+NH_4_]^+^ |
| TG 58:9_FA22:6 | 946.8 | 601.5 | 8.6 | 38 | [M+NH_4_]^+^ |
| TG 60:10_FA22:5 | 972.8 | 625.5 | 8.6 | 38 | [M+NH_4_]^+^ |
| TG 60:10_FA22:6 | 972.8 | 627.5 | 8.6 | 38 | [M+NH_4_]^+^ |
| TG 60:11_FA22:6 | 970.8 | 625.5 | 8.4 | 38 | [M+NH_4_]^+^ |
